# Patient-Centric Structural Determinants of Adherence Rates Among Asthma Populations: Exploring the Potential of Patient Activation and Encouragement Tool TRUSTR to Improve Adherence

**DOI:** 10.36469/jheor.2020.13607

**Published:** 2020-07-15

**Authors:** Asim Zia, Arthur Brassart, Sheila Thomas, Fen Ye, Judith J. Stephenson, C. Daniel Mullins, Christopher A. Jones

**Affiliations:** 1 Community Development and Applied Economics & Computer Science, University of Vermont, Burlington, VT, USA; 2 trUSX, Inc., South Burlington, VT, USA; 3 Sanofi, Inc., Bridgewater, NJ, USA; 4 HealthCore Inc., Wilmington, DE, USA; 5 Pharmaceutical Health Services Research Department, University of Maryland School of Pharmacy Baltimore, MD, USA

**Keywords:** respiratory diseases, adherence rates, behavioral nudging, rewards, structural models, mobile health apps

## Abstract

**Background:**

Lack of adherence with prescribed medications among the asthma populations exacerbates health outcomes and increases social and economic costs.

**Objectives:**

The proposed study aims to model patient-centric structural determinants of adherence rates among asthma patients and explore the potential of mobile health apps such as the TRUSTR platform to improve adherence using its power of monetary and non-monetary chatbotting and non-non-monetary nudges. Following specific hypotheses are tested: (1) Patient attributes, such as their age and monetary medical condition, have significant effect on their adherence with the prescribed treatment plans. (2) Behavioral nudging with rewards and engagement via mobile health apps will increase adherence rates.

**Methods:**

The patient population (N = 37 359) consists of commercially insured patients with asthma who have been identified from administrative claims in the HealthCore Integrated Research Database (HIRD) between April 1, 2018 and March 31, 2019. Two Structural Equation Models (SEMs) are estimated to quantify direct, indirect, and total effect sizes of age and medical condition on proportion of days covered (PDC) and medical possession ratio (MPR), mediated by patient medical and pharmacy visits. Fourteen additional SEMs were estimated to lateralize TRUSTR findings and conduct sensitivity analysis.

**Results:**

HIRD data reveal mean adherence rate of 59% (standard deviation (SD) 29%) for PDC and 58% for MPR (SD 36%). Key structural findings from SEMs derived from the HIRD dataset indicate that each additional year in the age of the patient has a positive total effect on the adherence rate. Patients with poor medical condition are likely to have lower adherence rate, but this direct effect is countered by mediating variables. Further, each additional reward and higher engagement with a mobile app is likely to have a positive total effect on increasing the adherence rate.

**Conclusions:**

HIRD data reveal mean adherence rate of 59% (SD 29%), providing the evidence for the opportunity to increase adherence rate by around 40%. Statistical modeling results reveal structural determinants, such as the opportunity to nudge, are higher among younger patients, as they have higher probability of being non-adherent. Methodologically, lateralization approach demonstrates the potential to capture real-world evidence beyond clinical data and merge it with clinical data.

## BACKGROUND AND OBJECTIVES

Asthma is reported to affect 20.4 million adults over 18 in the US representing 8.3% of the adult population, with the number of newly diagnosed cases rising annually (+28% between 2001 to 2011). Nearly 65% of adults with current asthma have persistent asthma accounting for considerable asthma morbidity, mortality, and costs. Each day, 11 Americans die from asthma, and in 2015, 3615 people died from asthma. Adults are four times more likely to die from asthma than children.^1^ Lack of adherence with the prescribed medications by asthma patients is a significant factor in increasing mortality rates from asthma.^2^–^6^ Recent systematic review studies, however, have identified that there are significant gaps in understanding behavioral and socio-economic drivers of non-adherence with prescribed medications.^7^,^8^

While more quantitative studies are needed to understand the impact of non-adherence on the survival probabilities of asthma patients, improved understanding of the patient-centric structural determinants of adherence to prescribed treatments may provide actionable information to policy makers and relevant public and private sector stakeholders about the leverage points to maximize adherence rates among asthma patients accounting for their age, medical condition and the process of treatment (ER, hospitalization, specialist etc.). Furthermore, testing the impact of novel interventions to improve adherence rates, such as behavioral nudging through mobile health applications, could be more rigorously evaluated if the patient-centric structural determinants of variable adherence rates among asthma patients are better understood.

Fewer than 10% of all asthma clinical trials have included patient reported outcomes (PROs) while delivering more patient-centered care is the common objective of the industry. To reach the highest standard of patient-centered care, the deployment of engagement solutions, including the collection of PROs and patient insights during treatments, must be a priority. Combining objective and subjective measures can capture patient insights and experiences and predict patients’ long-term treatment outcomes, health status, quality of life, and provide a better understanding of the parameters of adherence for each patient. While engaging with patients during treatment has been a challenge in the past, new health mobile app technologies like TRUSTR can easily engage with patients during their treatment with very high response rates, and a continuous flow of both self-reported and wearable-corroborated data with AI chatbotting, nudge theory, and conversational surveys. This study aims to estimate the capacity of such technology to engage with patients, collect patient insights and PROs, and improve adherence in a real-world setting accounting for patient-centric structural determinants of adherence rates.

Text messages have been shown to be highly effective and cost-effective at reaching patients outside of the hospital setting. Recently, there has been great interest in the “beyond the pill” or “beyond the point of infusion” area of the conversational patient experience. These include daily reminders to stay adherent or the offering of personalized motivational texts that can include words of recognition. Each of these touch points can augment treatment experience in a positive way, such as improving adherence rates, to be interpreted in their aggregate toward a wide range of value added for public and private sectors. Han and Lee^9^ conducted a systematic review of 20 randomized, controlled trial (RCT) studies to examine the effectiveness of mobile health applications in changing health related behaviors and clinical health outcomes. They found that 16 out of these 20 studies reported a positive impact on the targeted health behavior or clinical health outcomes. These RCT studies focused on evaluating the impact of mobile health apps on changing health related behaviors such as physical activity,^10^,^11^ adherence rate,^12^,^13^ dietary change,^14^ weight loss,^15^,^16^ smoking cessation,^17^ and alcohol addiction.^18^ Another meta-analysis study^19^ evaluated 9 RCTs with 1159 subjects to quantify efficacy of app-based interventions designed to support medication adherence and investigate which behavior change techniques used by the apps are associated with efficacy. In the sampled RCTs, health conditions of target populations included cardiovascular disease, depression, Parkinson’s disease, psoriasis, and multimorbidity. This study found that patients who use mobile apps to support them in taking medications are more likely to self-report adherence to medications. However, the meta-regression of behavioral change techniques did not reveal any significant associations with the effect size.

In this evolving socio-technological context, alongside text conversations, identifying activity levels using wearable tracking devices highlights an opportunity to collect persistent, intelligent patient reported outcomes (iPROs) by merging conversational chatbots with wearable-corroborated measurements. Continuous motivated adherence to medication and early reporting of treatable side effects are high priority areas of interest. Mobile health apps such as TRUSTR can potentially measure the treatment experience using the power of chatbotting, nudge theory, conversational surveys, and wearable-corroborated data in a real-world setting.

This study models patient-centric structural determinants of adherence rates among asthma patients and explores the potential of mobile health apps such as TRUSTR platform to improve adherence using its power of chatbotting, monetary and non-monetary nudges, and conversational surveys. The identification of key variables and tendencies will help practitioners describe, understand, and predict the outcomes of a real-world setting use of TRUSTR in the population of interest. It will also yield insights into ways by which to improve patient adherence and patient satisfaction. The following two specific hypotheses are tested in this study:

1. Patient attributes, such as age and medical condition, have significant effect on their adherence with the prescribed treatment plans:
  - Older patients are more likely to have higher adherence rates than younger patients and
  - Patients suffering from severe medical conditions are likely to have lower adherence rate, and
2. Compared with a non-TRUSTR lateralized database control, behavioral nudging with rewards and engagement through chatbotting through mobile health apps such as TRUSTR will increase adherence rates.

## METHODS

### Measurement of Adherence Rates

Adherence rate is generally measured using two approaches: medical possession ratio (MPR)^20^–^22^ and proportion of days covered (PDC).^6^,^23^–^25^ There is no single statistical definition for MPR and various MPR calculations have been developed and discussed in the literature.^26^ The MPR value determines the proportion of days of medication supply within a time interval and both fixed and variable time intervals have been used. We used a variable time interval to accommodate covariance analysis described in SEM methodology. Specific definitions of MPR and PDC used in this study are provided as follows.

Medical possession ratio (MPR) is defined as the total number of treated days in the specified time period divided by total number of days from first treated day until the last treated day (including the last Rx day supply). In this study, MPR is only calculated among patients that have two or more fills with the specified medications over the specified period; MPR of patients with only one fill with the specified medications is coded as ‘0’.

$$MPR=\frac{total\;Rx\;days\;of\;supply}{total\;days\;in\;observation\;period\;from\;first\;until\;last\;treated\;day}$$

PDC uses all available data for a given patient (i.e. from index until the end of continuous eligibility) and is calculated as the total number of covered days on a medication during the time period from index until the end of study observation period, divided by the total number of days in observation period from first until last treated day.

$$PDC=\frac{total\;covered\;days\;on\;drug(s)\;in\;study\;period}{total\;days\;in\;observation\;period\;from\;first\;until\;last\;treated\;day}$$

We used both measures, MPR and PDC, with variable time measures to estimate the adherence rate in this study.

### Patient-Centric Data of Adherence Rates Among Asthma Patients

The data source for this study is the HealthCore Integrated Research Database^®^ (HIRD). The HIRD is a large administrative health care database maintained by HealthCore for use in health outcomes and pharmacoepidemiologic research. The HIRD contains a broad, clinically rich and geographically diverse spectrum of longitudinal medical and pharmacy claims data from 14 Anthem-affiliated health plans in the Northeastern, Mid-Atlantic, Southeastern, Midwest, Central, and Western regions of the United States and represents members in each of the 50 states. The database includes lines of business such as health maintenance organizations, point of service plans, preferred provider organizations, Medicare Advantage, consumer directed health plans, and indemnity plans. HIRD data on over 49 million patients are available from January 1, 2006, for all of the plans represented in the database.

For the HIRD sample presented in this study, the patient identification period is from April 1, 2018 to March 31, 2019 and patients are required to have continuous enrollment during that period. The sampled patient population consists of commercially insured patients with asthma who have been identified from administrative claims in the HIRD between April 1, 2018 to March 31, 2019 (patient identification period) and meet the following inclusion criteria:

1. At least one medical claim with an ICD-10-CM diagnosis code for moderate or severe persistent asthma (J45.4x or J45.5x) during the patient identification period from April 1, 2018, to March 31, 2019,
2. At least one pharmacy claim for an inhaled corticosteroid (ICS) or at least one pharmacy claim for a long-acting beta-2 adrenergic agonist (LABA) or at least one pharmacy claim for an ICS/LABA combo medication (See Supplementary Materials, Table S2 for specific medication names and generic product identifier codes),
3. Have only commercial health insurance during April 1, 2018, to March 31, 2019,
4. Are 18 years of age or older as of the end of the patient identification period (March 31, 2019), and
5. Have at least 12 months of continuous enrollment with both medical and pharmacy benefits during the patient identification period.

The number of patients, means, SD, standard errors (SE), medians, correlation and covariance matrices were provided for ten asthma-related claims-determined variables described in Table 1 for the patient identification period from April 1, 2018, to March 31, 2019. All study measures of interest were described using univariate statistics consisting of number of patients, mean, SD, SE, and median. The patient cohort was limited to patients with at least one medical claim with an ICD-10-CM asthma diagnosis in any position and at least one pharmacy claim for an ICS, LABA, or ICS/LABA combo medication in the 12 month period from April 1, 2018 to March 31, 2019. The correlation and covariance matrices for the study measures were determined for the identified patient cohort; all study variables were considered to be continuous and there were no missing values for any of them. All variables were included, except inpatient length of stay (LOS), which was presented descriptively since it was calculated only for those patients with at least one inpatient hospitalization. Table 2 and Table 3 show means, variances, and covariances of 10 variables that were used to estimate Summary Statistics Data (SSD) in STATA 15.

### SEMs Estimated from Summary Statistics Databases (SSDs)

STATA 15 SSD commands were used to reconstruct the dataset and measure 16 SEMs. SEMs #1 and #2 were measured to predict MPR and PDC respectively directly from the HIRD dataset. Data analysis was performed using structural equation modeling logarithms in STATA 15 for Windows (StataCorp LLC, College Station, TX) specifically following SEM algorithms developed by Acock^28^ and Ullman and Bentler.^29^

Goodness-of-fit statistics were used to determine the fit of the estimated SEMs to the sample data. Two approaches to measure the goodness of fit were utilized for evaluating the model fit of estimated SEMs: Standardized Root Mean-squared Residual (SRMR) and Coefficient of Determination (CD). Ideally, the best fit models have SRMR closer to zero and CD closer to one. SRMR and CD for all 16 SEMs are reported in the Supplementary Materials. Since SEMs were derived from SSD, we could not use other statistics (e.g., likelihood ratio tests, comparative fit index, Tucker-Lewis index) to evaluate the model fitness.

Lateralization of TRUSTR Social Media Experimental Findings to Modeling Patient-Centric Adherence Rates

Tables 2 and 3 show the addition of two variables (shown in red font), rewards [measured as $/month] and engagement rate [measured as text messages/month] that were lateralized from TRUSTR social media study (see Zia et al^27^) to explore the potential of mobile health apps on adherence rates. We assume that rewards are distributed with a mean of $30 per month and SD of $15. We assume baseline covariance rates of 0.7 and 0.6 between rewards and MPR, and PDC respectively. Further, we assume that respondents send a mean of 234 text messages per month with an SD of 180 messages per month. Consistent with the social media study, we assume a high covariance of 0.82 between rewards and engagement rate. We also assume baseline covariance rates of 0.5 and 0.46 between engagement rate and MPR, and PDC respectively. Four sensitivity analysis scenarios are also tested, with two scenarios (S1 and S2) representing 10% and 20% increases in covariance of rewards and engagement rate with adherence rate measures (MPR and PDC), and two scenarios representing 10% and 20% decreases in covariance of rewards and engagement rate with adherence rate measures (See Table 4 for scenarios representing sensitivity analysis). SEMs #3 and #4 measure the effect of rewards on MPR and PDC respectively; SEMs #5 and #6 measure the effect of engagement rate on MPR and PDC respectively; and SEMs #7 and #8 measure the effect of both rewards and engagement rate on MPR and PDC respectively. Finally, we present results of sensitivity analysis through eight additional SEMs, four of which vary covariance between rewards and engagement rate vis-à-vis adherence rate measure of MPR, and the other four for PDC by 10% and 20% increases or decreases in covariance, compared with baseline covariance rates assumed in SEMs #7 and #8.

## RESULTS

Estimated SEMs for eight models are presented in the Supplementary Material (Appendix 2). The direct, indirect, and total effects of each SEM are also presented in the Supplementary Material (Appendix 2). Furthermore, results from eight additional SEMs estimated for four sensitivity analysis scenarios are reported in the Supplementary Material (Appendix 3), and their highlights are also summarized. Next, we focus on presenting the key findings with respect to the study objectives and hypotheses.

**Figure 1:**
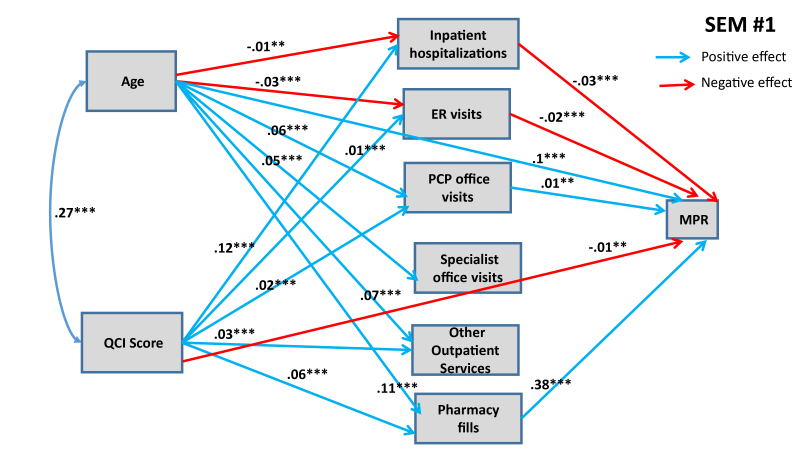
SEM #1 Presents Structural Determinants of MPR from HIRD Data Only statistically significant paths are shown. * 90% confidence level; ** 95% confidence level; *** 99% confidence level

**Figure 2:**
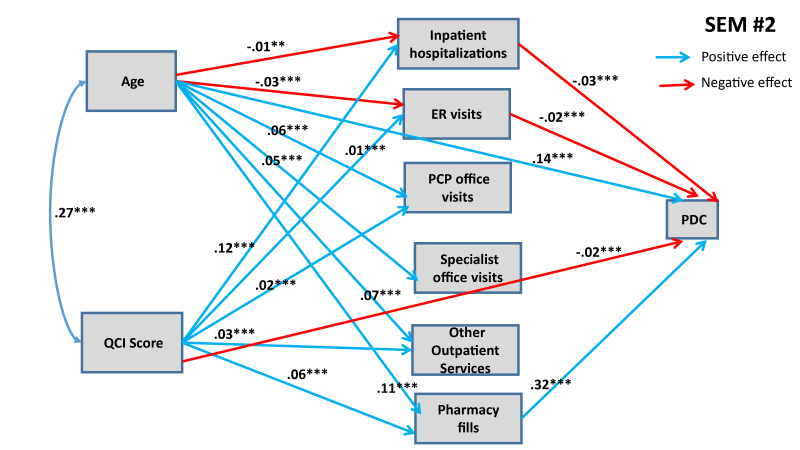
SEM #2 Presents Structural Determinants of PDC from HIRD Data Only statistically significant paths are shown. * 90% confidence level; ** 95% confidence level; *** 99% confidence level

Figure 1 and Figure 2 show statistically significant paths estimated for SEMs 1 and 2, respectively.

Both of these SEMs show confounding effects of patient age and QCI score on MPR and PDC. Both models predict that each additional year in the age of the patient has a direct positive effect on the adherence rate. Age also influences adherence rate through various indirect pathways, as shown in Figures 1 and 2. Older people are less likely to have inpatient hospitalizations and ER visits (connected with asthma), and asthma patients going through inpatient hospitalizations and ER visits are likely to have lower adherence rates (both MPR and PDC). Conversely, older people are more likely to maintain their pharmacy fills, which in turn has a positive effect on adherence rates. Further, we also find that older people are more likely to engage in PCP office visits, specialist office visits, and utilize other outpatient services, but we do not detect any significant effect of these mediating variables on adherence rates (with the exception of PCP office visits having positive effect on MPR). When we combine direct and indirect effects, age has consistently positive effect on adherence rate, implying that younger people are less likely to adhere with prescribed asthma medications (see Table 5 for total effects of eight SEMs).

Similarly, medical condition of the patients, measured through QCI, also have confounding effects on adherence rates, mediated through variegated health management pathways. Structural analysis in Figures 1 and 2 also shows that, as expected, patients with worse medical conditions are more likely to engage in inpatient hospitalizations, ER visits, PCP office visits and other outpatient services, which in turn have differential effects on adherence rates. The direct effects of medical conditions on adherence rates are statistically significant in both SEMs: patients with worse medical conditions are less likely to adhere. Overall, from total effects analysis shown in Table 5, we find that patients with worse medical condition (depicted by higher QCI score) have lower adherence rate (significant for PDC at p<0.05, but not significant for MPR).

**Figure 3:**
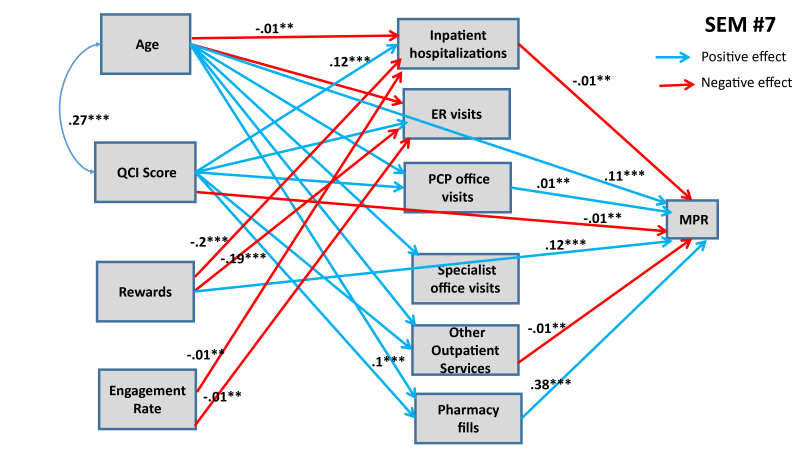
SEM #7 Presents Structural Determinants of MPR from HIRD Data and TRUSTR Social Media Experimental Data Only statistically significant paths are shown. * 90% confidence level; ** 95% confidence level; *** 99% confidence level

**Figure 4:**
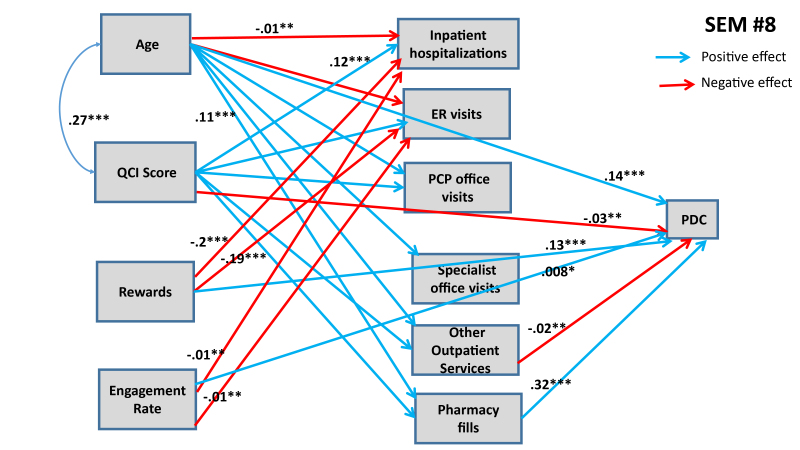
SEM #8 Presents Structural Determinants of PDC from HIRD Data and TRUSTR Social Media Experimental Data Only statistically significant paths are shown. * 90% confidence level; ** 95% confidence level; *** 99% confidence level

Six additional SEMs were estimated to lateralize TRUSTR findings (i.e., “what if” scenarios tested to quantify impacts of TRUSTR rewards and engagement nudges on behavioral change from one population (social media) to another population (HIRD)). Supplementary Materials show results for SEMs #3 through #6. Here, in Figure 3 and Figure 4 we show results from SEMs #7 and #8, each of which presents structural determinants for variables derived from HIRD data set as well as TRUSTR variables (rewards and engagement rate) that are hypothesized to influence adherence rates as mediated through health management pathways. Table 5 presents total effects for all eight SEMs. Further, sensitivity analysis around lateralization assumptions were conducted with eight additional SEMs, results of which are shown in Table 6.

From Figures 3 and 4 and Table 5, we find that with lateralization, age has a consistently significant positive effect size across both MPR and PDC; however, QCI score has a negative effect only for PDC. Importantly, we find that each additional monetary reward increases the likelihood of adherence rate. The total effect of engagement rate is marginally positive for PDC but insignificant for MPR. Further, sensitivity analysis results shown in Table 6 demonstrate that generally the findings about structural determinants of adherence rates are robust across SEMs. The effects of rewards and engagement rate however predictably vary upward and downward, when we marginally increase (SEMs #9 through #12) or marginally decrease (SEMs #13 through #16) the covariance between rewards and adherence rates or engagement rates and adherence rates. Remaining structural determinants of adherence rates retain the direction and magnitude of their effects, as estimated in SEMs #1 through #8.

## DISCUSSION

HIRD data reveal a mean adherence rate of 59% (SD 29%), providing the evidence for the opportunity to increase adherence rate by around 40%. Specifically, HIRD data reveal mean adherence rate of 59% (SD 29%) for PDC and 58% for MPR (SD 36%). While PDC is typically considered to be more conservative estimate of medication adherence rate compared with MPR, the HIRD dataset reveals statistically similar mean value. The SD of MPR is however higher than PDC. The reason the mean MPR is slightly less than the mean PDC is because of the statistical definition of MPR that we used. As indicated earlier, there is no single statistical definition for MPR and various MPR calculations have been developed and discussed in the literature.^26^ The MPR value determines the proportion of days of medication supply within a time interval and both fixed and variable time intervals have been used. We used a variable time interval—the MPR denominator, is the total number of days from first treated day until the last treated day. This requires at least two fills and patients with only one fill were coded with an MPR of zero so that all 37 359 observations could be used for the covariance calculations. The statistical MPR and PDC definitions that were used are given in Table 1. The effect of the zero MPR’s on the mean reduces the mean MPR so that it is less than the average PDC; however, the median is unaffected by the 0 MPRs and the median MPR (0.66) > median PDC (0.60) (see Table 2).

A key implication of this study is that statistical modeling results reveal structural determinants, which could be utilized to find substantial opportunities for increasing the adherence rates. First, structural analysis of HIRD data set reveals that the opportunity to nudge is higher among younger patients as they have higher probability of being non-adherent. Second, patients with relatively worse medical conditions could also be targeted for improving the overall adherence rates with asthma medications.

Findings about structural determinants of adherence rates are also consistent with previously published empirical literature. For example, in a recent systematic review of 51 published studies focused on evaluating asthma inhaler adherence determinants in adults, Dima et al^7^ found “consistent links between adherence and stronger inhaler-necessity beliefs, and possibly older age.” While our study did not explicitly model inhaler-necessity beliefs, as these are not available in HIRD data set, we found statistically significant effect of age on adherence, consistent with Dima et al.^7^ finding. Similar age effects on adherence rates were found by Feehan et al.^6^ Further, our structural findings about patient medical condition and health management interventions are also consistent with previous literature reviewed by Bårnes and Ulrik.^5^ A review of 19 studies revealed that the mean level of adherence was between 22% and 63%. “Poor adherence was associated with youth, being African-American, having mild asthma, <12 years of formal education, and poor communication with the health-care provider, whereas improved adherence was associated with being prescribed fixed-combination therapy (ICS and long-acting beta-2 agonists). Good adherence was associated with higher FEV1, a lower percentage of eosinophils in sputum, reduction in hospitalizations, less use of oral corticosteroids, and lower mortality rate (Bårnes and Ulrik^5^).”

Lateralization of structural findings from HIRD dataset, when merged with TRUSTR social media experimental study, identify potential pathways to capitalize on the opportunities available for targeting younger asthma patients and/or patients in relatively worse off medical condition. In our lateralization approach, we assumed fairly conservative assumptions, and then conducted sensitivity analysis around those conservative assumptions, and discovered that each additional TRUSTR reward has a positive total effect on nudging the adherence rate to be higher. Since many mobile health app studies have found evidence in the literature that younger people are more likely to use mobile health apps, deployment of mobile health apps to nudge the behaviors of younger people to be more complaint appears very promising.^30^–^35^ An RCT with a mobile health app (e.g., TRUSTR) would be the next logical step to test the potential of nudging younger asthma patients to become more adherent with their medications. In a recently published meta-analysis study, Armitage et al.^19^ found that mobile health apps increase medication adherence rates among the patients suffering from cardiovascular disease, depression, Parkinson’s disease, psoriasis, and multimorbidity. This study shows that similar benefits from mobile health apps could be derived for asthma patients.

The nudging opportunity might not be as straightforward with the people suffering from relatively worse medical condition. Mere provision of rewards might not be enough to overcome the capacity limitations of severe asthmatic patients. Non-monetary nudging through conversational features of mobile health apps could however be tested in follow up RCT. Engaging younger patients through conversational and non-monetary engagement could also be tested in follow up RCTs. Existing telehealth applications fail to reach many patients, and fail to establish crucial elements of digital trust. Moreover, many concerns of patients from a real-world data perspective are missed by surveys that have very narrow response windows. The TRUSTR tool allows for multichannel controlled messaging while inviting crucial feedback in the patient’s own words. Moreover, by co-synchronizing and co-registering with wearables and other applications (WhatsApp, GoogleFit etc.), it is envisaged that TRUSTR can provide controlled and validated information, asynchronously, just as it collects valuable real-world data.

## CONCLUSIONS

This study models patient-centric structural determinants of adherence rates among asthma patients and explores the potential of mobile health apps such as TRUSTR platform to improve adherence. The US nationally representative HIRD data reveal mean adherence rate of 59% (SD 29%) for PDC and 58% for MPR (SD 36%). Key structural findings from SEMs derived from the HIRD dataset indicate that: (1) Each additional year in the age of the patient has a positive total effect on the adherence rate. (2) Patients with worse medical condition (ie, higher QCI score) are likely to have lower adherence rate, but this direct effect is countered in opposite direction by mediating variables. Further, key findings from lateralization of TRUSTR with HIRD data indicate that: (3) Each additional reward has a positive total effect on increasing the adherence rate. (4) Higher engagement rate with TRUSTR app has a weak positive effect on increasing adherence rate. These findings indicate that the opportunity to nudge is higher among younger patients as they have higher probability of being non-adherent. Methodologically, lateralization approach pioneered in this study demonstrates the potential to capture real-world evidence beyond clinical data and merge it with clinical data (e.g., this study lateralizes TRUSTR experimental findings to a sample of 37 359 asthma patients identified from claims in the HealthCore Integrated Research Database^®^ (HIRD) using structural equation modeling and path analysis). Statistical modeling demonstrates the ability to differentiate patient behaviors of interest with more granularity (e.g., by age, medical condition (QCI score), and frequency of visits to ER, specialists, PCPs, and other outpatient services). RCTs with mobile health apps (e.g., TRUSTR) are recommended for future research to test the potential of nudging (asthma) patients to become more adherent with their medications.

## Supplementary Information



## Figures and Tables

**Table 1:** Study Variables

Name in the Protocol	Name in the Model Code	Description
Age	age	Age in years at end of patient identification period.
Inpatient Hospitalizations	inpatient_hospitaliza~s	Number of inpatient hospitalizations (including ER visits that lead to a hospitalization); no inpatient hospitalization is coded as ‘0’ LOS among patients with ≥1 asthma-related hospitalization; aggregated over period of interest (days), mean (SE), median.
ER Visits	er_visits	Number of ER visits; no ER visit is coded as ‘0’.
Primary Care Physician (PCP) [Family Medicine/Practice, Internal Medicine, Gerontologist] Office Visits	pcp_officevisits	Number of Primary Care Physician (PCP) [Family Medicine/Practice, Internal Medicine, Gerontologist] office visits; no visit is coded as ‘0’.
Specialist (Allergy/Immunology, Pulmonology, Respiratory) Office Visits	specialist_office_vis~s	Number of Specialist (Allergy/Immunology, Pulmonology, Respiratory) office visits; no visit is coded as ‘0’.
Other Outpatient Services	other_outpatientservi~s	Number of other outpatient services (e.g., durable medical equipment, imaging, medication and related services, procedures, physical therapy/occupational therapy/speech, physician other services, lab tests, other); no other outpatient services is coded as ‘0’.
Asthma-Related Pharmacy Dispensing	pharmacy_fills	Asthma-related pharmacy dispensing (n[%] of patients with ≥1 prescription fill for all medications listed in Supplementary Material, Table S2; mean [SE]/median of fills among all patients) (refer to Supplementary Material, Table S2 for codes).
Medical Possession Ratio (MPR)	mpr	Medical possession ratio (MPR) for ICS, LABA, and ICS/LABA combo medications (n[%] of patients with ≥1 prescription fill; mean [SE]/median of fills among all patients) (refer to Supplementary Material, Table S2 for codes). MPR is defined as total number of treated days in the specified time period divided by total number of days from first treated day until the last treated day (including the last Rx day supply). MPR is only calculated among patients that have two or more fills with the specified medications over the specified period; MPR of patients with only one fill with the specified medications is coded as ‘0’.
Proportion of Days Covered (PDC)	pdc	Proportion of days covered (PDC) for ICS, LABA, and ICS/LABA combo medications (n[%] of patients with ≥1 prescription fill; mean [SE]/median of fills among all patients) (refer to Supplementary Materail, Table S2 for codes). PDC is defined as total number of treated days in the specified time period divided by total number of days from first treated day until the end of the period. Calculated only among those patients who received one or more of the specified medication(s) over the specified period.
Quan-Charlson Comorbidity Index (QCI) score	qci_score	Quan-Charlson comorbidity index (QCI) score; n of patients; mean (SE), median (refer to Supplementary Material, Table S3 for codes) To quantify comorbidity, the QCI score is computed by adding the weights that are assigned to the specific diagnoses. A score of 1 is attributed to myocardial infarction, congestive heart failure, peripheral vascular disease, cerebrovascular disease, dementia, chronic pulmonary disease, connective tissue/rheumatologic disease, peptic ulcer disease, mild liver disease, and diabetes without chronic complications. The following diseases are scored as 2: hemiplegia or paraplegia, renal disease, diabetes with complications, and malignancy including leukemia and lymphoma. Moderate or severe liver disease is scored 3. Finally, a score of 6 is assigned to metastatic solid carcinoma and AIDS/HIV. Each diagnosis is only counted once (e.g., if a patient has ICD-9 code 410.xx and 412.xx, they will receive a score of 1 for MI, not 2). The minimum possible score is 0 and the maximum possible score is 33.

**Table 2:** Means and Medians for HIRD Data and Two Lateralization Variables, Rewards, and Engagement Rate Derived from the TRUSTR Social Media Study^27^

Variable	Number (n%)	Analysis Sample Size	Mean	SD	SE	Median
Age on 03/31/2019	37 359 (100.0%)	37 359	49.64	14.87	0.0769	52
Inpatient Hospitalizations	1133 (3.0%)	37 359	0.04	0.26	0.0013	0
Length of Stay (LOS) among patients with ≥1 asthma-related hospitalization	1133 (3.0%)	1133	5.61	6.80	0.2020	4
ER Visits	684 (1.8%)	37 359	0.02	0.20	0.0010	0
Out visits	36 789 (98.5%)	37 359	3.18	4.73	0.0245	2
PCP Office Visits	12 883 (34.5%)	37 359	2.18	2.50	0.0129	1
Specialist Office Visits	18 987 (50.8%)	37 359	2.61	3.81	0.0197	2
Other Outpatient Services	26 740 (71.6%)	37 359	2.22	4.41	0.0228	1
Pharmacy Fills (All meds in table 2)	37 359 (100.0%)	37 359	9.95	7.37	0.0381	8
Pharmacy Fill (ICS, LABA, ICS/LABA)	37 359 (100.0%)	37 359				
ICS Monotherapy	10 571 (28.3%)	37 359				
LABA	371 (1.0%)	37 359				
ICS/LABA combination	30 590 (81.9%)	37 359				
Quan-Charlson Comorbidity Index (QCI) score	37 359 (100.0%)	37 359	1.47	1.13	0.0058	1
MPR (ICS, LABA, ICS/LABA)	37 359(100.0%)	37 359	0.58	0.36	0.0019	0.66
PDC (ICS, LABA, ICS/LABA)	37 359 (100.0%)	37 359	0.59	0.29	0.0015	0.60
Reward	37 359	37 359	30	15	0.1	35
Engagement rate (messages/month)	37 359	37 359	234	180	0.18	265

**Table 3:** Variance and Covariances of HIRD and Lateralized Data

	Age	Inpatient Hospitalization	ER Visits	PCP Office Visits	Specialist Office Visits	Outpatient Services	Pharmacy (Asthma meds)	QCI Score	MPR (ICS, LABA, ICS/LABA)	PDC (ICS, LABA, ICS/LABA)	Rewards	Engagement Rate
Age	221.105233	0.089650	−0.094017	2.442816	3.267802	5.864529	14.209445	4.632682	0.803770	0.761121		
Inpatient Hospitalization	0.089650	0.067846	0.011868	0.037303	0.033255	0.113002	0.200045	0.036237	0.000065	−0.000166		
ER Visits	−0.094017	0.011868	0.040578	0.033435	0.022245	0.075032	0.084031	0.002199	−0.001100	−0.000795		
PCP Office Visits	2.451662	0.037385	0.033620	6.244202	6.816085	7.943561	4.400360	0.101140	0.087750	0.047842		
Specialist Office Visits	3.267802	0.033255	0.022245	6.805591	14.548804	15.837080	7.174931	0.076749	0.131478	0.073374		
Outpatient Services	5.864529	0.113002	0.075032	7.918511	15.837080	22.331055	10.065882	0.274862	0.182628	0.105173		
Pharmacy (Asthma meds)	14.209445	0.200045	0.084031	4.385411	7.174931	10.065882	54.346633	0.771759	1.037027	0.708629		
QCI Score	4.632682	0.036237	0.002199	0.101364	0.076749	0.274862	0.771759	1.282943	0.018688	0.012082		
MPR (ICS, LABA, ICS/LABA)	0.803770	0.000065	−0.001100	0.087459	0.131478	0.182628	1.037027	0.018688	0.129239	0.062483		
PDC (ICS, LABA, ICS/LABA)	0.761121	−0.000166	−0.000795	0.047573	0.073374	0.105173	0.708629	0.012082	0.062483	0.082140		
Rewards	−0.2	−0.8	−0.6	0.05	0.1	0.15	0.85	0.002	0.7	0.6	225	
Engagement rate (messages/month)	−0.100000	−0.600000	−0.7	0.08	0.2	0.26	1.04	0.001	0.5	0.46	0.82	32 400

ER=Emergency Room; PCP=Primary Care Physician; QCI=Quan-Charlson Comorbidity Index; MPR=Medication Possession Ratio; PDC=Proportion of Days Covered

ICS=Inhaled corticosteroid; LABA=Long acting beta-2 adrenergic agonist

**Table 4:** Scenarios Representing Sensitivity Analysis on Covariance of Rewards and Engagement Rate With Adherence Rate Measures (MPR and PDC)

Senstivity analysis BASELINE		
	MPR	PDC
Rewards	0.7	0.6
Engagement Rate	0.5	0.46
**Senstivity analysis 10% higher covariance (S1)**		
	**MPR**	**PDC**
Rewards	0.77	0.66
Engagement Rate	0.55	0.506
**Senstivity analysis 20% higher covariance (S2)**		
	**MPR**	**PDC**
Rewards	0.84	0.72
Engagement Rate	0.6	0.552
**Senstivity analysis 10% lower covariance (S3)**		
	**MPR**	**PDC**
Rewards	0.63	0.54
Engagement Rate	0.45	0.414
**Senstivity analysis 20% lower covariance (S4)**		
	**MPR**	**PDC**
Rewards	0.56	0.48
Engagement Rate	0.4	0.368

**Table 5:** Comparison of Total Effects Across Eight SEMs

Predictor	SEM #1 (MPR)	SEM #2 (PDC)	SEM #3 (MPR)	SEM #4 (PDC)	SEM #5 (MPR)	SEM #6 (PDC)	SEM #7 (MPR)	SEM #8 (PDC)
**Inpatient hospitalizations**	−0.0340***	−0.0303***	−0.0120**	−0.0060	−0.0339***	−0.0302***	−0.0120**	−0.0059
**ER visits**	−0.0259***	−0.01914***	−0.0055	0.0034	−0.0258***	−0.0190***	−0.0054	0.0035
**PCP office visits**	0.01583**	−0.0006***	0.0136**	−0.0031	0.0158**	−0.0007	0.0136*	−0.0031
**Specialist office visits**	−0.0107	−0.0093	−0.0014	0.0008	−0.0106	−0.0093	−0.0014	0.0008
**Other Outpatient Services**	−0.0078	−0.0154	−0.0174*	−0.0259**	−0.0078	−0.0154	−0.0174*	−0.0260**
**Pharmacy fills**	0.3844***	0.3293***	0.3812***	0.3257***	0.3844***	0.3293***	0.3812***	0.3257***
**Age**	0.1486***	0.1451***	0.1489***	0.1818***	0.1486	0.1815***	0.1489***	0.1818***
**QCI Score**	0.0048	−0.0283***	0.0048	−0.0129**	0.0048965	−0.0128**	0.0048	−0.0129**
**Rewards**	N/A	N/A	0.1297***	0.1393***	N/A	N/A	0.1297***	0.1393***
**Engagement Rate**	N/A	N/A	N/A	N/A	0.0077	0.0088*	0.0076	0.0088*

* 90% confidence level;

** 95% confidence level;

*** 99% confidence level

**Table 6:** Sensitivity Analysis: Total Effects on Adherence Rate for Four Scenarios

Predictor	SEM #9 (MPR) S1: 10% ↑	SEM #10 (PDC) S1: 10% ↑	SEM #11 (MPR) S2: 20% ↑	SEM #12 (PDC) S2: 20% ↑	SEM #13 (MPR) S3: 10% ↓	SEM #14 (PDC) S3: 10% ↓	SEM #15 (MPR) S4: 20% ↓	SEM #16 (PDC) S4: 20% ↓
**Inpatient hospitalizations**	−0.0095*	−0.0032	−0.007	−0.0005	−0.0145**	−0.0086*	−0.0169***	−0.0112**
**ER visits**	−0.0030	0.0060	−0.0007	0.0085*	−0.0077	0.0010	−0.0010**	−0.0014
**PCP office visits**	0.0134**	−0.0033	0.0131*	−0.0036	0.0139**	−0.0028	0.0141**	−0.0025
**Specialist office visits**	−0.0003	0.0019	0.0006	0.0031	−0.0024	−0.0002	−0.0035	−0.0013
**Other Outpatient Services**	−0.0185*	−0.02720**	−0.0196*	−0.0283**	−0.0163	−0.0248**	−0.0152	−0.0237**
**Pharmacy fills**	0.3809***	0.3253***	0.3805***	0.3249***	0.3816***	0.3261***	0.3819***	0.3265***
**Age**	0.1489***	0.1818***	0.1489***	0.1818***	0.1488***	0.1817***	0.1488***	0.1817***
**QCI Score**	0.0048	−0.0129**	0.0048	−0.0129**	0.0048	−0.0128**	0.0048	−0.0128**
**Rewards**	0.1427***	0.1532***	0.1557***	0.1672***	0.1167***	0.1254***	0.1038***	0.1115***
**Engagement Rate**	0.0084*	0.0097*	0.0092*	0.0106**	0.0069	0.0079	0.0061	0.0070

* 90% confidence level;

** 95% confidence level;

*** 99% confidence level
