# Supplementary material for: Patient-Centric Structural Determinants of Adherence Rates Among Asthma Populations: Exploring the Potential of Patient Activation and Encouragement Tool TRUSTR to Improve Adherence
Source: J Health Econ Outcomes Res. 2020 Jul 15;7(2):111–22. doi: 10.36469/jheor.2020.13607 (PMC7398613; doi:10.36469/jheor.2020.13607)
Supplement: Supplementary file 1 [file jheor-7-2-13607-s01.pdf]

Journal of Health Economics and Outcomes Research

Supplementary Online Material

Zia A, Brassart A, Thomas S, et al. Patient-centric structural determinants of adherence rates among asthma populations: exploring the potential of patient activation and encouragement tool TRUSTR to improve adherence. *JHEOR*. 2020:7(2);111-122.
doi: 10.36469/jheor.2020.13607

Appendix 1: Asthma Code Lists; QCI Coding Algorithm
Table S1. Diagnosis Codes for Moderate or Severe Persistent Asthma
Table S2. Asthma Medication Code List
Table S3. Coding algorithm for Quan-Charlson Comorbidity Score

Appendix 2: Detailed Outputs of eight Structural Equation Models and Estimated Direct, Indirect and Total Effects

Appendix 3: Sensitivity Analysis Results Presenting Detailed Outputs of Eight Additional Structural Equation Models and Their Estimated Direct, Indirect and Total Effects

This supplementary material has been provided by the authors to give readers additional information about their work.

This is an open-access article distributed under the terms of the Creative Commons Attribution 4.0 International License (CCBY-4.0). View this license's legal deed at http://creativecommons.org/licenses/by/4.0 and legal code at http://creativecommons.org/licenses/by/4.0/legalcode for more information.

Appendix 1: Asthma Code Lists; QCI Coding Algorithm

Table S1. Diagnosis Codes for Moderate or Severe Persistent Asthma

Diagnosis code	ICD-10-CM: J45.4x or J45.5x

Table S2. Asthma Medication Code List

Drug	GPI Code (starting with)	HCPCS Code	ICD-9-CM Procedure Code	ICD-10-PCS
Asthma controller medications				
**Inhaled corticosteroids (ICS)**				
Beclomethasone	44400010	J7622		
Budesonide	44400015	J7626, J7627, J7633, J7634		
Ciclesonide	44400017			
Flunisolide	44400030	J7641		
Fluticasone	44400033			
Mometasone	44400036			
Triamcinolone	44400040	J7683, J7684		
**Long acting beta-2 adrenergic agonist (LABA)**				
Salmeterol	44201058x			
Formoterol	44201027x	J7606, J7640, Q4099		
Arformoterol	44201012102520	J7605		
**ICS/LABA combination**				
Budesonide + formoterol	442099024x			
Fluticasone + formoterol				
Fluticasone + salmeterol	4420990270x			
Fluticasone+ Vilanterol	44209902758020, 44209902758030			
Mometasone furoate+ formoterol	4420990290x			
**Leukotriene modifiers**	4450x			
Zileuton	44504085x			
Leukotriene receptor antagonist (LTRA)				
Montelukast	44505050x			
Zafirlukast	44505080x			
**Methylxanthine**				
Aminophylline	44300010x	J0280		
Dyphylline	44300020x, 44993204200310, 4499100220	J1180		
Oxtriphylline	44300030x, 449910023			
Theophylline	4430004000, 4430004001, 4499100240, 4499100242, 4499100250, 4499300322, 4499320440, 4499900305, 44992203, 44999602	J2810		
**Monoclonal antibodies***				
Omalizumab	44603060002120	J2357		
Mepolizumab	44604055002120	C9473, J2182		

Table S2. Asthma Medication Code List

Drug	GPI Code (starting with)	HCPCS Code	ICD-9-CM Procedure Code	ICD-10-PCS
Reslizumab	44604460002020	J2786		
Benralizumab	4460402000E520	C9466		
**Long acting muscarinic antagonists (LAMA)**				
Tiotropium Bromide (Spiriva)	44100080x			
**Mast cell stabilizers**				
Cromolyn	4415001010 excluding 44150010102900	J7630, J7631, J7632		
**Long acting albuterol**	44201010107410, 44201010107420, 44201010107470, 44201010107480, 44201010100410, 44201010100470, 44201010100480			
Asthma rescue medications				
**Short acting beta-2 adrenergic agonist (SABA)**				
Inhaled SABA				
Albuterol	44201010003405, 44201010102100, 44201010102515, 44201010102520, 44201010102555, 44201010102560, 44201010103410, 44201010108020	J7602, J7603, J7609, J7610, J7611, J7613, J7616, J7618, J7619, J7621, J7625, Q4093, Q4094		
Bitolterol	44201020x	J7628, J7629		
Levalbuterol	44201045x	J7607, J7612, J7614, J7615, J7617		
Metaproterenol	44201050202503, 44201050202505, 44201050202510, 44201050203310	J7667-J7670, J7672, J7675		
Pirbuterol	44201055x			
Terbutaline	44201060203405	J7680, J7681		
Isoetharine	44201030x	J7647-J7655		
Isoproterenol	44201040102520	J7657-J7660		
	44201040102525			
	44201040102530			
	44201040103405			
	44201040102510			
	44201040103410			
	44201040203405			
	44209902103410			
Non-inhaled SABA				
Albuterol	44201010100120, 44201010100305, 44201010100310, 44201010101205			

Table S2. Asthma Medication Code List

Drug	GPI Code (starting with)	HCPCS Code	ICD-9-CM Procedure Code	ICD-10-PCS
Isoproterenol	44201040100705, 44201040100710, 44201040102005			
Metaproterenol	44201050200305, 44201050200310, 44201050201205			
Terbutaline	44201060200305, 44201060200310, 44201060202005	J3105		
**Oral corticosteroids (OCS)**				
Prednisone	22100045000305, 22100045000310, 22100045000315, 22100045000320, 22100045000325, 22100045000330, 22100045000335, 22100045001205, 22100045001310, 22100045002005, 22100045002010, 22100045002015, 22100045006405, 22100045006410			
Prednisolone	22100040000305, 22100040001203, 22100040001205, 22100040006420, 22100040200910, 22100040202020, 22100040202040, 22100040202060, 22100040207215, 22100040207220, 22100040207240, 22100040202050, 22100040202025, 22100040101860			
Methylprednisolone	22100030000305, 22100030000310, 22100030000315, 22100030000320, 22100030000325, 22100030000330, 22100030006405, 22100030006410			
Paramethasone	22100035100305, 22100035100310			
Hydrocortisone	22100025000303, 22100025000305, 22100025000310, 22100025201810			

Table S2. Asthma Medication Code List

Drug	GPI Code (starting with)	HCPCS Code	ICD-9-CM Procedure Code	ICD-10-PCS
Dexamethasone	22100020000310, 22100020000315, 22100020000320, 22100020000325, 22100020000330, 22100020000335, 22100020000340, 22100020000345, 22100020001005, 22100020001320, 22100020002005, 22100020002010, 22100020006400, 22100020006420			
Betamethasone	22100010000305, 22100010002010			
Budesonide	22100012007530, 22100012007020			
Cortisone Acetate	22100015100303, 22100015100305, 22100015100310			
Triamcinolone	22100050000305, 22100050000310, 22100050000315, 22100050000320, 22100050006405, 22100050201203, 22100050201205			
**SAMA and SAMA combinations**				
Ipratropium (Atrovent)	42300040102010, 44100030102020, 44100030103410, 44100030123420	J7644, J7645		
Ipratropium/albuterol (Combivent or DuoNeb)	44209902013220, 44209902013420, 44209902012015	J7620		

Table S3. Coding algorithm for Quan-Charlson Comorbidity Score

Comorbidities	ICD-9-CM Diagnosis Codes	ICD-10-CM Diagnosis Codes	Charlson Score
**Myocardial infarction**	410.x, 412.x	I21.x, I22.x, I25.2	1
**Congestive heart failure**	398.91, 402.01, 402.11, 402.91, 404.01, 404.03, 404.11, 404.13, 404.91, 404.93, 425.4-425.9, 428.x	I09.9,I11.0, I13.0, I13.2, I25.5, I42.0, I42.5-I42.9, I43.x, I50.x, P29.0	1
**Peripheral vascular disease**	093.0, 437.3, 440.x, 441.x, 443.1-443.9, 447.1, 557.1,557.9, V43.4	I70.x, I71.x, I73.1, I73.8, I73.9, I77.1,I79.0, I79.2, K55.1, K55.8, K55.9,Z95.8, Z95.9	1
**Cerebrovascular disease**	362.34, 430.x-438.x	G45.x, G46.x, H34.0, I60.x-I69.x	1
**Dementia**	290.x, 294.1, 331.2	F00.x-F03.x, F05.1, G30.x, G31.1	1
**Chronic pulmonary Disease**	416.8, 416.9, 490.x-505.x, 506.4, 508.1, 508.8	I27.8, I27.9, J40.x-J47.x, J60.x-J67.x, J68.4, J70.1, J70.3	1
**Rheumatic disease**	446.5, 710.0-710.4, 714.0-714.2, 714.8, 725.x	M05.x, M06.x, M31.5, M32.x-M34.x, M35.1, M35.3, M36.0	1
**Peptic ulcer disease**	531.x-534.x	K25.x-K28.x	1
**Mild liver disease**	070.22, 070.23, 070.32, 070.33,070.44, 070.54, 070.6, 070.9,570.x, 571.x, 573.3, 573.4, 573.8, 573.9, V42.7	B18.x, K70.0-K70.3, K70.9,K71.3-K71.5, K71.7, K73.x, K74.x, K76.0, K76.2-K76.4, K76.8, K76.9,Z94.4	1
**Diabetes without chronic complication**	250.0-250.3, 250.8, 250.9	E10.0, E10.l, E10.6, E10.8, E10.9, E11.0, E11.1, E11.6, E11.8, E11.9, E12.0, E12.1, E12.6, E12.8, E12.9, E13.0, E13.1, E13.6, E13.8, E13.9, E14.0, E14.1, E14.6, E14.8, E14.9	1
**Diabetes with chronic complication**	250.4-250.7	E10.2-E10.5, E10.7, E11.2-E11.5, E11.7, E12.2-E12.5, E12.7, E13.2-E13.5, E13.7, E14.2-E14.5, E14.7	2
**Hemiplegia or paraplegia**	334.1, 342.x, 343.x, 344.0-344.6, 344.9	G04.1, G11.4, G80.1, G80.2, G81.x, G82.x, G83.0-G83.4, G83.9	2
**Renal disease**	403.01, 403.11, 403.91, 404.02, 404.03, 404.12, 404.13, 404.92, 404.93, 582.x, 583.0-583.7, 585.x, 586.x, 588.0, V42.0, V45.1, V56.x	I12.0, I13.1, N03.2-N03.7, N05.2-N05.7, N18.x, N19.x, N25.0, Z49.0-Z49.2, Z94.0, Z99.2	2
**Any malignancy**	140.x-172.x, 174.x-195.8, 200.x-208.x, 238.6	C00.x-C26.x, C30.x-C34.x, C37.x-C41.x, C43.x, C45.x-C58.x, C60.x-C76.x, C81.x-C85.x, C88.x, C90.x-C97.x	3
**Moderate or severe liver disease**	456.0-456.2, 572.2-572.8	I85.0, I85.9, I86.4, I98.2, K70.4, K71.1, K72.1, K72.9, K76.5, K76.6, K76.7	6
**Metastatic solid tumor**	196.x-199.x	C77.x-C80.x	6
**AIDS/HIV**	042.x-044.x	B20.x-B22.x, B24.x	6

Note: The table above outlines the QCI index. To quantify comorbidity, the QCI score is computed by adding the weights that are assigned to the specific diagnoses. A score of 1 is attributed to myocardial infarction, congestive heart failure, peripheral vascular disease, cerebrovascular disease, dementia, chronic pulmonary disease, connective tissue/rheumatologic disease, peptic ulcer disease, mild liver disease, and diabetes without chronic complications. The following diseases are scored as 2: hemiplegia or paraplegia, renal disease, diabetes with complications, and malignancy including leukemia and lymphoma. Moderate or severe liver disease is scored 3. Finally, a score of 6 is assigned to metastatic solid carcinoma and AIDS/HIV. Each diagnosis is only counted once (e.g. if a patient has ICD-9 code 410.xx and 412.xx, they will receive a score of 1 for MI, not 2). The minimum possible score is 0 and the maximum possible score is 33.

ICD-9-CM: International Classification of Disease, Version 9, Clinical Modification; ICD-10-CM: International Classification of Disease, Version 10, Clinical Modification

Appendix 2: Detailed Outputs of Eight Structural Equation Models and Estimated Direct, Indirect and Total Effects

```
----------------------------------------------------------------------------------------------------------------------
                      ------------------------------------------------------------------------

      name:  <unnamed>
       log:  /Users/asim/Dropbox/Integrative Sustainable Solutions/TRUST APP/Cancer and Asthma Application/Health Corp
Data/LateralizationResults20190830.smcl

. ssd init age inpatient_hospitalizations er_visits pcp_officevisits specialist_office_visits other_outpatientservices
pharmacy_fills qci_score mpr pdc rewards engagement_rate

Summary statistics data initialized.  Next use, in any order,

    ssd set observations (required)
        It is best to do this first.

    ssd set means (optional)
        Default setting is 0.

    ssd set variances or ssd set sd (optional)
        Use this only if you have set or will set correlations and, even then, this is optional but highly recommended.
Default setting is 1.

    ssd set covariances or ssd set correlations (required)

.
. ssd set observations 37359
  (value set)

    Status:
                      observations:    set
                             means:  unset
                   variances or sd:  unset
        covariances or correlations:  unset (required to be set)

. ssd set means 49.64 0.04 0.02 2.18 2.61 2.22 9.95 1.47 0.58 0.59 30 234
  (values set)
```

```
    Status:
                       observations:    set
                              means:    set
                    variances or sd:  unset
        covariances or correlations:  unset (required to be set)

.
. ssd set covariances 221.105233\0.089650 0.067846\-0.094017 0.011868 0.040578\2.451662 0.037385 0.033620
6.244202\3.267802 0.033255 0.022245 6.805591 14.548804\ 5.864529 0.113002 0.075032 7.918511 15
> .837080 22.331055\ 14.209445 0.200045 0.084031 4.385411 7.174931 10.065882 54.346633\ 4.632682 0.036237 0.002199
0.1013640 .076749 0.274862 0.771759 1.282943\ 0.803770 0.000065 -0.001100 0.087459 0.
> 131478 0.182628 1.037027 0.018688 0.129239\ 0.761121 -0.000166 -0.000795 0.047573 0.073374 0.105173 0.708629 0.012082
0.062483 0.082140\-0.2 -0.8 -0.6 0.05 0.1 0.15 0.85 0.002 0.7 0.6 225\-0.1 -.6 -
> 0.7 0.08 0.2 0.26 1.04 0.001 0.5 0.46 0.82 32400
  (values set)

    Status:
                       observations:    set
                              means:    set
                    variances or sd:    set
        covariances or correlations:    set
```

. MODEL 1

```
.
. sem (inpatient_hospitalizations -> mpr, ) (er_visits -> mpr, ) (pcp_officevisits -> mpr, ) (specialist_office_visits
-> mpr, ) (other_outpatientservices -> mpr, ) (pharmacy_fills -> mpr, ) (age -> m
> pr, ) (age -> inpatient_hospitalizations, ) (age -> er_visits, ) (age -> pcp_officevisits, ) (age ->
specialist_office_visits, ) (age -> other_outpatientservices, ) (age -> pharmacy_fills, ) (qci_sc
> ore -> mpr, ) (qci_score -> inpatient_hospitalizations, ) (qci_score -> er_visits, ) (qci_score -> pcp_officevisits,
) (qci_score -> specialist_office_visits, ) (qci_score -> other_outpatientservice
> s, ) (qci_score -> pharmacy_fills, ), standardized cov( age*qci_score) nocapslatent

Endogenous variables

Observed:  inpatient_hospitalizations mpr er_visits pcp_officevisits specialist_office_visits other_outpatientservices
pharmacy_fills
```

```
Exogenous variables

Observed:  age qci_score

Fitting target model:

Iteration 0:   log likelihood = -645278.05
Iteration 1:   log likelihood = -645278.05

Structural equation model                       Number of obs     =     37,359
Estimation method  = ml
Log likelihood     = -645278.05

----------------------------------------------------------------------------------------------
                             |                 OIM
                Standardized |      Coef.   Std. Err.      z    P>|z|     [95% Conf. Interval]
-----------------------------+----------------------------------------------------------------
Structural                   |
  inpatient_hospitalizations |
                         age |  -.0115084   .0053399    -2.16   0.031    -.0219744   -.0010424
                   qci_score |   .1259904   .0053009    23.77   0.000     .1156009      .13638
                       _cons |   .0284739   .0180125     1.58   0.114    -.0068299    .0637777
  ---------------------------+----------------------------------------------------------------
  mpr                        |
  inpatient_hospitalizations |  -.0340214   .0049108    -6.93   0.000    -.0436464   -.0243964
                   er_visits |  -.0259384   .0048693    -5.33   0.000    -.0354821   -.0163948
            pcp_officevisits |   .0158356   .0068169     2.32   0.020     .0024747    .0291965
    specialist_office_visits |  -.0107087   .0106578    -1.00   0.315    -.0315976    .0101802
    other_outpatientservices |   -.007845   .0101605    -0.77   0.440    -.0277593    .0120692
             pharmacy_fills |   .3844501   .0045822    83.90   0.000     .3754691     .393431
                         age |    .104232   .0049244    21.17   0.000     .0945804    .1138836
                   qci_score |  -.0139556   .0049505    -2.82   0.005    -.0236585   -.0042527
                       _cons |   .7660481   .0185528    41.29   0.000     .7296853    .8024109
  ---------------------------+----------------------------------------------------------------
  er_visits                  |
                         age |  -.0368249   .0053743    -6.85   0.000    -.0473584   -.0262915
                   qci_score |   .0197669   .0053767     3.68   0.000     .0092288     .030305
                       _cons |   .1965688   .0181193    10.85   0.000     .1610555     .232082
  ---------------------------+----------------------------------------------------------------
  pcp_officevisits           |
```

```
                        age |   .0607252   .0053595    11.33   0.000     .0502207    .0712296
                  qci_score |   .0191099   .0053677     3.56   0.000     .0085893    .0296305
                      _cons |   .6448901   .0184688    34.92   0.000     .6086919    .6810884
  --------------------------+----------------------------------------------------------------
  specialist_office_visits  |
                        age |   .0570456   .0053643    10.63   0.000     .0465318    .0675593
                  qci_score |   .0020736   .0053723     0.39   0.700    -.008456     .0126031
                      _cons |   .4911462   .0183427    26.78   0.000     .4551952    .5270972
  --------------------------+----------------------------------------------------------------
  other_outpatientservices  |
                        age |   .0750104   .0053462    14.03   0.000      .064532    .0854888
                  qci_score |   .0307194   .0053578     5.73   0.000     .0202183    .0412206
                      _cons |   .1795071   .0181625     9.88   0.000     .1439093     .215105
  --------------------------+----------------------------------------------------------------
  pharmacy_fills            |
                        age |   .1127323   .0052952    21.29   0.000      .102354    .1231106
                  qci_score |   .0614172   .0053172    11.55   0.000     .0509956    .0718388
                      _cons |    .893662    .018832    47.45   0.000     .8567521     .930572
----------------------------+----------------------------------------------------------------
                  mean(age)|   3.338398   .0132637   251.69   0.000     3.312401    3.364394
            mean(qci_score)|   1.297835   .0070221   184.82   0.000     1.284072    1.311598
----------------------------+----------------------------------------------------------------
var(e.inpatient_hospitaliz~s)|   .9847916   .0012567                      .9823317    .9872577
                var(e.mpr)|   .8304298   .0036817                      .8232451    .8376772
          var(e.er_visits)|   .9986536   .0003792                      .9979108    .9993971
     var(e.pcp_officevisits)|   .9953089   .0007054                      .9939273    .9966924
var(e.specialist_office_vi~s)|   .9966764   .0005946                      .9955118    .9978424
var(e.other_outpatientserv~s)|   .9921621   .0009089                      .9903823    .9939451
       var(e.pharmacy_fills)|   .9797105    .001444                      .9768844    .9825448
                   var(age)|          1          .                             .           .
             var(qci_score)|          1          .                             .           .
----------------------------+----------------------------------------------------------------
         cov(age,qci_score)|   .2750612   .0047823    57.52   0.000     .2656881    .2844343
---------------------------------------------------------------------------------------------
LR test of model vs. saturated: chi2(15)  =  88816.15, Prob > chi2 = 0.0000
.
. (note: file /Users/asim/Dropbox/Integrative Sustainable Solutions/TRUST APP/Cancer and Asthma Application/Health Corp
Data/SEMS version 2/SEM_M1.stsem not found)
estat gof, stats(all)
```

```
----------------------------------------------------------------------------
Fit statistic        |      Value   Description
---------------------+------------------------------------------------------
Likelihood ratio     |
         chi2_ms(15) |  88816.147   model vs. saturated
            p > chi2 |      0.000
         chi2_bs(35) |  97574.734   baseline vs. saturated
            p > chi2 |      0.000
---------------------+------------------------------------------------------
Population error     |
               RMSEA |      0.398   Root mean squared error of approximation
 90% CI, lower bound |      0.000
         upper bound |          .
              pclose |      0.000   Probability RMSEA <= 0.05
---------------------+------------------------------------------------------
Information criteria |
                 AIC |  1.291e+06   Akaike's information criterion
                 BIC |  1.291e+06   Bayesian information criterion
---------------------+------------------------------------------------------
Baseline comparison  |
                 CFI |      0.090   Comparative fit index
                 TLI |     -1.124   Tucker-Lewis index
---------------------+------------------------------------------------------
Size of residuals    |
                SRMR |      0.211   Standardized root mean squared residual
                  CD |      0.062   Coefficient of determination
----------------------------------------------------------------------------

. estat teffects, standardized


Direct effects
------------------------------------------------------------------------------------------------
                             |                 OIM
                             |      Coef.   Std. Err.      z    P>|z|               Std. Coef.
-----------------------------+------------------------------------------------------------------
Structural                   |
  inpatient_hospitalizations |
                         age |  -.0002016   .0000935    -2.16   0.031               -.0115084
                   qci_score |   .0289732   .0012281    23.59   0.000                .1259904
```

```
  ---------------------------+----------------------------------------------------------------
  mpr                        |
  inpatient_hospitalizations |  -.0470599    .006801    -6.92   0.000                 -.0340214
                   er_visits |  -.0463937   .0087124    -5.33   0.000                 -.0259384
            pcp_officevisits |   .0022833    .000983     2.32   0.020                  .0158356
    specialist_office_visits |  -.0010115   .0010068    -1.00   0.315                 -.0107087
    other_outpatientservices |  -.0005981   .0007748    -0.77   0.440                  -.007845
              pharmacy_fills |   .0187895   .0002437    77.09   0.000                  .3844501
                         age |   .0025256   .0001198    21.08   0.000                   .104232
                   qci_score |  -.0044392   .0015748    -2.82   0.005                 -.0139556
  ---------------------------+----------------------------------------------------------------
  er_visits                  |
                         age |  -.0004989   .0000729    -6.85   0.000                 -.0368249
                   qci_score |   .0035154   .0009564     3.68   0.000                  .0197669
  ---------------------------+----------------------------------------------------------------
  pcp_officevisits           |
                         age |   .0102049   .0009022    11.31   0.000                  .0607252
                   qci_score |   .0421594    .011844     3.56   0.000                  .0191099
  ---------------------------+----------------------------------------------------------------
  specialist_office_visits   |
                         age |   .0146331   .0013781    10.62   0.000                  .0570456
                   qci_score |   .0069828   .0180914     0.39   0.700                  .0020736
  ---------------------------+----------------------------------------------------------------
  other_outpatientservices   |
                         age |   .0238384   .0017035    13.99   0.000                  .0750104
                   qci_score |   .1281635   .0223629     5.73   0.000                  .0307194
  ---------------------------+----------------------------------------------------------------
  pharmacy_fills             |
                         age |   .0558901   .0026407    21.16   0.000                  .1127323
                   qci_score |   .3997355   .0346671    11.53   0.000                  .0614172
-----------------------------------------------------------------------------------------------


Indirect effects
-----------------------------------------------------------------------------------------------
                             |                 OIM
                             |      Coef.   Std. Err.      z    P>|z|               Std. Coef.
-----------------------------+-----------------------------------------------------------------
Structural                   |
  inpatient_hospitalizations |
```

```
                        age |          0  (no path)                                           0
                  qci_score |          0  (no path)                                           0
  --------------------------+----------------------------------------------------------------
  mpr                       |
  inpatient_hospitalizations |          0  (no path)                                           0
                  er_visits |          0  (no path)                                           0
           pcp_officevisits |          0  (no path)                                           0
    specialist_office_visits |          0  (no path)                                           0
   other_outpatientservices |          0  (no path)                                           0
             pharmacy_fills |          0  (no path)                                           0
                        age |    .001077   .0000524    20.57   0.000                   .0444489
                  qci_score |   .0059968    .000691     8.68   0.000                   .0188522
  --------------------------+----------------------------------------------------------------
  er_visits                 |
                        age |          0  (no path)                                           0
                  qci_score |          0  (no path)                                           0
  --------------------------+----------------------------------------------------------------
  pcp_officevisits          |
                        age |          0  (no path)                                           0
                  qci_score |          0  (no path)                                           0
  --------------------------+----------------------------------------------------------------
  specialist_office_visits  |
                        age |          0  (no path)                                           0
                  qci_score |          0  (no path)                                           0
  --------------------------+----------------------------------------------------------------
  other_outpatientservices  |
                        age |          0  (no path)                                           0
                  qci_score |          0  (no path)                                           0
  --------------------------+----------------------------------------------------------------
  pharmacy_fills            |
                        age |          0  (no path)                                           0
                  qci_score |          0  (no path)                                           0
-----------------------------------------------------------------------------------------------


Total effects
-----------------------------------------------------------------------------------------------
                            |                 OIM
                            |      Coef.   Std. Err.      z    P>|z|               Std. Coef.
----------------------------+----------------------------------------------------------------
```

```
Structural                    |
  inpatient_hospitalizations |
                         age |  -.0002016   .0000935    -2.16   0.031                  -.0115084
                   qci_score |   .0289732   .0012281    23.59   0.000                   .1259904
  ---------------------------+----------------------------------------------------------------
  mpr                        |
  inpatient_hospitalizations |  -.0470599    .006801    -6.92   0.000                  -.0340214
                   er_visits |  -.0463937   .0087124    -5.33   0.000                  -.0259384
            pcp_officevisits |   .0022833    .000983     2.32   0.020                   .0158356
    specialist_office_visits |  -.0010115   .0010068    -1.00   0.315                  -.0107087
    other_outpatientservices |  -.0005981   .0007748    -0.77   0.440                   -.007845
              pharmacy_fills |   .0187895   .0002437    77.09   0.000                   .3844501
                         age |   .0036026   .0001289    27.95   0.000                   .1486809
                   qci_score |   .0015576   .0016924     0.92   0.357                   .0048966
  ---------------------------+----------------------------------------------------------------
  er_visits                  |
                         age |  -.0004989   .0000729    -6.85   0.000                  -.0368249
                   qci_score |   .0035154   .0009564     3.68   0.000                   .0197669
  ---------------------------+----------------------------------------------------------------
  pcp_officevisits           |
                         age |   .0102049   .0009022    11.31   0.000                   .0607252
                   qci_score |   .0421594    .011844     3.56   0.000                   .0191099
  ---------------------------+----------------------------------------------------------------
  specialist_office_visits   |
                         age |   .0146331   .0013781    10.62   0.000                   .0570456
                   qci_score |   .0069828   .0180914     0.39   0.700                   .0020736
  ---------------------------+----------------------------------------------------------------
  other_outpatientservices   |
                         age |   .0238384   .0017035    13.99   0.000                   .0750104
                   qci_score |   .1281635   .0223629     5.73   0.000                   .0307194
  ---------------------------+----------------------------------------------------------------
  pharmacy_fills             |
                         age |   .0558901   .0026407    21.16   0.000                   .1127323
                   qci_score |   .3997355   .0346671    11.53   0.000                   .0614172
----------------------------------------------------------------------------------------------

. (note: file /Users/asim/Dropbox/Integrative Sustainable Solutions/TRUST APP/Cancer and Asthma Application/Health Corp
Data/SEMS version 2/SEM_M2.stsem not found)
```

MODEL 2

```
sem (inpatient_hospitalizations -> pdc, ) (er_visits -> pdc, ) (pcp_officevisits -> pdc, ) (specialist_office_visits ->
pdc, ) (other_outpatientservices -> pdc, ) (pharmacy_fills -> pdc, ) (age -> pdc
> , ) (age -> inpatient_hospitalizations, ) (age -> er_visits, ) (age -> pcp_officevisits, ) (age ->
specialist_office_visits, ) (age -> other_outpatientservices, ) (age -> pharmacy_fills, ) (qci_scor
> e -> pdc, ) (qci_score -> inpatient_hospitalizations, ) (qci_score -> er_visits, ) (qci_score -> pcp_officevisits, )
(qci_score -> specialist_office_visits, ) (qci_score -> other_outpatientservices,
>  ) (qci_score -> pharmacy_fills, ), standardized cov( age*qci_score) nocapslatent

Endogenous variables

Observed:  inpatient_hospitalizations pdc er_visits pcp_officevisits specialist_office_visits other_outpatientservices
pharmacy_fills

Exogenous variables

Observed:  age qci_score

Fitting target model:

Iteration 0:   log likelihood = -637507.53
Iteration 1:   log likelihood = -637507.53

Structural equation model                       Number of obs     =     37,359
Estimation method  = ml
Log likelihood     = -637507.53

--------------------------------------------------------------------------------------------
                             |                 OIM
                Standardized |      Coef.   Std. Err.      z    P>|z|     [95% Conf. Interval]
-----------------------------+--------------------------------------------------------------
Structural                   |
  inpatient_hospitalizations |
                         age |  -.0115084   .0053399    -2.16   0.031    -.0219744   -.0010424
                   qci_score |   .1259904   .0053009    23.77   0.000     .1156009      .13638
                       _cons |   .0284739   .0180125     1.58   0.114    -.0068299    .0637777
  ---------------------------+--------------------------------------------------------------
```

pdc						
inpatient_hospitalizations	-.0303064	.0049997	-6.06	0.000	-.0401055	-.0205072
er_visits	-.0191494	.004957	-3.86	0.000	-.0288648	-.0094339
pcp_officevisits	-.0006992	.0069386	-0.10	0.920	-.0142986	.0129002
specialist_office_visits	-.0093955	.0108487	-0.87	0.386	-.0306586	.0118676
other_outpatientservices	-.0154057	.0103394	-1.49	0.136	-.0356706	.0048592
pharmacy_fills	.3293921	.0047729	69.01	0.000	.3200373	.3387468
age	.1451071	.0049904	29.08	0.000	.135326	.1548882
qci_score	-.0283679	.0050377	-5.63	0.000	-.0382417	-.0184941
_cons	1.180709	.0196405	60.12	0.000	1.142214	1.219204
er_visits						
age	-.0368249	.0053743	-6.85	0.000	-.0473584	-.0262915
qci_score	.0197669	.0053767	3.68	0.000	.0092288	.030305
_cons	.1965688	.0181193	10.85	0.000	.1610555	.232082
pcp_officevisits						
age	.0607252	.0053595	11.33	0.000	.0502207	.0712296
qci_score	.0191099	.0053677	3.56	0.000	.0085893	.0296305
_cons	.6448901	.0184688	34.92	0.000	.6086919	.6810884
specialist_office_visits						
age	.0570456	.0053643	10.63	0.000	.0465318	.0675593
qci_score	.0020736	.0053723	0.39	0.700	-.008456	.0126031
_cons	.4911462	.0183427	26.78	0.000	.4551952	.5270972
other_outpatientservices						
age	.0750104	.0053462	14.03	0.000	.064532	.0854888
qci_score	.0307194	.0053578	5.73	0.000	.0202183	.0412206
_cons	.1795071	.0181625	9.88	0.000	.1439093	.215105
pharmacy_fills						
age	.1127323	.0052952	21.29	0.000	.102354	.1231106
qci_score	.0614172	.0053172	11.55	0.000	.0509956	.0718388
_cons	.893662	.018832	47.45	0.000	.8567521	.930572
mean(age)	3.338398	.0132637	251.69	0.000	3.312401	3.364394
mean(qci_score)	1.297835	.0070221	184.82	0.000	1.284072	1.311598
var(e.inpatient_hospitaliz~s)	.9847916	.0012567			.9823317	.9872577

```
                var(e.pdc)|   .8602612   .0034683                      .8534903    .8670858
          var(e.er_visits)|   .9986536   .0003792                      .9979108    .9993971
   var(e.pcp_officevisits)|   .9953089   .0007054                      .9939273    .9966924
var(e.specialist_office_vi~s)|   .9966764   .0005946                      .9955118    .9978424
var(e.other_outpatientserv~s)|   .9921621   .0009089                      .9903823    .9939451
     var(e.pharmacy_fills)|   .9797105    .001444                      .9768844    .9825448
                  var(age)|          1          .                             .           .
            var(qci_score)|          1          .                             .           .
--------------------------+----------------------------------------------------------------
      cov(age,qci_score)|   .2750612   .0047823    57.52   0.000     .2656881    .2844343
-------------------------------------------------------------------------------------------
LR test of model vs. saturated: chi2(15)  =  88816.15, Prob > chi2 = 0.0000
.
. estat gof, stats(all)

----------------------------------------------------------------------------
Fit statistic        |      Value   Description
---------------------+------------------------------------------------------
Likelihood ratio     |
         chi2_ms(15) |  88816.147   model vs. saturated
            p > chi2 |      0.000
         chi2_bs(35) |  96183.252   baseline vs. saturated
            p > chi2 |      0.000
---------------------+------------------------------------------------------
Population error     |
               RMSEA |      0.398   Root mean squared error of approximation
 90% CI, lower bound |      0.000
         upper bound |          .
              pclose |      0.000   Probability RMSEA <= 0.05
---------------------+------------------------------------------------------
Information criteria |
                 AIC |  1.275e+06   Akaike's information criterion
                 BIC |  1.275e+06   Bayesian information criterion
---------------------+------------------------------------------------------
Baseline comparison  |
                 CFI |      0.076   Comparative fit index
                 TLI |     -1.155   Tucker-Lewis index
---------------------+------------------------------------------------------
Size of residuals    |
                SRMR |      0.211   Standardized root mean squared residual
```

```
                  CD |      0.072   Coefficient of determination
----------------------------------------------------------------------------

. estat teffects, standardized


Direct effects
-----------------------------------------------------------------------------------------------
                             |                OIM
                             |      Coef.   Std. Err.      z    P>|z|              Std. Coef.
-----------------------------+-----------------------------------------------------------------
Structural                   |
  inpatient_hospitalizations |
                         age |  -.0002016   .0000935    -2.16   0.031                -.0115084
                   qci_score |   .0289732   .0012281    23.59   0.000                 .1259904
  ---------------------------+-----------------------------------------------------------------
  pdc                        |
  inpatient_hospitalizations |  -.0334531   .0055239    -6.06   0.000                -.0303064
                   er_visits |  -.0273322   .0070763    -3.86   0.000                -.0191494
           pcp_officevisits |  -.0000805   .0007984    -0.10   0.920                -.0006992
    specialist_office_visits |  -.0007082   .0008178    -0.87   0.386                -.0093955
    other_outpatientservices |  -.0009373   .0006293    -1.49   0.136                -.0154057
              pharmacy_fills |   .0128467    .000198    64.90   0.000                 .3293921
                         age |   .0028058   .0000973    28.83   0.000                 .1451071
                   qci_score |  -.0072009   .0012791    -5.63   0.000                -.0283679
  ---------------------------+-----------------------------------------------------------------
  er_visits                  |
                         age |  -.0004989   .0000729    -6.85   0.000                -.0368249
                   qci_score |   .0035154   .0009564     3.68   0.000                 .0197669
  ---------------------------+-----------------------------------------------------------------
  pcp_officevisits           |
                         age |   .0102049   .0009022    11.31   0.000                 .0607252
                   qci_score |   .0421594    .011844     3.56   0.000                 .0191099
  ---------------------------+-----------------------------------------------------------------
  specialist_office_visits   |
                         age |   .0146331   .0013781    10.62   0.000                 .0570456
                   qci_score |   .0069828   .0180914     0.39   0.700                 .0020736
  ---------------------------+-----------------------------------------------------------------
  other_outpatientservices   |
                         age |   .0238384   .0017035    13.99   0.000                 .0750104
```

```
                qci_score |   .1281635   .0223629     5.73   0.000                 .0307194
  --------------------------+----------------------------------------------------------------
  pharmacy_fills            |
                      age |   .0558901   .0026407    21.16   0.000                 .1127323
                qci_score |   .3997355   .0346671    11.53   0.000                 .0614172
------------------------------------------------------------------------------------------------


Indirect effects
------------------------------------------------------------------------------------------------
                            |                 OIM
                            |      Coef.   Std. Err.      z    P>|z|               Std. Coef.
----------------------------+-------------------------------------------------------------------
Structural                  |
  inpatient_hospitalizations |
                      age |          0  (no path)                                          0
                qci_score |          0  (no path)                                          0
  --------------------------+----------------------------------------------------------------
  pdc                       |
  inpatient_hospitalizations |         0  (no path)                                          0
                er_visits |          0  (no path)                                          0
          pcp_officevisits |          0  (no path)                                          0
    specialist_office_visits |        0  (no path)                                          0
    other_outpatientservices |        0  (no path)                                          0
            pharmacy_fills |          0  (no path)                                          0
                      age |   .0007049   .0000364    19.35   0.000                  .036453
                qci_score |   .0039415   .0004818     8.18   0.000                 .0155274
  --------------------------+----------------------------------------------------------------
  er_visits                 |
                      age |          0  (no path)                                          0
                qci_score |          0  (no path)                                          0
  --------------------------+----------------------------------------------------------------
  pcp_officevisits          |
                      age |          0  (no path)                                          0
                qci_score |          0  (no path)                                          0
  --------------------------+----------------------------------------------------------------
  specialist_office_visits  |
                      age |          0  (no path)                                          0
                qci_score |          0  (no path)                                          0
  --------------------------+----------------------------------------------------------------
```

```
  other_outpatientservices   |
                         age |          0  (no path)                                                 0
                   qci_score |          0  (no path)                                                 0
  ---------------------------+----------------------------------------------------------------------
  pharmacy_fills             |
                         age |          0  (no path)                                                 0
                   qci_score |          0  (no path)                                                 0
----------------------------------------------------------------------------------------------------


Total effects
----------------------------------------------------------------------------------------------------
                             |                 OIM
                             |      Coef.   Std. Err.      z    P>|z|                  Std. Coef.
-----------------------------+----------------------------------------------------------------------
Structural                   |
  inpatient_hospitalizations |
                         age |  -.0002016   .0000935    -2.16   0.031                   -.0115084
                   qci_score |   .0289732   .0012281    23.59   0.000                    .1259904
  ---------------------------+----------------------------------------------------------------------
  pdc                        |
  inpatient_hospitalizations |  -.0334531   .0055239    -6.06   0.000                   -.0303064
                   er_visits |  -.0273322   .0070763    -3.86   0.000                   -.0191494
            pcp_officevisits |  -.0000805   .0007984    -0.10   0.920                   -.0006992
    specialist_office_visits |  -.0007082   .0008178    -0.87   0.386                   -.0093955
    other_outpatientservices |  -.0009373   .0006293    -1.49   0.136                   -.0154057
              pharmacy_fills |   .0128467    .000198    64.90   0.000                    .3293921
                         age |   .0035106   .0001024    34.29   0.000                    .1815601
                   qci_score |  -.0032594   .0013441    -2.43   0.015                   -.0128405
  ---------------------------+----------------------------------------------------------------------
  er_visits                  |
                         age |  -.0004989   .0000729    -6.85   0.000                   -.0368249
                   qci_score |   .0035154   .0009564     3.68   0.000                    .0197669
  ---------------------------+----------------------------------------------------------------------
  pcp_officevisits           |
                         age |   .0102049   .0009022    11.31   0.000                    .0607252
                   qci_score |   .0421594    .011844     3.56   0.000                    .0191099
  ---------------------------+----------------------------------------------------------------------
  specialist_office_visits   |
                         age |   .0146331   .0013781    10.62   0.000                    .0570456
```

```
                 qci_score |   .0069828   .0180914     0.39   0.700                 .0020736
  ---------------------------+----------------------------------------------------------------
  other_outpatientservices   |
                       age |   .0238384   .0017035    13.99   0.000                 .0750104
                 qci_score |   .1281635   .0223629     5.73   0.000                 .0307194
  ---------------------------+----------------------------------------------------------------
  pharmacy_fills             |
                       age |   .0558901   .0026407    21.16   0.000                 .1127323
                 qci_score |   .3997355   .0346671    11.53   0.000                 .0614172
--------------------------------------------------------------------------------------------------

. (note: file /Users/asim/Dropbox/Integrative Sustainable Solutions/TRUST APP/Cancer and Asthma Application/Health Corp
Data/SEMS version 2/SEM_M3.stsem not found)
```

MODEL 3

```
sem (inpatient_hospitalizations -> mpr, ) (er_visits -> mpr, ) (pcp_officevisits -> mpr, ) (specialist_office_visits ->
mpr, ) (other_outpatientservices -> mpr, ) (pharmacy_fills -> mpr, ) (age -> mpr
> , ) (age -> inpatient_hospitalizations, ) (age -> er_visits, ) (age -> pcp_officevisits, ) (age ->
specialist_office_visits, ) (age -> other_outpatientservices, ) (age -> pharmacy_fills, ) (qci_scor
> e -> mpr, ) (qci_score -> inpatient_hospitalizations, ) (qci_score -> er_visits, ) (qci_score -> pcp_officevisits, )
(qci_score -> specialist_office_visits, ) (qci_score -> other_outpatientservices,
>  ) (qci_score -> pharmacy_fills, ) (rewards -> mpr, ) (rewards -> inpatient_hospitalizations, ) (rewards ->
er_visits, ) (rewards -> pcp_officevisits, ) (rewards -> specialist_office_visits, ) (rewa
> rds -> other_outpatientservices, ) (rewards -> pharmacy_fills, ), standardized cov( age*qci_score age*rewards
qci_score*rewards) nocapslatent

Endogenous variables

Observed:  inpatient_hospitalizations mpr er_visits pcp_officevisits specialist_office_visits other_outpatientservices
pharmacy_fills

Exogenous variables

Observed:  age qci_score rewards
```

```
Fitting target model:

Iteration 0:   log likelihood =  -797570.7
Iteration 1:   log likelihood =  -797570.7

Structural equation model                      Number of obs     =     37,359
Estimation method  = ml
Log likelihood     =  -797570.7

-----------------------------------------------------------------------------------------------
                             |                 OIM
                Standardized |      Coef.   Std. Err.      z    P>|z|     [95% Conf. Interval]
-----------------------------+-----------------------------------------------------------------
Structural                   |
  inpatient_hospitalizations |
                         age |  -.0117142    .005225    -2.24   0.025     -.021955   -.0014735
                   qci_score |   .1260711   .0051885    24.30   0.000     .1159019    .1362404
                     rewards |  -.2047813    .004919   -41.63   0.000    -.2144224   -.1951403
                       _cons |   .4386244   .0201977    21.72   0.000     .3990376    .4782112
  ---------------------------+-----------------------------------------------------------------
  mpr                        |
  inpatient_hospitalizations |  -.0120911   .0049527    -2.44   0.015    -.0217982    -.002384
                   er_visits |  -.0055437   .0048992    -1.13   0.258    -.0151459    .0040585
            pcp_officevisits |   .0136725   .0067649     2.02   0.043     .0004135    .0269314
    specialist_office_visits |  -.0014893    .010582    -0.14   0.888    -.0222297     .019251
    other_outpatientservices |   -.017402   .0100837    -1.73   0.084    -.0371656    .0023617
              pharmacy_fills |   .3812777   .0045604    83.61   0.000     .3723395     .390216
                         age |   .1061491   .0048856    21.73   0.000     .0965735    .1157247
                   qci_score |   -.016656   .0049132    -3.39   0.001    -.0262857   -.0070264
                     rewards |   .1232295   .0048269    25.53   0.000     .1137689    .1326902
                       _cons |   .5168378   .0206838    24.99   0.000     .4762983    .5573772
  ---------------------------+-----------------------------------------------------------------
  er_visits                  |
                         age |  -.0370246   .0052672    -7.03   0.000    -.0473481   -.0267011
                   qci_score |   .0198452   .0052695     3.77   0.000     .0095172    .0301731
                     rewards |   -.198606   .0049663   -39.99   0.000    -.2083398   -.1888722
                       _cons |   .5943509     .02021    29.41   0.000     .5547401    .6339617
  ---------------------------+-----------------------------------------------------------------
  pcp_officevisits           |
                         age |   .0607266   .0053595    11.33   0.000     .0502221     .071231
```

qci_score	.0191094	.0053677	3.56	0.000	.0085888	.02963
rewards	.0013862	.0051616	0.27	0.788	-.0087303	.0115026
_cons	.6421138	.0211656	30.34	0.000	.60063	.6835977
specialist_office_visits						
age	.0570474	.0053642	10.63	0.000	.0465337	.0675611
qci_score	.0020729	.0053723	0.39	0.700	-.0084567	.0126024
rewards	.0017987	.0051651	0.35	0.728	-.0083247	.0119221
_cons	.4875436	.021059	23.15	0.000	.4462687	.5288185
other_outpatientservices						
age	.0750126	.0053462	14.03	0.000	.0645342	.0854909
qci_score	.0307186	.0053578	5.73	0.000	.0202174	.0412197
rewards	.0021798	.0051534	0.42	0.672	-.0079206	.0122802
_cons	.1751413	.0208893	8.38	0.000	.134199	.2160836
pharmacy_fills						
age	.1127401	.005295	21.29	0.000	.1023621	.1231181
qci_score	.0614141	.0053171	11.55	0.000	.0509929	.0718354
rewards	.0077806	.0051207	1.52	0.129	-.0022557	.0178169
_cons	.8780785	.0214427	40.95	0.000	.8360516	.9201054
mean(age)	3.338398	.0132637	251.69	0.000	3.312401	3.364394
mean(qci_score)	1.297835	.0070221	184.82	0.000	1.284072	1.311598
mean(rewards)	2.000027	.0089612	223.19	0.000	1.982463	2.01759
var(e.inpatient_hospitaliz~s)	.9428563	.0023322			.9382963	.9474384
var(e.mpr)	.8175303	.0037562			.8102013	.8249257
var(e.er_visits)	.9592093	.0020046			.9552884	.9631463
var(e.pcp_officevisits)	.995307	.0007055			.9939251	.9966907
var(e.specialist_office_vi~s)	.9966732	.0005948			.995508	.9978397
var(e.other_outpatientserv~s)	.9921574	.0009092			.990377	.9939409
var(e.pharmacy_fills)	.9796499	.0014461			.9768198	.9824883
var(age)	1	.			.	.
var(qci_score)	1	.			.	.
var(rewards)	1	.			.	.
cov(age,qci_score)	.2750612	.0047823	57.52	0.000	.2656881	.2844343
cov(age,rewards)	-.0008967	.0051737	-0.17	0.862	-.011037	.0092436
cov(qci_score,rewards)	.0001177	.0051737	0.02	0.982	-.0100226	.010258

```
---------------------------------------------------------------------------------------------
LR test of model vs. saturated: chi2(15)  =  88379.85, Prob > chi2 = 0.0000
.
. (note: file /Users/asim/Dropbox/Integrative Sustainable Solutions/TRUST APP/Cancer and Asthma Application/Health Corp
Data/SEMS version 2/SEM_M4.stsem not found)
estat gof, stats(all)

----------------------------------------------------------------------------
Fit statistic        |      Value   Description
---------------------+------------------------------------------------------
Likelihood ratio     |
         chi2_ms(15) |  88379.852   model vs. saturated
            p > chi2 |      0.000
         chi2_bs(42) | 100912.442   baseline vs. saturated
            p > chi2 |      0.000
---------------------+------------------------------------------------------
Population error     |
               RMSEA |      0.397   Root mean squared error of approximation
 90% CI, lower bound |      0.000
         upper bound |          .
              pclose |      0.000   Probability RMSEA <= 0.05
---------------------+------------------------------------------------------
Information criteria |
                 AIC |  1.595e+06   Akaike's information criterion
                 BIC |  1.596e+06   Bayesian information criterion
---------------------+------------------------------------------------------
Baseline comparison  |
                 CFI |      0.124   Comparative fit index
                 TLI |     -1.453   Tucker-Lewis index
---------------------+------------------------------------------------------
Size of residuals    |
                SRMR |      0.191   Standardized root mean squared residual
                  CD |      0.151   Coefficient of determination
----------------------------------------------------------------------------

. estat teffects, standardized


Direct effects
---------------------------------------------------------------------------------------------
```

```
                             |                   OIM
                             |      Coef.   Std. Err.      z    P>|z|               Std. Coef.
-----------------------------+----------------------------------------------------------------
Structural                   |
  inpatient_hospitalizations |
                         age |  -.0002052   .0000915    -2.24   0.025                -.0117142
                   qci_score |   .0289917   .0012016    24.13   0.000                 .1260711
                     rewards |   -.003556   .0000872   -40.76   0.000                -.2047813
  ---------------------------+----------------------------------------------------------------
  mpr                        |
  inpatient_hospitalizations |  -.0167126    .006848    -2.44   0.015                -.0120911
                   er_visits |  -.0099082   .0087566    -1.13   0.258                -.0055437
          pcp_officevisits |   .0019699   .0009746     2.02   0.043                 .0136725
    specialist_office_visits |  -.0001406   .0009988    -0.14   0.888                -.0014893
    other_outpatientservices |  -.0013258   .0007687    -1.72   0.085                 -.017402
             pharmacy_fills |   .0186207   .0002417    77.03   0.000                 .3812777
                         age |   .0025701   .0001188    21.63   0.000                 .1061491
                   qci_score |  -.0052943   .0015617    -3.39   0.001                 -.016656
                     rewards |   .0029578   .0001164    25.41   0.000                 .1232295
  ---------------------------+----------------------------------------------------------------
  er_visits                  |
                         age |  -.0005016   .0000714    -7.03   0.000                -.0370246
                   qci_score |   .0035294   .0009373     3.77   0.000                 .0198452
                     rewards |  -.0026671    .000068   -39.20   0.000                 -.198606
  ---------------------------+----------------------------------------------------------------
  pcp_officevisits           |
                         age |   .0102051   .0009022    11.31   0.000                 .0607266
                   qci_score |   .0421582    .011844     3.56   0.000                 .0191094
                     rewards |   .0002309   .0008599     0.27   0.788                 .0013862
  ---------------------------+----------------------------------------------------------------
  specialist_office_visits   |
                         age |   .0146336   .0013781    10.62   0.000                 .0570474
                   qci_score |   .0069804   .0180914     0.39   0.700                 .0020729
                     rewards |   .0004574   .0013134     0.35   0.728                 .0017987
  ---------------------------+----------------------------------------------------------------
  other_outpatientservices   |
                         age |   .0238391   .0017035    13.99   0.000                 .0750126
                   qci_score |   .1281599   .0223629     5.73   0.000                 .0307186
                     rewards |   .0006867   .0016235     0.42   0.672                 .0021798
  ---------------------------+----------------------------------------------------------------
```

```
  pharmacy_fills             |
                         age |    .055894   .0026406    21.17   0.000                 .1127401
                   qci_score |   .3997155    .034666    11.53   0.000                 .0614141
                     rewards |   .0038239   .0025167     1.52   0.129                 .0077806
----------------------------------------------------------------------------------------------


Indirect effects
----------------------------------------------------------------------------------------------
                             |                 OIM
                             |      Coef.   Std. Err.      z    P>|z|               Std. Coef.
-----------------------------+----------------------------------------------------------------
Structural                   |
  inpatient_hospitalizations |
                         age |          0  (no path)                                         0
                   qci_score |          0  (no path)                                         0
                     rewards |          0  (no path)                                         0
  ---------------------------+----------------------------------------------------------------
  mpr                        |
  inpatient_hospitalizations |          0  (no path)                                         0
                   er_visits |          0  (no path)                                         0
          pcp_officevisits |          0  (no path)                                         0
    specialist_office_visits |          0  (no path)                                         0
   other_outpatientservices |          0  (no path)                                         0
              pharmacy_fills |          0  (no path)                                         0
                         age |   .0010356   .0000517    20.05   0.000                 .0427721
                   qci_score |   .0068356   .0006824    10.02   0.000                 .0215051
                     rewards |   .0001565   .0000561     2.79   0.005                 .0065219
  ---------------------------+----------------------------------------------------------------
  er_visits                  |
                         age |          0  (no path)                                         0
                   qci_score |          0  (no path)                                         0
                     rewards |          0  (no path)                                         0
  ---------------------------+----------------------------------------------------------------
  pcp_officevisits           |
                         age |          0  (no path)                                         0
                   qci_score |          0  (no path)                                         0
                     rewards |          0  (no path)                                         0
  ---------------------------+----------------------------------------------------------------
  specialist_office_visits   |
```

```
                    age |          0  (no path)                                         0
              qci_score |          0  (no path)                                         0
                rewards |          0  (no path)                                         0
  ----------------------------+----------------------------------------------------------------
  other_outpatientservices   |
                    age |          0  (no path)                                         0
              qci_score |          0  (no path)                                         0
                rewards |          0  (no path)                                         0
  ----------------------------+----------------------------------------------------------------
  pharmacy_fills             |
                    age |          0  (no path)                                         0
              qci_score |          0  (no path)                                         0
                rewards |          0  (no path)                                         0
----------------------------------------------------------------------------------------------


Total effects
----------------------------------------------------------------------------------------------
                             |                 OIM
                             |      Coef.   Std. Err.      z    P>|z|              Std. Coef.
-----------------------------+----------------------------------------------------------------
Structural                   |
  inpatient_hospitalizations |
                         age |  -.0002052   .0000915    -2.24   0.025               -.0117142
                   qci_score |   .0289917   .0012016    24.13   0.000                .1260711
                     rewards |   -.003556   .0000872   -40.76   0.000               -.2047813
  ---------------------------+----------------------------------------------------------------
  mpr                        |
  inpatient_hospitalizations |  -.0167126    .006848    -2.44   0.015               -.0120911
                   er_visits |  -.0099082   .0087566    -1.13   0.258               -.0055437
             pcp_officevisits |   .0019699   .0009746     2.02   0.043                .0136725
    specialist_office_visits |  -.0001406   .0009988    -0.14   0.888               -.0014893
    other_outpatientservices |  -.0013258   .0007687    -1.72   0.085                -.017402
              pharmacy_fills |   .0186207   .0002417    77.03   0.000                .3812777
                         age |   .0036058   .0001277    28.24   0.000                .1489212
                   qci_score |   .0015413   .0016765     0.92   0.358                .0048491
                     rewards |   .0031143   .0001217    25.59   0.000                .1297515
  ---------------------------+----------------------------------------------------------------
  er_visits                  |
                         age |  -.0005016   .0000714    -7.03   0.000               -.0370246
```

```
                 qci_score |   .0035294   .0009373     3.77   0.000                 .0198452
                   rewards |  -.0026671    .000068   -39.20   0.000                 -.198606
  -------------------------+----------------------------------------------------------------
  pcp_officevisits         |
                       age |   .0102051   .0009022    11.31   0.000                 .0607266
                 qci_score |   .0421582    .011844     3.56   0.000                 .0191094
                   rewards |   .0002309   .0008599     0.27   0.788                 .0013862
  -------------------------+----------------------------------------------------------------
  specialist_office_visits  |
                       age |   .0146336   .0013781    10.62   0.000                 .0570474
                 qci_score |   .0069804   .0180914     0.39   0.700                 .0020729
                   rewards |   .0004574   .0013134     0.35   0.728                 .0017987
  -------------------------+----------------------------------------------------------------
  other_outpatientservices  |
                       age |   .0238391   .0017035    13.99   0.000                 .0750126
                 qci_score |   .1281599   .0223629     5.73   0.000                 .0307186
                   rewards |   .0006867   .0016235     0.42   0.672                 .0021798
  -------------------------+----------------------------------------------------------------
  pharmacy_fills           |
                       age |    .055894   .0026406    21.17   0.000                 .1127401
                 qci_score |   .3997155    .034666    11.53   0.000                 .0614141
                   rewards |   .0038239   .0025167     1.52   0.129                 .0077806
--------------------------------------------------------------------------------------------
```

MODEL 4

```
. sem (inpatient_hospitalizations -> pdc, ) (er_visits -> pdc, ) (pcp_officevisits -> pdc, ) (specialist_office_visits
-> pdc, ) (other_outpatientservices -> pdc, ) (pharmacy_fills -> pdc, ) (age -> p
> dc, ) (age -> inpatient_hospitalizations, ) (age -> er_visits, ) (age -> pcp_officevisits, ) (age ->
specialist_office_visits, ) (age -> other_outpatientservices, ) (age -> pharmacy_fills, ) (qci_sc
> ore -> pdc, ) (qci_score -> inpatient_hospitalizations, ) (qci_score -> er_visits, ) (qci_score -> pcp_officevisits,
) (qci_score -> specialist_office_visits, ) (qci_score -> other_outpatientservice
> s, ) (qci_score -> pharmacy_fills, ) (rewards -> pdc, ) (rewards -> inpatient_hospitalizations, ) (rewards ->
er_visits, ) (rewards -> pcp_officevisits, ) (rewards -> specialist_office_visits, ) (re
> wards -> other_outpatientservices, ) (rewards -> pharmacy_fills, ), standardized cov( age*qci_score age*rewards
qci_score*rewards) nocapslatent

Endogenous variables
```

```
Observed:  inpatient_hospitalizations pdc er_visits pcp_officevisits specialist_office_visits other_outpatientservices
pharmacy_fills

Exogenous variables

Observed:  age qci_score rewards

Fitting target model:

Iteration 0:   log likelihood = -789741.35
Iteration 1:   log likelihood = -789741.35  (backed up)

Structural equation model                       Number of obs     =     37,359
Estimation method  = ml
Log likelihood     = -789741.35

----------------------------------------------------------------------------------------------
                             |                 OIM
                Standardized |      Coef.   Std. Err.      z    P>|z|     [95% Conf. Interval]
-----------------------------+----------------------------------------------------------------
Structural                   |
  inpatient_hospitalizations |
                         age |  -.0117142    .005225    -2.24   0.025     -.021955   -.0014735
                   qci_score |   .1260711   .0051885    24.30   0.000     .1159019    .1362404
                     rewards |  -.2047813    .004919   -41.63   0.000    -.2144224   -.1951403
                       _cons |   .4386244   .0201977    21.72   0.000     .3990376    .4782112
  ---------------------------+----------------------------------------------------------------
  pdc                        |
  inpatient_hospitalizations |  -.0060334   .0050333    -1.20   0.231    -.0158985    .0038316
                   er_visits |   .0034248   .0049781     0.69   0.491    -.0063321    .0131816
            pcp_officevisits |  -.0031058    .006873    -0.45   0.651    -.0165767    .0103651
    specialist_office_visits |   .0008078   .0107517     0.08   0.940    -.0202651    .0218807
    other_outpatientservices |  -.0259822   .0102411    -2.54   0.011    -.0460544     -.00591
              pharmacy_fills |   .3257658   .0047412    68.71   0.000     .3164731    .3350584
                         age |   .1472301   .0049425    29.79   0.000      .137543    .1569172
                   qci_score |  -.0313613   .0049912    -6.28   0.000    -.0411438   -.0215787
                     rewards |   .1363392   .0048957    27.85   0.000     .1267438    .1459347
                       _cons |   .9050695   .0217151    41.68   0.000     .8625087    .9476303
  ---------------------------+----------------------------------------------------------------
```

```
  er_visits                  |
                         age |  -.0370246   .0052672    -7.03   0.000    -.0473481   -.0267011
                   qci_score |   .0198452   .0052695     3.77   0.000     .0095172    .0301731
                     rewards |   -.198606   .0049663   -39.99   0.000    -.2083398   -.1888722
                       _cons |   .5943509     .02021    29.41   0.000     .5547401    .6339617
  ---------------------------+----------------------------------------------------------------
  pcp_officevisits           |
                         age |   .0607266   .0053595    11.33   0.000     .0502221     .071231
                   qci_score |   .0191094   .0053677     3.56   0.000     .0085888      .02963
                     rewards |   .0013862   .0051616     0.27   0.788    -.0087303    .0115026
                       _cons |   .6421138   .0211656    30.34   0.000       .60063    .6835977
  ---------------------------+----------------------------------------------------------------
  specialist_office_visits   |
                         age |   .0570474   .0053642    10.63   0.000     .0465337    .0675611
                   qci_score |   .0020729   .0053723     0.39   0.700    -.0084567    .0126024
                     rewards |   .0017987   .0051651     0.35   0.728    -.0083247    .0119221
                       _cons |   .4875436    .021059    23.15   0.000     .4462687    .5288185
  ---------------------------+----------------------------------------------------------------
  other_outpatientservices   |
                         age |   .0750126   .0053462    14.03   0.000     .0645342    .0854909
                   qci_score |   .0307186   .0053578     5.73   0.000     .0202174    .0412197
                     rewards |   .0021798   .0051534     0.42   0.672    -.0079206    .0122802
                       _cons |   .1751413   .0208893     8.38   0.000      .134199    .2160836
  ---------------------------+----------------------------------------------------------------
  pharmacy_fills             |
                         age |   .1127401    .005295    21.29   0.000     .1023621    .1231181
                   qci_score |   .0614141   .0053171    11.55   0.000     .0509929    .0718354
                     rewards |   .0077806   .0051207     1.52   0.129    -.0022557    .0178169
                       _cons |   .8780785   .0214427    40.95   0.000     .8360516    .9201054
-----------------------------+----------------------------------------------------------------
                   mean(age)|   3.338398   .0132637   251.69   0.000     3.312401    3.364394
             mean(qci_score)|   1.297835   .0070221   184.82   0.000     1.284072    1.311598
               mean(rewards)|   2.000027   .0089612   223.19   0.000     1.982463     2.01759
-----------------------------+----------------------------------------------------------------
var(e.inpatient_hospitaliz~s)|   .9428563   .0023322                      .9382963    .9474384
                  var(e.pdc)|    .844002   .0036047                      .8369665    .8510967
            var(e.er_visits)|   .9592093   .0020046                      .9552884    .9631463
     var(e.pcp_officevisits)|    .995307   .0007055                      .9939251    .9966907
var(e.specialist_office_vi~s)|   .9966732   .0005948                       .995508    .9978397
var(e.other_outpatientserv~s)|   .9921574   .0009092                       .990377    .9939409
```

```
       var(e.pharmacy_fills)|   .9796499   .0014461                      .9768198    .9824883
                    var(age)|          1          .                             .           .
                var(qci_score)|        1          .                             .           .
                 var(rewards)|         1          .                             .           .
-----------------------------+----------------------------------------------------------------
          cov(age,qci_score)|   .2750612   .0047823    57.52   0.000     .2656881    .2844343
            cov(age,rewards)|  -.0008967   .0051737    -0.17   0.862    -.011037    .0092436
      cov(qci_score,rewards)|   .0001177   .0051737     0.02   0.982   -.0100226     .010258
----------------------------------------------------------------------------------------------
LR test of model vs. saturated: chi2(15)  =  88379.85, Prob > chi2 = 0.0000
.
. estat gof, stats(all)

----------------------------------------------------------------------------
Fit statistic        |      Value   Description
---------------------+------------------------------------------------------
Likelihood ratio     |
         chi2_ms(15) |  88379.852   model vs. saturated
            p > chi2 |      0.000
         chi2_bs(42) |  99638.621   baseline vs. saturated
            p > chi2 |      0.000
---------------------+------------------------------------------------------
Population error     |
               RMSEA |      0.397   Root mean squared error of approximation
 90% CI, lower bound |      0.000
         upper bound |          .
              pclose |      0.000   Probability RMSEA <= 0.05
---------------------+------------------------------------------------------
Information criteria |
                 AIC |  1.580e+06   Akaike's information criterion
                 BIC |  1.580e+06   Bayesian information criterion
---------------------+------------------------------------------------------
Baseline comparison  |
                 CFI |      0.113   Comparative fit index
                 TLI |     -1.484   Tucker-Lewis index
---------------------+------------------------------------------------------
Size of residuals    |
                SRMR |      0.190   Standardized root mean squared residual
                  CD |      0.162   Coefficient of determination
----------------------------------------------------------------------------
```

```
. estat teffects, standardized


Direct effects
----------------------------------------------------------------------------------------------
                             |                 OIM
                             |      Coef.   Std. Err.      z    P>|z|               Std. Coef.
-----------------------------+----------------------------------------------------------------
Structural                   |
  inpatient_hospitalizations |
                         age |  -.0002052   .0000915    -2.24   0.025               -.0117142
                   qci_score |   .0289917   .0012016    24.13   0.000                .1260711
                     rewards |   -.003556   .0000872   -40.76   0.000               -.2047813
  ---------------------------+----------------------------------------------------------------
  pdc                        |
  inpatient_hospitalizations |  -.0066559   .0055533    -1.20   0.231               -.0060334
                   er_visits |   .0048853    .007101     0.69   0.491                .0034248
          pcp_officevisits |  -.0003571   .0007904    -0.45   0.651               -.0031058
    specialist_office_visits |   .0000609     .00081     0.08   0.940                .0008078
    other_outpatientservices |  -.0015799   .0006234    -2.53   0.011               -.0259822
              pharmacy_fills |   .0126977    .000196    64.77   0.000                .3257658
                         age |   .0028451   .0000964    29.53   0.000                .1472301
                   qci_score |   -.007956   .0012664    -6.28   0.000               -.0313613
                     rewards |   .0026118   .0000944    27.67   0.000                .1363392
  ---------------------------+----------------------------------------------------------------
  er_visits                  |
                         age |  -.0005016   .0000714    -7.03   0.000               -.0370246
                   qci_score |   .0035294   .0009373     3.77   0.000                .0198452
                     rewards |  -.0026671    .000068   -39.20   0.000                -.198606
  ---------------------------+----------------------------------------------------------------
  pcp_officevisits           |
                         age |   .0102051   .0009022    11.31   0.000                .0607266
                   qci_score |   .0421582    .011844     3.56   0.000                .0191094
                     rewards |   .0002309   .0008599     0.27   0.788                .0013862
  ---------------------------+----------------------------------------------------------------
  specialist_office_visits   |
                         age |   .0146336   .0013781    10.62   0.000                .0570474
                   qci_score |   .0069804   .0180914     0.39   0.700                .0020729
                     rewards |   .0004574   .0013134     0.35   0.728                .0017987
```

other_outpatientservices					
age	.0238391	.0017035	13.99	0.000	.0750126
qci_score	.1281599	.0223629	5.73	0.000	.0307186
rewards	.0006867	.0016235	0.42	0.672	.0021798
pharmacy_fills					
age	.055894	.0026406	21.17	0.000	.1127401
qci_score	.3997155	.034666	11.53	0.000	.0614141
rewards	.0038239	.0025167	1.52	0.129	.0077806

Indirect effects

	Coef.	OIM Std. Err.	z	P>|z|	Std. Coef.
Structural					
inpatient_hospitalizations					
age	0	(no path)			0
qci_score	0	(no path)			0
rewards	0	(no path)			0
pdc					
inpatient_hospitalizations	0	(no path)			0
er_visits	0	(no path)			0
pcp_officevisits	0	(no path)			0
specialist_office_visits	0	(no path)			0
other_outpatientservices	0	(no path)			0
pharmacy_fills	0	(no path)			0
age	.0006682	.0000359	18.61	0.000	.0345792
qci_score	.0046826	.0004754	9.85	0.000	.0184581
rewards	.0000581	.0000406	1.43	0.153	.0030305
er_visits					
age	0	(no path)			0
qci_score	0	(no path)			0
rewards	0	(no path)			0

```
  pcp_officevisits           |
                         age |          0  (no path)                                        0
                   qci_score |          0  (no path)                                        0
                     rewards |          0  (no path)                                        0
  ---------------------------+----------------------------------------------------------------
  specialist_office_visits   |
                         age |          0  (no path)                                        0
                   qci_score |          0  (no path)                                        0
                     rewards |          0  (no path)                                        0
  ---------------------------+----------------------------------------------------------------
  other_outpatientservices   |
                         age |          0  (no path)                                        0
                   qci_score |          0  (no path)                                        0
                     rewards |          0  (no path)                                        0
  ---------------------------+----------------------------------------------------------------
  pharmacy_fills             |
                         age |          0  (no path)                                        0
                   qci_score |          0  (no path)                                        0
                     rewards |          0  (no path)                                        0
----------------------------------------------------------------------------------------------


Total effects
----------------------------------------------------------------------------------------------
                             |                 OIM
                             |      Coef.   Std. Err.      z    P>|z|               Std. Coef.
-----------------------------+----------------------------------------------------------------
Structural                   |
  inpatient_hospitalizations |
                         age |  -.0002052   .0000915    -2.24   0.025                -.0117142
                   qci_score |   .0289917   .0012016    24.13   0.000                 .1260711
                     rewards |   -.003556   .0000872   -40.76   0.000                -.2047813
  ---------------------------+----------------------------------------------------------------
  pdc                        |
  inpatient_hospitalizations |  -.0066559   .0055533    -1.20   0.231                -.0060334
                   er_visits |   .0048853    .007101     0.69   0.491                 .0034248
            pcp_officevisits |  -.0003571   .0007904    -0.45   0.651                -.0031058
    specialist_office_visits |   .0000609     .00081     0.08   0.940                 .0008078
    other_outpatientservices |  -.0015799   .0006234    -2.53   0.011                -.0259822
              pharmacy_fills |   .0126977    .000196    64.77   0.000                 .3257658
```

```
                      age |   .0035133   .0001013    34.69   0.000                  .1818093
                qci_score |  -.0032734   .0013297    -2.46   0.014                 -.0129031
                  rewards |   .0026698   .0000965    27.66   0.000                  .1393698
  ------------------------+----------------------------------------------------------------
  er_visits               |
                      age |  -.0005016   .0000714    -7.03   0.000                 -.0370246
                qci_score |   .0035294   .0009373     3.77   0.000                  .0198452
                  rewards |  -.0026671    .000068   -39.20   0.000                  -.198606
  ------------------------+----------------------------------------------------------------
  pcp_officevisits        |
                      age |   .0102051   .0009022    11.31   0.000                  .0607266
                qci_score |   .0421582    .011844     3.56   0.000                  .0191094
                  rewards |   .0002309   .0008599     0.27   0.788                  .0013862
  ------------------------+----------------------------------------------------------------
  specialist_office_visits |
                      age |   .0146336   .0013781    10.62   0.000                  .0570474
                qci_score |   .0069804   .0180914     0.39   0.700                  .0020729
                  rewards |   .0004574   .0013134     0.35   0.728                  .0017987
  ------------------------+----------------------------------------------------------------
  other_outpatientservices |
                      age |   .0238391   .0017035    13.99   0.000                  .0750126
                qci_score |   .1281599   .0223629     5.73   0.000                  .0307186
                  rewards |   .0006867   .0016235     0.42   0.672                  .0021798
  ------------------------+----------------------------------------------------------------
  pharmacy_fills          |
                      age |    .055894   .0026406    21.17   0.000                  .1127401
                qci_score |   .3997155    .034666    11.53   0.000                  .0614141
                  rewards |   .0038239   .0025167     1.52   0.129                  .0077806
------------------------------------------------------------------------------------------

. (note: file /Users/asim/Dropbox/Integrative Sustainable Solutions/TRUST APP/Cancer and Asthma Application/Health Corp
Data/SEMS version 2/SEM_M5.stsem not found)
```

MODEL 5

```
sem (inpatient_hospitalizations -> mpr, ) (er_visits -> mpr, ) (pcp_officevisits -> mpr, ) (specialist_office_visits ->
mpr, ) (other_outpatientservices -> mpr, ) (pharmacy_fills -> mpr, ) (age -> mpr
```

```
> , ) (age -> inpatient_hospitalizations, ) (age -> er_visits, ) (age -> pcp_officevisits, ) (age ->
specialist_office_visits, ) (age -> other_outpatientservices, ) (age -> pharmacy_fills, ) (qci_scor
> e -> mpr, ) (qci_score -> inpatient_hospitalizations, ) (qci_score -> er_visits, ) (qci_score -> pcp_officevisits, )
(qci_score -> specialist_office_visits, ) (qci_score -> other_outpatientservices,
>  ) (qci_score -> pharmacy_fills, ) (engagement_rate -> mpr, ) (engagement_rate -> inpatient_hospitalizations, )
(engagement_rate -> er_visits, ) (engagement_rate -> pcp_officevisits, ) (engagement_r
> ate -> specialist_office_visits, ) (engagement_rate -> other_outpatientservices, ) (engagement_rate ->
pharmacy_fills, ), standardized cov( age*qci_score age*engagement_rate qci_score*engagement_rat
> e) nocapslatent

Endogenous variables

Observed:  inpatient_hospitalizations mpr er_visits pcp_officevisits specialist_office_visits other_outpatientservices
pharmacy_fills

Exogenous variables

Observed:  age qci_score engagement_rate

Fitting target model:

Iteration 0:   log likelihood = -892280.31
Iteration 1:   log likelihood = -892280.31

Structural equation model                      Number of obs     =     37,359
Estimation method  = ml
Log likelihood     = -892280.31

-----------------------------------------------------------------------------------------------
                             |                 OIM
                Standardized |      Coef.   Std. Err.      z    P>|z|     [95% Conf. Interval]
-----------------------------+-----------------------------------------------------------------
Structural                   |
  inpatient_hospitalizations |
                         age |  -.0115089   .0053394    -2.16   0.031     -.021974   -.0010438
                   qci_score |   .1259906   .0053005    23.77   0.000     .1156019    .1363793
             engagement_rate |  -.0127983   .0051334    -2.49   0.013    -.0228595   -.0027371
                       _cons |   .0451134   .0192102     2.35   0.019      .007462    .0827648
  ---------------------------+-----------------------------------------------------------------
  mpr                        |
```

inpatient_hospitalizations	-.0339593	.0049109	-6.92	0.000	-.0435845	-.0243341
er_visits	-.0258236	.0048699	-5.30	0.000	-.0353684	-.0162788
pcp_officevisits	.015826	.0068168	2.32	0.020	.0024654	.0291866
specialist_office_visits	-.0106713	.0106576	-1.00	0.317	-.0315597	.0102172
other_outpatientservices	-.0078844	.0101603	-0.78	0.438	-.0277983	.0120294
pharmacy_fills	.3844366	.0045822	83.90	0.000	.3754558	.3934175
age	.1042404	.0049243	21.17	0.000	.094589	.1138918
qci_score	-.0139636	.0049504	-2.82	0.005	-.0236663	-.004261
engagement_rate	.0064819	.0047156	1.37	0.169	-.0027605	.0157244
_cons	.7576056	.0195373	38.78	0.000	.7193133	.795898
er_visits						
age	-.0368258	.0053733	-6.85	0.000	-.0473572	-.0262943
qci_score	.0197672	.0053757	3.68	0.000	.009231	.0303034
engagement_rate	-.0193069	.0051683	-3.74	0.000	-.0294366	-.0091772
_cons	.2216704	.019315	11.48	0.000	.1838137	.2595271
pcp_officevisits						
age	.0607252	.0053595	11.33	0.000	.0502208	.0712296
qci_score	.0191099	.0053677	3.56	0.000	.0085893	.0296305
engagement_rate	.00018	.0051616	0.03	0.972	-.0099364	.0102965
_cons	.6446561	.0196503	32.81	0.000	.6061422	.6831699
specialist_office_visits						
age	.0570456	.0053643	10.63	0.000	.0465318	.0675593
qci_score	.0020736	.0053723	0.39	0.700	-.008456	.0126031
engagement_rate	.0002934	.0051651	0.06	0.955	-.00983	.0104168
_cons	.4907647	.0195333	25.12	0.000	.4524801	.5290493
other_outpatientservices						
age	.0750104	.0053462	14.03	0.000	.064532	.0854888
qci_score	.0307194	.0053578	5.73	0.000	.0202183	.0412206
engagement_rate	.0003083	.0051534	0.06	0.952	-.0097922	.0104088
_cons	.1791063	.0193588	9.25	0.000	.1411637	.2170489
pharmacy_fills						
age	.1127323	.0052951	21.29	0.000	.102354	.1231106
qci_score	.0614172	.0053172	11.55	0.000	.0509956	.0718388
engagement_rate	.0007877	.005121	0.15	0.878	-.0092492	.0108245
_cons	.892638	.0199742	44.69	0.000	.8534892	.9317867

```
-----------------------------+----------------------------------------------------------------
                   mean(age)|   3.338398   .0132637   251.69   0.000     3.312401    3.364394
             mean(qci_score)|   1.297835   .0070221   184.82   0.000     1.284072    1.311598
       mean(engagement_rate)|   1.300017   .0070275   184.99   0.000     1.286244    1.313791
-----------------------------+----------------------------------------------------------------
var(e.inpatient_hospitaliz~s)|   .9846278   .0012632                     .9821551    .9871068
                  var(e.mpr)|   .8303914   .0036819                     .8232062    .8376393
            var(e.er_visits)|   .9982809   .0004283                     .9974418    .9991207
     var(e.pcp_officevisits)|   .9953088   .0007054                     .9939273    .9966923
var(e.specialist_office_vi~s)|   .9966763   .0005946                     .9955117    .9978423
var(e.other_outpatientserv~s)|    .992162   .0009089                     .9903822     .993945
       var(e.pharmacy_fills)|   .9797099    .001444                     .9768837    .9825442
                    var(age)|          1          .                            .           .
              var(qci_score)|          1          .                            .           .
        var(engagement_rate)|          1          .                            .           .
-----------------------------+----------------------------------------------------------------
          cov(age,qci_score)|   .2750612   .0047823    57.52   0.000     .2656881    .2844343
    cov(age,engagement_rate)|  -.0000374   .0051737    -0.01   0.994    -.0101777    .0101029
              cov(qci_score,|
             engagement_rate)|   4.90e-06   .0051737     0.00   0.999    -.0101354    .0101452
----------------------------------------------------------------------------------------------
Note: The LR test of model vs. saturated is not reported because the fitted
      model is not full rank.
.
. estat gof, stats(all)

----------------------------------------------------------------------------
Fit statistic        |      Value   Description
---------------------+------------------------------------------------------
Likelihood ratio     |
          chi2_ms(.) |          .   model vs. saturated
            p > chi2 |          .
         chi2_bs(42) |  97594.077   baseline vs. saturated
            p > chi2 |      0.000
---------------------+------------------------------------------------------
Population error     |
               RMSEA |          .   Root mean squared error of approximation
 90% CI, lower bound |      0.000
         upper bound |          .
              pclose |          .   Probability RMSEA <= 0.05
```

```
---------------------+----------------------------------------------------
Information criteria |
                 AIC |  1.785e+06   Akaike's information criterion
                 BIC |  1.785e+06   Bayesian information criterion
---------------------+----------------------------------------------------
Baseline comparison  |
                 CFI |      1.000   Comparative fit index
                 TLI |          .   Tucker-Lewis index
---------------------+----------------------------------------------------
Size of residuals    |
                SRMR |      0.191   Standardized root mean squared residual
                  CD |      0.063   Coefficient of determination
--------------------------------------------------------------------------

. estat teffects, standardized


Direct effects
------------------------------------------------------------------------------------------
                             |                 OIM
                             |      Coef.   Std. Err.      z    P>|z|              Std. Coef.
-----------------------------+------------------------------------------------------------
Structural                   |
  inpatient_hospitalizations |
                         age |  -.0002016   .0000935    -2.16   0.031              -.0115089
                   qci_score |   .0289732    .001228    23.59   0.000               .1259906
             engagement_rate |  -.0000185   7.43e-06    -2.49   0.013              -.0127983
  ---------------------------+------------------------------------------------------------
  mpr                        |
  inpatient_hospitalizations |  -.0469739   .0068012    -6.91   0.000              -.0339593
                   er_visits |  -.0461882   .0087134    -5.30   0.000              -.0258236
          pcp_officevisits |   .0022819   .0009829     2.32   0.020               .015826
    specialist_office_visits |   -.001008   .0010068    -1.00   0.317              -.0106713
    other_outpatientservices |  -.0006011   .0007748    -0.78   0.438              -.0078844
              pharmacy_fills |   .0187888   .0002437    77.09   0.000               .3844366
                         age |   .0025258   .0001198    21.08   0.000               .1042404
                   qci_score |  -.0044418   .0015747    -2.82   0.005              -.0139636
             engagement_rate |    .000013   9.44e-06     1.37   0.169               .0064819
  ---------------------------+------------------------------------------------------------
  er_visits                  |
```

```
                       age |  -.0004989   .0000728    -6.85   0.000                  -.0368258
                 qci_score |   .0035155   .0009562     3.68   0.000                   .0197672
           engagement_rate |  -.0000216   5.78e-06    -3.73   0.000                  -.0193069
  -------------------------+----------------------------------------------------------------
  pcp_officevisits         |
                       age |   .0102049   .0009022    11.31   0.000                   .0607252
                 qci_score |   .0421594    .011844     3.56   0.000                   .0191099
           engagement_rate |   2.50e-06   .0000717     0.03   0.972                     .00018
  -------------------------+----------------------------------------------------------------
  specialist_office_visits  |
                       age |   .0146331   .0013781    10.62   0.000                   .0570456
                 qci_score |   .0069828   .0180914     0.39   0.700                   .0020736
           engagement_rate |   6.22e-06   .0001095     0.06   0.955                   .0002934
  -------------------------+----------------------------------------------------------------
  other_outpatientservices  |
                       age |   .0238384   .0017035    13.99   0.000                   .0750104
                 qci_score |   .1281634   .0223629     5.73   0.000                   .0307194
           engagement_rate |   8.09e-06   .0001353     0.06   0.952                   .0003083
  -------------------------+----------------------------------------------------------------
  pharmacy_fills           |
                       age |   .0558901   .0026407    21.16   0.000                   .1127323
                 qci_score |   .3997354   .0346671    11.53   0.000                   .0614172
           engagement_rate |   .0000323   .0002097     0.15   0.878                   .0007877
---------------------------------------------------------------------------------------------


Indirect effects
---------------------------------------------------------------------------------------------
                             |                OIM
                             |      Coef.   Std. Err.      z    P>|z|               Std. Coef.
-----------------------------+---------------------------------------------------------------
Structural                   |
  inpatient_hospitalizations |
                         age |          0  (no path)                                         0
                   qci_score |          0  (no path)                                         0
             engagement_rate |          0  (no path)                                         0
  ---------------------------+---------------------------------------------------------------
  mpr                        |
  inpatient_hospitalizations |          0  (no path)                                         0
                   er_visits |          0  (no path)                                         0
```

```
           pcp_officevisits |          0  (no path)                                            0
   specialist_office_visits |          0  (no path)                                            0
   other_outpatientservices |          0  (no path)                                            0
             pharmacy_fills |          0  (no path)                                            0
                        age |   .0010768   .0000523    20.57   0.000                    .0444411
                  qci_score |   .0059993    .000691     8.68   0.000                    .0188601
            engagement_rate |   2.47e-06   3.98e-06     0.62   0.535                    .0012333
  --------------------------+---------------------------------------------------------------
  er_visits                 |
                        age |          0  (no path)                                            0
                  qci_score |          0  (no path)                                            0
            engagement_rate |          0  (no path)                                            0
  --------------------------+---------------------------------------------------------------
  pcp_officevisits          |
                        age |          0  (no path)                                            0
                  qci_score |          0  (no path)                                            0
            engagement_rate |          0  (no path)                                            0
  --------------------------+---------------------------------------------------------------
  specialist_office_visits  |
                        age |          0  (no path)                                            0
                  qci_score |          0  (no path)                                            0
            engagement_rate |          0  (no path)                                            0
  --------------------------+---------------------------------------------------------------
  other_outpatientservices  |
                        age |          0  (no path)                                            0
                  qci_score |          0  (no path)                                            0
            engagement_rate |          0  (no path)                                            0
  --------------------------+---------------------------------------------------------------
  pharmacy_fills            |
                        age |          0  (no path)                                            0
                  qci_score |          0  (no path)                                            0
            engagement_rate |          0  (no path)                                            0
----------------------------------------------------------------------------------------------


Total effects
----------------------------------------------------------------------------------------------
                            |                 OIM
                            |      Coef.   Std. Err.      z    P>|z|              Std. Coef.
----------------------------+-----------------------------------------------------------------
```

Structural					
inpatient_hospitalizations					
age	-.0002016	.0000935	-2.16	0.031	-.0115089
qci_score	.0289732	.001228	23.59	0.000	.1259906
engagement_rate	-.0000185	7.43e-06	-2.49	0.013	-.0127983
mpr					
inpatient_hospitalizations	-.0469739	.0068012	-6.91	0.000	-.0339593
er_visits	-.0461882	.0087134	-5.30	0.000	-.0258236
pcp_officevisits	.0022819	.0009829	2.32	0.020	.015826
specialist_office_visits	-.001008	.0010068	-1.00	0.317	-.0106713
other_outpatientservices	-.0006011	.0007748	-0.78	0.438	-.0078844
pharmacy_fills	.0187888	.0002437	77.09	0.000	.3844366
age	.0036026	.0001289	27.95	0.000	.1486815
qci_score	.0015575	.0016923	0.92	0.357	.0048965
engagement_rate	.0000154	.0000102	1.51	0.131	.0077152
er_visits					
age	-.0004989	.0000728	-6.85	0.000	-.0368258
qci_score	.0035155	.0009562	3.68	0.000	.0197672
engagement_rate	-.0000216	5.78e-06	-3.73	0.000	-.0193069
pcp_officevisits					
age	.0102049	.0009022	11.31	0.000	.0607252
qci_score	.0421594	.011844	3.56	0.000	.0191099
engagement_rate	2.50e-06	.0000717	0.03	0.972	.00018
specialist_office_visits					
age	.0146331	.0013781	10.62	0.000	.0570456
qci_score	.0069828	.0180914	0.39	0.700	.0020736
engagement_rate	6.22e-06	.0001095	0.06	0.955	.0002934
other_outpatientservices					
age	.0238384	.0017035	13.99	0.000	.0750104
qci_score	.1281634	.0223629	5.73	0.000	.0307194
engagement_rate	8.09e-06	.0001353	0.06	0.952	.0003083
pharmacy_fills					
age	.0558901	.0026407	21.16	0.000	.1127323
qci_score	.3997354	.0346671	11.53	0.000	.0614172

```
            engagement_rate |   .0000323   .0002097     0.15   0.878                .0007877
----------------------------------------------------------------------------------------------

. (note: file /Users/asim/Dropbox/Integrative Sustainable Solutions/TRUST APP/Cancer and Asthma Application/Health Corp
Data/SEMS version 2/SEM_M6.stsem not found)
```

MODEL 6

```
sem (inpatient_hospitalizations -> pdc, ) (er_visits -> pdc, ) (pcp_officevisits -> pdc, ) (specialist_office_visits ->
pdc, ) (other_outpatientservices -> pdc, ) (pharmacy_fills -> pdc, ) (age -> pdc
> , ) (age -> inpatient_hospitalizations, ) (age -> er_visits, ) (age -> pcp_officevisits, ) (age ->
specialist_office_visits, ) (age -> other_outpatientservices, ) (age -> pharmacy_fills, ) (qci_scor
> e -> pdc, ) (qci_score -> inpatient_hospitalizations, ) (qci_score -> er_visits, ) (qci_score -> pcp_officevisits, )
(qci_score -> specialist_office_visits, ) (qci_score -> other_outpatientservices,
>  ) (qci_score -> pharmacy_fills, ) (engagement_rate -> pdc, ) (engagement_rate -> inpatient_hospitalizations, )
(engagement_rate -> er_visits, ) (engagement_rate -> pcp_officevisits, ) (engagement_r
> ate -> specialist_office_visits, ) (engagement_rate -> other_outpatientservices, ) (engagement_rate ->
pharmacy_fills, ), standardized cov( age*qci_score age*engagement_rate qci_score*engagement_rat
> e) nocapslatent

Endogenous variables

Observed:  inpatient_hospitalizations pdc er_visits pcp_officevisits specialist_office_visits other_outpatientservices
pharmacy_fills

Exogenous variables

Observed:  age qci_score engagement_rate

Fitting target model:

Iteration 0:   log likelihood = -884509.38
Iteration 1:   log likelihood = -884509.38

Structural equation model                        Number of obs     =     37,359
Estimation method  = ml
```

```
Log likelihood     = -884509.38

------------------------------------------------------------------------------------------------
                             |                 OIM
                Standardized |      Coef.   Std. Err.      z    P>|z|     [95% Conf. Interval]
-----------------------------+------------------------------------------------------------------
Structural                   |
  inpatient_hospitalizations |
                         age | -.0115089   .0053394    -2.16   0.031     -.021974   -.0010438
                   qci_score |  .1259906   .0053005    23.77   0.000     .1156019    .1363793
             engagement_rate | -.0127983   .0051334    -2.49   0.013    -.0228595   -.0027371
                       _cons |  .0451134   .0192102     2.35   0.019      .007462    .0827648
  ---------------------------+------------------------------------------------------------------
  pdc                        |
  inpatient_hospitalizations | -.0302308   .0049997    -6.05   0.000      -.04003   -.0204315
                   er_visits | -.0190096   .0049575    -3.83   0.000    -.0287262    -.009293
            pcp_officevisits | -.0007109   .0069384    -0.10   0.918    -.0143099     .012888
    specialist_office_visits | -.0093499   .0108484    -0.86   0.389    -.0306124    .0119126
    other_outpatientservices | -.0154537   .0103391    -1.49   0.135    -.0357179    .0048106
              pharmacy_fills |  .3293753   .0047728    69.01   0.000     .3200208    .3387298
                         age |  .1451174   .0049903    29.08   0.000     .1353366    .1548981
                   qci_score | -.0283777   .0050376    -5.63   0.000    -.0382512   -.0185043
             engagement_rate |  .0078895   .0047995     1.64   0.100    -.0015173    .0172963
                       _cons |  1.170433    .020606    56.80   0.000     1.130046     1.21082
  ---------------------------+------------------------------------------------------------------
  er_visits                  |
                         age | -.0368258   .0053733    -6.85   0.000    -.0473572   -.0262943
                   qci_score |  .0197672   .0053757     3.68   0.000      .009231    .0303034
             engagement_rate | -.0193069   .0051683    -3.74   0.000    -.0294366   -.0091772
                       _cons |  .2216704    .019315    11.48   0.000     .1838137    .2595271
  ---------------------------+------------------------------------------------------------------
  pcp_officevisits           |
                         age |  .0607252   .0053595    11.33   0.000     .0502208    .0712296
                   qci_score |  .0191099   .0053677     3.56   0.000     .0085893    .0296305
             engagement_rate |    .00018   .0051616     0.03   0.972    -.0099364    .0102965
                       _cons |  .6446561   .0196503    32.81   0.000     .6061422    .6831699
  ---------------------------+------------------------------------------------------------------
  specialist_office_visits   |
                         age |  .0570456   .0053643    10.63   0.000     .0465318    .0675593
                   qci_score |  .0020736   .0053723     0.39   0.700     -.008456    .0126031
```

```
            engagement_rate |   .0002934   .0051651     0.06   0.955     -.00983    .0104168
                      _cons |   .4907647   .0195333    25.12   0.000    .4524801    .5290493
  --------------------------+----------------------------------------------------------------
  other_outpatientservices  |
                        age |   .0750104   .0053462    14.03   0.000     .064532    .0854888
                  qci_score |   .0307194   .0053578     5.73   0.000    .0202183    .0412206
            engagement_rate |   .0003083   .0051534     0.06   0.952   -.0097922    .0104088
                      _cons |   .1791063   .0193588     9.25   0.000    .1411637    .2170489
  --------------------------+----------------------------------------------------------------
  pharmacy_fills            |
                        age |   .1127323   .0052951    21.29   0.000     .102354    .1231106
                  qci_score |   .0614172   .0053172    11.55   0.000    .0509956    .0718388
            engagement_rate |   .0007877    .005121     0.15   0.878   -.0092492    .0108245
                      _cons |    .892638   .0199742    44.69   0.000    .8534892    .9317867
----------------------------+----------------------------------------------------------------
                  mean(age)|   3.338398   .0132637   251.69   0.000    3.312401    3.364394
            mean(qci_score)|   1.297835   .0070221   184.82   0.000    1.284072    1.311598
      mean(engagement_rate)|   1.300017   .0070275   184.99   0.000    1.286244    1.313791
----------------------------+----------------------------------------------------------------
var(e.inpatient_hospitaliz~s)|   .9846278   .0012632                      .9821551    .9871068
               var(e.pdc)|   .8602023   .0034688                      .8534304    .8670279
         var(e.er_visits)|   .9982809   .0004283                      .9974418    .9991207
    var(e.pcp_officevisits)|   .9953088   .0007054                      .9939273    .9966923
var(e.specialist_office_vi~s)|   .9966763   .0005946                      .9955117    .9978423
var(e.other_outpatientserv~s)|    .992162   .0009089                      .9903822     .993945
      var(e.pharmacy_fills)|   .9797099    .001444                      .9768837    .9825442
                   var(age)|          1          .                             .           .
             var(qci_score)|          1          .                             .           .
       var(engagement_rate)|          1          .                             .           .
----------------------------+----------------------------------------------------------------
       cov(age,qci_score)|   .2750612   .0047823    57.52   0.000    .2656881    .2844343
   cov(age,engagement_rate)|  -.0000374   .0051737    -0.01   0.994   -.0101777    .0101029
             cov(qci_score,|
            engagement_rate)|   4.90e-06   .0051737     0.00   0.999   -.0101354    .0101452
---------------------------------------------------------------------------------------------
Note: The LR test of model vs. saturated is not reported because the fitted
      model is not full rank.
.
. estat gof, stats(all)
```

```
----------------------------------------------------------------------------
Fit statistic        |      Value   Description
---------------------+------------------------------------------------------
Likelihood ratio     |
          chi2_ms(.) |          .   model vs. saturated
            p > chi2 |          .
         chi2_bs(42) |  96203.408   baseline vs. saturated
            p > chi2 |      0.000
---------------------+------------------------------------------------------
Population error     |
               RMSEA |          .   Root mean squared error of approximation
 90% CI, lower bound |      0.000
         upper bound |          .
              pclose |          .   Probability RMSEA <= 0.05
---------------------+------------------------------------------------------
Information criteria |
                 AIC |  1.769e+06   Akaike's information criterion
                 BIC |  1.770e+06   Bayesian information criterion
---------------------+------------------------------------------------------
Baseline comparison  |
                 CFI |      1.000   Comparative fit index
                 TLI |          .   Tucker-Lewis index
---------------------+------------------------------------------------------
Size of residuals    |
                SRMR |      0.191   Standardized root mean squared residual
                  CD |      0.072   Coefficient of determination
----------------------------------------------------------------------------

. estat teffects, standardized


Direct effects
----------------------------------------------------------------------------------------------
                             |                OIM
                             |      Coef.   Std. Err.      z    P>|z|               Std. Coef.
-----------------------------+----------------------------------------------------------------
Structural                   |
  inpatient_hospitalizations |
                         age |  -.0002016    .0000935    -2.16   0.031               -.0115089
                   qci_score |   .0289732    .001228    23.59   0.000                .1259906
```

engagement_rate	-.0000185	7.43e-06	-2.49	0.013	-.0127983
pdc					
inpatient_hospitalizations	-.0333696	.0055239	-6.04	0.000	-.0302308
er_visits	-.0271326	.0070771	-3.83	0.000	-.0190096
pcp_officevisits	-.0000818	.0007983	-0.10	0.918	-.0007109
specialist_office_visits	-.0007048	.0008177	-0.86	0.389	-.0093499
other_outpatientservices	-.0009402	.0006293	-1.49	0.135	-.0154537
pharmacy_fills	.012846	.000198	64.89	0.000	.3293753
age	.002806	.0000973	28.83	0.000	.1451174
qci_score	-.0072034	.001279	-5.63	0.000	-.0283777
engagement_rate	.0000126	7.67e-06	1.64	0.100	.0078895
er_visits					
age	-.0004989	.0000728	-6.85	0.000	-.0368258
qci_score	.0035155	.0009562	3.68	0.000	.0197672
engagement_rate	-.0000216	5.78e-06	-3.73	0.000	-.0193069
pcp_officevisits					
age	.0102049	.0009022	11.31	0.000	.0607252
qci_score	.0421594	.011844	3.56	0.000	.0191099
engagement_rate	2.50e-06	.0000717	0.03	0.972	.00018
specialist_office_visits					
age	.0146331	.0013781	10.62	0.000	.0570456
qci_score	.0069828	.0180914	0.39	0.700	.0020736
engagement_rate	6.22e-06	.0001095	0.06	0.955	.0002934
other_outpatientservices					
age	.0238384	.0017035	13.99	0.000	.0750104
qci_score	.1281634	.0223629	5.73	0.000	.0307194
engagement_rate	8.09e-06	.0001353	0.06	0.952	.0003083
pharmacy_fills					
age	.0558901	.0026407	21.16	0.000	.1127323
qci_score	.3997354	.0346671	11.53	0.000	.0614172
engagement_rate	.0000323	.0002097	0.15	0.878	.0007877

Indirect effects

	Coef.	OIM Std. Err.	z	P>|z|	Std. Coef.
**Structural**					
inpatient_hospitalizations					
age	0	(no path)			0
qci_score	0	(no path)			0
engagement_rate	0	(no path)			0
pdc					
inpatient_hospitalizations	0	(no path)			0
er_visits	0	(no path)			0
pcp_officevisits	0	(no path)			0
specialist_office_visits	0	(no path)			0
other_outpatientservices	0	(no path)			0
pharmacy_fills	0	(no path)			0
age	.0007047	.0000364	19.35	0.000	.0364435
qci_score	.0039439	.0004818	8.19	0.000	.0155371
engagement_rate	1.61e-06	2.72e-06	0.59	0.555	.0010057
er_visits					
age	0	(no path)			0
qci_score	0	(no path)			0
engagement_rate	0	(no path)			0
pcp_officevisits					
age	0	(no path)			0
qci_score	0	(no path)			0
engagement_rate	0	(no path)			0
specialist_office_visits					
age	0	(no path)			0
qci_score	0	(no path)			0
engagement_rate	0	(no path)			0
other_outpatientservices					
age	0	(no path)			0
qci_score	0	(no path)			0

```
            engagement_rate |          0  (no path)                                          0
  --------------------------+-----------------------------------------------------------------
  pharmacy_fills            |
                        age |          0  (no path)                                          0
                  qci_score |          0  (no path)                                          0
            engagement_rate |          0  (no path)                                          0
--------------------------------------------------------------------------------------------------


Total effects
--------------------------------------------------------------------------------------------------
                            |                 OIM
                            |      Coef.   Std. Err.      z    P>|z|               Std. Coef.
----------------------------+---------------------------------------------------------------------
Structural                  |
  inpatient_hospitalizations |
                        age |  -.0002016   .0000935    -2.16   0.031                -.0115089
                  qci_score |   .0289732    .001228    23.59   0.000                 .1259906
            engagement_rate |  -.0000185   7.43e-06    -2.49   0.013                -.0127983
  --------------------------+-----------------------------------------------------------------
  pdc                       |
  inpatient_hospitalizations |  -.0333696   .0055239    -6.04   0.000                -.0302308
                  er_visits |  -.0271326   .0070771    -3.83   0.000                -.0190096
            pcp_officevisits |  -.0000818   .0007983    -0.10   0.918                -.0007109
    specialist_office_visits |  -.0007048   .0008177    -0.86   0.389                -.0093499
    other_outpatientservices |  -.0009402   .0006293    -1.49   0.135                -.0154537
             pharmacy_fills |    .012846    .000198    64.89   0.000                 .3293753
                        age |   .0035106   .0001024    34.29   0.000                 .1815608
                  qci_score |  -.0032595    .001344    -2.43   0.015                -.0128407
            engagement_rate |   .0000142   8.13e-06     1.75   0.081                 .0088952
  --------------------------+-----------------------------------------------------------------
  er_visits                 |
                        age |  -.0004989   .0000728    -6.85   0.000                -.0368258
                  qci_score |   .0035155   .0009562     3.68   0.000                 .0197672
            engagement_rate |  -.0000216   5.78e-06    -3.73   0.000                -.0193069
  --------------------------+-----------------------------------------------------------------
  pcp_officevisits          |
                        age |   .0102049   .0009022    11.31   0.000                 .0607252
                  qci_score |   .0421594    .011844     3.56   0.000                 .0191099
            engagement_rate |   2.50e-06   .0000717     0.03   0.972                   .00018
```

```
  --------------------------+----------------------------------------------------------------
  specialist_office_visits   |
                        age |   .0146331   .0013781    10.62   0.000                 .0570456
                  qci_score |   .0069828   .0180914     0.39   0.700                 .0020736
            engagement_rate |   6.22e-06   .0001095     0.06   0.955                 .0002934
  --------------------------+----------------------------------------------------------------
  other_outpatientservices   |
                        age |   .0238384   .0017035    13.99   0.000                 .0750104
                  qci_score |   .1281634   .0223629     5.73   0.000                 .0307194
            engagement_rate |   8.09e-06   .0001353     0.06   0.952                 .0003083
  --------------------------+----------------------------------------------------------------
  pharmacy_fills             |
                        age |   .0558901   .0026407    21.16   0.000                 .1127323
                  qci_score |   .3997354   .0346671    11.53   0.000                 .0614172
            engagement_rate |   .0000323   .0002097     0.15   0.878                 .0007877
----------------------------------------------------------------------------------------------

. (note: file /Users/asim/Dropbox/Integrative Sustainable Solutions/TRUST APP/Cancer and Asthma Application/Health Corp
Data/SEMS version 2/SEM_M7.stsem not found)
```

MODEL 7

```
sem (inpatient_hospitalizations -> mpr, ) (er_visits -> mpr, ) (pcp_officevisits -> mpr, ) (specialist_office_visits ->
mpr, ) (other_outpatientservices -> mpr, ) (pharmacy_fills -> mpr, ) (age -> mpr
> , ) (age -> inpatient_hospitalizations, ) (age -> er_visits, ) (age -> pcp_officevisits, ) (age ->
specialist_office_visits, ) (age -> other_outpatientservices, ) (age -> pharmacy_fills, ) (qci_scor
> e -> mpr, ) (qci_score -> inpatient_hospitalizations, ) (qci_score -> er_visits, ) (qci_score -> pcp_officevisits, )
(qci_score -> specialist_office_visits, ) (qci_score -> other_outpatientservices,
>  ) (qci_score -> pharmacy_fills, ) (engagement_rate -> mpr, ) (engagement_rate -> inpatient_hospitalizations, )
(engagement_rate -> er_visits, ) (engagement_rate -> pcp_officevisits, ) (engagement_r
> ate -> specialist_office_visits, ) (engagement_rate -> other_outpatientservices, ) (engagement_rate ->
pharmacy_fills, ) (rewards -> mpr, ) (rewards -> inpatient_hospitalizations, ) (rewards -> er_v
> isits, ) (rewards -> pcp_officevisits, ) (rewards -> specialist_office_visits, ) (rewards ->
other_outpatientservices, ) (rewards -> pharmacy_fills, ), standardized cov( age*qci_score age*engagement
> _rate qci_score*engagement_rate engagement_rate*rewards rewards*age rewards*qci_score) nocapslatent

Endogenous variables
```

```
Observed:  inpatient_hospitalizations mpr er_visits pcp_officevisits specialist_office_visits other_outpatientservices
pharmacy_fills

Exogenous variables

Observed:  age qci_score engagement_rate rewards

Fitting target model:

Iteration 0:   log likelihood = -1044572.4
Iteration 1:   log likelihood = -1044572.4

Structural equation model                      Number of obs     =     37,359
Estimation method  = ml
Log likelihood     = -1044572.4

---------------------------------------------------------------------------------------------
                             |                 OIM
                Standardized |      Coef.   Std. Err.      z    P>|z|     [95% Conf. Interval]
-----------------------------+---------------------------------------------------------------
Structural                   |
  inpatient_hospitalizations |
                         age |  -.0117148   .0052245    -2.24   0.025    -.0219546   -.0014749
                   qci_score |   .1260713    .005188    24.30   0.000      .115903    .1362397
             engagement_rate |  -.0127361   .0050229    -2.54   0.011    -.0225808   -.0028914
                     rewards |  -.2047775   .0049186   -41.63   0.000    -.2144177   -.1951372
                       _cons |   .4551753   .0212149    21.46   0.000     .4135949    .4967557
  ---------------------------+---------------------------------------------------------------
  mpr                        |
  inpatient_hospitalizations |  -.0120157   .0049528    -2.43   0.015     -.021723   -.0023084
                   er_visits |  -.0054108   .0048998    -1.10   0.269    -.0150142    .0041927
            pcp_officevisits |   .0136612   .0067647     2.02   0.043     .0004027    .0269198
    specialist_office_visits |  -.0014452   .0105818    -0.14   0.891     -.022185    .0192947
    other_outpatientservices |  -.0174484   .0100834    -1.73   0.084    -.0372116    .0023148
              pharmacy_fills |   .3812621   .0045603    83.60   0.000      .372324    .3902002
                         age |   .1061591   .0048855    21.73   0.000     .0965837    .1157344
                   qci_score |  -.0166658    .004913    -3.39   0.001    -.0262952   -.0070364
             engagement_rate |   .0071283   .0046789     1.52   0.128    -.0020422    .0162988
                     rewards |   .1232698   .0048269    25.54   0.000     .1138093    .1327303
                       _cons |   .5074725   .0215687    23.53   0.000     .4651985    .5497464
```

er_visits						
age	-.0370254	.0052662	-7.03	0.000	-.0473469	-.0267039
qci_score	.0198455	.0052685	3.77	0.000	.0095195	.0301715
engagement_rate	-.0192466	.0050652	-3.80	0.000	-.0291743	-.009319
rewards	-.1986002	.0049654	-40.00	0.000	-.2083321	-.1888682
_cons	.6193624	.0212275	29.18	0.000	.5777573	.6609675
pcp_officevisits						
age	.0607266	.0053595	11.33	0.000	.0502222	.071231
qci_score	.0191094	.0053677	3.56	0.000	.0085888	.02963
engagement_rate	.0001796	.0051616	0.03	0.972	-.0099369	.0102961
rewards	.0013861	.0051616	0.27	0.788	-.0087304	.0115026
_cons	.6418804	.0222031	28.91	0.000	.5983632	.6853977
specialist_office_visits						
age	.0570474	.0053642	10.63	0.000	.0465337	.0675611
qci_score	.0020729	.0053723	0.39	0.700	-.0084567	.0126024
engagement_rate	.0002929	.0051651	0.06	0.955	-.0098305	.0104163
rewards	.0017986	.0051651	0.35	0.728	-.0083248	.011922
_cons	.487163	.0221029	22.04	0.000	.4438422	.5304838
other_outpatientservices						
age	.0750126	.0053462	14.03	0.000	.0645342	.085491
qci_score	.0307186	.0053578	5.73	0.000	.0202174	.0412197
engagement_rate	.0003077	.0051534	0.06	0.952	-.0097928	.0104081
rewards	.0021797	.0051534	0.42	0.672	-.0079207	.0122801
_cons	.1747415	.0219364	7.97	0.000	.1317469	.2177361
pharmacy_fills						
age	.1127401	.005295	21.29	0.000	.1023621	.1231181
qci_score	.0614141	.0053171	11.55	0.000	.0509929	.0718354
engagement_rate	.0007853	.0051208	0.15	0.878	-.0092513	.0108219
rewards	.0077803	.0051206	1.52	0.129	-.0022559	.0178166
_cons	.877058	.0224515	39.06	0.000	.8330539	.9210621
mean(age)	3.338398	.0132637	251.69	0.000	3.312401	3.364394
mean(qci_score)	1.297835	.0070221	184.82	0.000	1.284072	1.311598
mean(engagement_rate)	1.300017	.0070275	184.99	0.000	1.286244	1.313791
mean(rewards)	2.000027	.0089612	223.19	0.000	1.982463	2.01759

```
-----------------------------+----------------------------------------------------------------
var(e.inpatient_hospitaliz~s)|   .9426941   .0023351                      .9381285    .9472819
                 var(e.mpr)|   .8174848   .0037566                      .8101551    .8248808
           var(e.er_visits)|   .9588389   .0020129                      .9549018    .9627922
    var(e.pcp_officevisits)|   .9953069   .0007055                      .9939251    .9966907
var(e.specialist_office_vi~s)|   .9966731   .0005948                      .9955079    .9978397
var(e.other_outpatientserv~s)|   .9921573   .0009092                      .9903769    .9939408
       var(e.pharmacy_fills)|   .9796493   .0014461                      .9768192    .9824877
                   var(age)|          1          .                             .           .
             var(qci_score)|          1          .                             .           .
       var(engagement_rate)|          1          .                             .           .
               var(rewards)|          1          .                             .           .
-----------------------------+----------------------------------------------------------------
         cov(age,qci_score)|   .2750612   .0047823    57.52   0.000     .2656881    .2844343
   cov(age,engagement_rate)|  -.0000374   .0051737    -0.01   0.994    -.0101777    .0101029
           cov(age,rewards)|  -.0008967   .0051737    -0.17   0.862     -.011037    .0092436
             cov(qci_score,|
           engagement_rate)|   4.90e-06   .0051737     0.00   0.999    -.0101354    .0101452
     cov(qci_score,rewards)|   .0001177   .0051737     0.02   0.982    -.0100226     .010258
 cov(engagement_rate,rewards)|   .0003037   .0051737     0.06   0.953    -.0098366     .010444
----------------------------------------------------------------------------------------------
Note: The LR test of model vs. saturated is not reported because the fitted
      model is not full rank.
.
. (note: file /Users/asim/Dropbox/Integrative Sustainable Solutions/TRUST APP/Cancer and Asthma Application/Health Corp
Data/SEMS version 2/SEM_M8.stsem not found)
estat gof, stats(all)

----------------------------------------------------------------------------
Fit statistic        |      Value   Description
---------------------+------------------------------------------------------
Likelihood ratio     |
          chi2_ms(.) |          .   model vs. saturated
            p > chi2 |          .
         chi2_bs(49) | 100933.301   baseline vs. saturated
            p > chi2 |      0.000
---------------------+------------------------------------------------------
Population error     |
               RMSEA |          .   Root mean squared error of approximation
 90% CI, lower bound |      0.000
```

```
        upper bound |          .
             pclose |          .   Probability RMSEA <= 0.05
--------------------+---------------------------------------------
Information criteria |
                AIC |  2.089e+06   Akaike's information criterion
                BIC |  2.090e+06   Bayesian information criterion
--------------------+---------------------------------------------
Baseline comparison  |
                CFI |      1.000   Comparative fit index
                TLI |          .   Tucker-Lewis index
--------------------+---------------------------------------------
Size of residuals    |
               SRMR |      0.174   Standardized root mean squared residual
                 CD |      0.151   Coefficient of determination
------------------------------------------------------------------

. estat teffects, standardized


Direct effects
------------------------------------------------------------------------------------------------
                             |                 OIM
                             |      Coef.   Std. Err.      z    P>|z|               Std. Coef.
-----------------------------+------------------------------------------------------------------
Structural                   |
  inpatient_hospitalizations |
                         age |  -.0002052   .0000915    -2.24   0.025                -.0117148
                   qci_score |   .0289918   .0012015    24.13   0.000                 .1260713
             engagement_rate |  -.0000184   7.27e-06    -2.54   0.011                -.0127361
                     rewards |  -.0035559   .0000872   -40.77   0.000                -.2047775
  ---------------------------+------------------------------------------------------------------
  mpr                        |
  inpatient_hospitalizations |  -.0166083   .0068481    -2.43   0.015                -.0120157
                   er_visits |  -.0096706   .0087577    -1.10   0.269                -.0054108
            pcp_officevisits |   .0019683   .0009746     2.02   0.043                 .0136612
    specialist_office_visits |  -.0001364   .0009988    -0.14   0.891                -.0014452
    other_outpatientservices |  -.0013293   .0007687    -1.73   0.084                -.0174484
             pharmacy_fills |   .0186198   .0002417    77.03   0.000                 .3812621
                         age |   .0025704   .0001188    21.63   0.000                 .1061591
                   qci_score |  -.0052974   .0015617    -3.39   0.001                -.0166658
```

engagement_rate	.0000143	9.36e-06	1.52	0.128	.0071283
rewards	.0029587	.0001164	25.42	0.000	.1232698
er_visits					
age	-.0005016	.0000714	-7.03	0.000	-.0370254
qci_score	.0035294	.0009371	3.77	0.000	.0198455
engagement_rate	-.0000215	5.67e-06	-3.80	0.000	-.0192466
rewards	-.0026671	.000068	-39.20	0.000	-.1986002
pcp_officevisits					
age	.0102051	.0009022	11.31	0.000	.0607266
qci_score	.0421582	.011844	3.56	0.000	.0191094
engagement_rate	2.49e-06	.0000717	0.03	0.972	.0001796
rewards	.0002309	.0008599	0.27	0.788	.0013861
specialist_office_visits					
age	.0146336	.0013781	10.62	0.000	.0570474
qci_score	.0069804	.0180914	0.39	0.700	.0020729
engagement_rate	6.21e-06	.0001095	0.06	0.955	.0002929
rewards	.0004574	.0013134	0.35	0.728	.0017986
other_outpatientservices					
age	.0238391	.0017035	13.99	0.000	.0750126
qci_score	.1281599	.0223629	5.73	0.000	.0307186
engagement_rate	8.08e-06	.0001353	0.06	0.952	.0003077
rewards	.0006867	.0016235	0.42	0.672	.0021797
pharmacy_fills					
age	.055894	.0026406	21.17	0.000	.1127401
qci_score	.3997155	.034666	11.53	0.000	.0614141
engagement_rate	.0000322	.0002097	0.15	0.878	.0007853
rewards	.0038238	.0025167	1.52	0.129	.0077803

Indirect effects

	Coef.	OIM Std. Err.	z	P>|z|	Std. Coef.

```
Structural                   |
  inpatient_hospitalizations |
                         age |          0  (no path)                                                  0
                   qci_score |          0  (no path)                                                  0
             engagement_rate |          0  (no path)                                                  0
                     rewards |          0  (no path)                                                  0
  ---------------------------+-----------------------------------------------------------------
  mpr                        |
  inpatient_hospitalizations |          0  (no path)                                                  0
                   er_visits |          0  (no path)                                                  0
            pcp_officevisits |          0  (no path)                                                  0
    specialist_office_visits |          0  (no path)                                                  0
    other_outpatientservices |          0  (no path)                                                  0
              pharmacy_fills |          0  (no path)                                                  0
                         age |   .0010354   .0000517    20.04   0.000                         .0427629
                   qci_score |   .0068387   .0006823    10.02   0.000                         .0215147
             engagement_rate |   1.11e-06   3.92e-06     0.28   0.778                         .0005532
                     rewards |   .0001555   .0000561     2.77   0.006                         .0064798
  ---------------------------+-----------------------------------------------------------------
  er_visits                  |
                         age |          0  (no path)                                                  0
                   qci_score |          0  (no path)                                                  0
             engagement_rate |          0  (no path)                                                  0
                     rewards |          0  (no path)                                                  0
  ---------------------------+-----------------------------------------------------------------
  pcp_officevisits           |
                         age |          0  (no path)                                                  0
                   qci_score |          0  (no path)                                                  0
             engagement_rate |          0  (no path)                                                  0
                     rewards |          0  (no path)                                                  0
  ---------------------------+-----------------------------------------------------------------
  specialist_office_visits   |
                         age |          0  (no path)                                                  0
                   qci_score |          0  (no path)                                                  0
             engagement_rate |          0  (no path)                                                  0
                     rewards |          0  (no path)                                                  0
  ---------------------------+-----------------------------------------------------------------
  other_outpatientservices   |
                         age |          0  (no path)                                                  0
                   qci_score |          0  (no path)                                                  0
```

```
            engagement_rate |          0  (no path)                                            0
                    rewards |          0  (no path)                                            0
  --------------------------+----------------------------------------------------------------
  pharmacy_fills            |
                        age |          0  (no path)                                            0
                  qci_score |          0  (no path)                                            0
            engagement_rate |          0  (no path)                                            0
                    rewards |          0  (no path)                                            0
----------------------------------------------------------------------------------------------


Total effects
----------------------------------------------------------------------------------------------
                            |                 OIM
                            |      Coef.   Std. Err.      z    P>|z|               Std. Coef.
----------------------------+-----------------------------------------------------------------
Structural                  |
  inpatient_hospitalizations |
                        age |  -.0002052   .0000915    -2.24   0.025                -.0117148
                  qci_score |   .0289918   .0012015    24.13   0.000                 .1260713
            engagement_rate |  -.0000184   7.27e-06    -2.54   0.011                -.0127361
                    rewards |  -.0035559   .0000872   -40.77   0.000                -.2047775
  --------------------------+-----------------------------------------------------------------
  mpr                       |
  inpatient_hospitalizations |  -.0166083   .0068481    -2.43   0.015                -.0120157
                  er_visits |  -.0096706   .0087577    -1.10   0.269                -.0054108
            pcp_officevisits |   .0019683   .0009746     2.02   0.043                 .0136612
    specialist_office_visits |  -.0001364   .0009988    -0.14   0.891                -.0014452
    other_outpatientservices |  -.0013293   .0007687    -1.73   0.084                -.0174484
             pharmacy_fills |   .0186198   .0002417    77.03   0.000                 .3812621
                        age |   .0036058   .0001277    28.24   0.000                  .148922
                  qci_score |   .0015413   .0016764     0.92   0.358                  .004849
            engagement_rate |   .0000154   .0000101     1.51   0.130                 .0076815
                    rewards |   .0031142   .0001217    25.59   0.000                 .1297496
  --------------------------+-----------------------------------------------------------------
  er_visits                 |
                        age |  -.0005016   .0000714    -7.03   0.000                -.0370254
                  qci_score |   .0035294   .0009371     3.77   0.000                 .0198455
            engagement_rate |  -.0000215   5.67e-06    -3.80   0.000                -.0192466
                    rewards |  -.0026671    .000068   -39.20   0.000                -.1986002
```

```
  --------------------------+----------------------------------------------------------------
  pcp_officevisits          |
                        age |   .0102051   .0009022    11.31   0.000                 .0607266
                  qci_score |   .0421582    .011844     3.56   0.000                 .0191094
            engagement_rate |   2.49e-06   .0000717     0.03   0.972                 .0001796
                    rewards |   .0002309   .0008599     0.27   0.788                 .0013861
  --------------------------+----------------------------------------------------------------
  specialist_office_visits  |
                        age |   .0146336   .0013781    10.62   0.000                 .0570474
                  qci_score |   .0069804   .0180914     0.39   0.700                 .0020729
            engagement_rate |   6.21e-06   .0001095     0.06   0.955                 .0002929
                    rewards |   .0004574   .0013134     0.35   0.728                 .0017986
  --------------------------+----------------------------------------------------------------
  other_outpatientservices  |
                        age |   .0238391   .0017035    13.99   0.000                 .0750126
                  qci_score |   .1281599   .0223629     5.73   0.000                 .0307186
            engagement_rate |   8.08e-06   .0001353     0.06   0.952                 .0003077
                    rewards |   .0006867   .0016235     0.42   0.672                 .0021797
  --------------------------+----------------------------------------------------------------
  pharmacy_fills            |
                        age |    .055894   .0026406    21.17   0.000                 .1127401
                  qci_score |   .3997155    .034666    11.53   0.000                 .0614141
            engagement_rate |   .0000322   .0002097     0.15   0.878                 .0007853
                    rewards |   .0038238   .0025167     1.52   0.129                 .0077803
---------------------------------------------------------------------------------------------
```

MODEL 8

```
. sem (inpatient_hospitalizations -> pdc, ) (er_visits -> pdc, ) (pcp_officevisits -> pdc, ) (specialist_office_visits
-> pdc, ) (other_outpatientservices -> pdc, ) (pharmacy_fills -> pdc, ) (age -> p
> dc, ) (age -> inpatient_hospitalizations, ) (age -> er_visits, ) (age -> pcp_officevisits, ) (age ->
specialist_office_visits, ) (age -> other_outpatientservices, ) (age -> pharmacy_fills, ) (qci_sc
> ore -> pdc, ) (qci_score -> inpatient_hospitalizations, ) (qci_score -> er_visits, ) (qci_score -> pcp_officevisits,
) (qci_score -> specialist_office_visits, ) (qci_score -> other_outpatientservice
> s, ) (qci_score -> pharmacy_fills, ) (engagement_rate -> pdc, ) (engagement_rate -> inpatient_hospitalizations, )
(engagement_rate -> er_visits, ) (engagement_rate -> pcp_officevisits, ) (engagement
> _rate -> specialist_office_visits, ) (engagement_rate -> other_outpatientservices, ) (engagement_rate ->
pharmacy_fills, ) (rewards -> pdc, ) (rewards -> inpatient_hospitalizations, ) (rewards -> er
```

```
> _visits, ) (rewards -> pcp_officevisits, ) (rewards -> specialist_office_visits, ) (rewards ->
other_outpatientservices, ) (rewards -> pharmacy_fills, ), standardized cov( age*qci_score age*engageme
> nt_rate qci_score*engagement_rate engagement_rate*rewards rewards*age rewards*qci_score) nocapslatent

Endogenous variables

Observed:  inpatient_hospitalizations pdc er_visits pcp_officevisits specialist_office_visits other_outpatientservices
pharmacy_fills

Exogenous variables

Observed:  age qci_score engagement_rate rewards

Fitting target model:

Iteration 0:   log likelihood = -1036742.6
Iteration 1:   log likelihood = -1036742.6

Structural equation model                      Number of obs     =     37,359
Estimation method  = ml
Log likelihood     = -1036742.6

----------------------------------------------------------------------------------------------
                             |                 OIM
                Standardized |      Coef.   Std. Err.      z    P>|z|     [95% Conf. Interval]
-----------------------------+----------------------------------------------------------------
Structural                   |
  inpatient_hospitalizations |
                         age |  -.0117148   .0052245    -2.24   0.025    -.0219546   -.0014749
                   qci_score |   .1260713    .005188    24.30   0.000      .115903    .1362397
             engagement_rate |  -.0127361   .0050229    -2.54   0.011    -.0225808   -.0028914
                     rewards |  -.2047775   .0049186   -41.63   0.000    -.2144177   -.1951372
                       _cons |   .4551753   .0212149    21.46   0.000     .4135949    .4967557
  ---------------------------+----------------------------------------------------------------
  pdc                        |
  inpatient_hospitalizations |  -.0059424   .0050333    -1.18   0.238    -.0158075    .0039228
                   er_visits |   .0035852   .0049787     0.72   0.471    -.0061728    .0133433
            pcp_officevisits |  -.0031194   .0068728    -0.45   0.650    -.0165898    .0103509
    specialist_office_visits |   .0008611   .0107513     0.08   0.936     -.020211    .0219332
    other_outpatientservices |  -.0260383   .0102407    -2.54   0.011    -.0461096   -.0059669
```

pharmacy_fills	.3257464	.0047411	68.71	0.000	.3164539	.3350388
age	.1472421	.0049423	29.79	0.000	.1375554	.1569288
qci_score	-.0313731	.004991	-6.29	0.000	-.0411552	-.0215909
engagement_rate	.0086041	.0047539	1.81	0.070	-.0007135	.0179216
rewards	.1363877	.0048956	27.86	0.000	.1267925	.1459829
_cons	.8937658	.0225853	39.57	0.000	.8494993	.9380322
er_visits						
age	-.0370254	.0052662	-7.03	0.000	-.0473469	-.0267039
qci_score	.0198455	.0052685	3.77	0.000	.0095195	.0301715
engagement_rate	-.0192466	.0050652	-3.80	0.000	-.0291743	-.009319
rewards	-.1986002	.0049654	-40.00	0.000	-.2083321	-.1888682
_cons	.6193624	.0212275	29.18	0.000	.5777573	.6609675
pcp_officevisits						
age	.0607266	.0053595	11.33	0.000	.0502222	.071231
qci_score	.0191094	.0053677	3.56	0.000	.0085888	.02963
engagement_rate	.0001796	.0051616	0.03	0.972	-.0099369	.0102961
rewards	.0013861	.0051616	0.27	0.788	-.0087304	.0115026
_cons	.6418804	.0222031	28.91	0.000	.5983632	.6853977
specialist_office_visits						
age	.0570474	.0053642	10.63	0.000	.0465337	.0675611
qci_score	.0020729	.0053723	0.39	0.700	-.0084567	.0126024
engagement_rate	.0002929	.0051651	0.06	0.955	-.0098305	.0104163
rewards	.0017986	.0051651	0.35	0.728	-.0083248	.011922
_cons	.487163	.0221029	22.04	0.000	.4438422	.5304838
other_outpatientservices						
age	.0750126	.0053462	14.03	0.000	.0645342	.085491
qci_score	.0307186	.0053578	5.73	0.000	.0202174	.0412197
engagement_rate	.0003077	.0051534	0.06	0.952	-.0097928	.0104081
rewards	.0021797	.0051534	0.42	0.672	-.0079207	.0122801
_cons	.1747415	.0219364	7.97	0.000	.1317469	.2177361
pharmacy_fills						
age	.1127401	.005295	21.29	0.000	.1023621	.1231181
qci_score	.0614141	.0053171	11.55	0.000	.0509929	.0718354
engagement_rate	.0007853	.0051208	0.15	0.878	-.0092513	.0108219
rewards	.0077803	.0051206	1.52	0.129	-.0022559	.0178166

```
                       _cons |    .877058   .0224515    39.06   0.000     .8330539    .9210621
-----------------------------+----------------------------------------------------------------
                   mean(age)|   3.338398   .0132637   251.69   0.000     3.312401    3.364394
             mean(qci_score)|   1.297835   .0070221   184.82   0.000     1.284072    1.311598
       mean(engagement_rate)|   1.300017   .0070275   184.99   0.000     1.286244    1.313791
               mean(rewards)|   2.000027   .0089612   223.19   0.000     1.982463     2.01759
-----------------------------+----------------------------------------------------------------
var(e.inpatient_hospitaliz~s)|   .9426941   .0023351                      .9381285    .9472819
                  var(e.pdc)|   .8439326   .0036053                      .8368959    .8510286
            var(e.er_visits)|   .9588389   .0020129                      .9549018    .9627922
      var(e.pcp_officevisits)|   .9953069   .0007055                      .9939251    .9966907
var(e.specialist_office_vi~s)|   .9966731   .0005948                      .9955079    .9978397
var(e.other_outpatientserv~s)|   .9921573   .0009092                      .9903769    .9939408
       var(e.pharmacy_fills)|   .9796493   .0014461                      .9768192    .9824877
                    var(age)|          1          .                             .           .
              var(qci_score)|          1          .                             .           .
        var(engagement_rate)|          1          .                             .           .
                var(rewards)|          1          .                             .           .
-----------------------------+----------------------------------------------------------------
         cov(age,qci_score)|   .2750612   .0047823    57.52   0.000     .2656881    .2844343
    cov(age,engagement_rate)|  -.0000374   .0051737    -0.01   0.994    -.0101777    .0101029
            cov(age,rewards)|  -.0008967   .0051737    -0.17   0.862     -.011037    .0092436
              cov(qci_score,|
            engagement_rate)|   4.90e-06   .0051737     0.00   0.999    -.0101354    .0101452
      cov(qci_score,rewards)|   .0001177   .0051737     0.02   0.982    -.0100226     .010258
 cov(engagement_rate,rewards)|   .0003037   .0051737     0.06   0.953    -.0098366     .010444
----------------------------------------------------------------------------------------------
Note: The LR test of model vs. saturated is not reported because the fitted
      model is not full rank.
.
. estat gof, stats(all)

----------------------------------------------------------------------------
Fit statistic        |      Value   Description
---------------------+------------------------------------------------------
Likelihood ratio     |
          chi2_ms(.) |          .   model vs. saturated
            p > chi2 |          .
         chi2_bs(49) |  99660.434   baseline vs. saturated
            p > chi2 |      0.000
```

```
---------------------+------------------------------------------------------
Population error     |
               RMSEA |          .   Root mean squared error of approximation
 90% CI, lower bound |      0.000
         upper bound |          .
              pclose |          .   Probability RMSEA <= 0.05
---------------------+------------------------------------------------------
Information criteria |
                 AIC |  2.074e+06   Akaike's information criterion
                 BIC |  2.074e+06   Bayesian information criterion
---------------------+------------------------------------------------------
Baseline comparison  |
                 CFI |      1.000   Comparative fit index
                 TLI |          .   Tucker-Lewis index
---------------------+------------------------------------------------------
Size of residuals    |
                SRMR |      0.173   Standardized root mean squared residual
                  CD |      0.163   Coefficient of determination
----------------------------------------------------------------------------

. estat teffects, standardized


Direct effects
----------------------------------------------------------------------------------------------
                             |                 OIM
                             |      Coef.   Std. Err.      z    P>|z|              Std. Coef.
-----------------------------+----------------------------------------------------------------
Structural                   |
  inpatient_hospitalizations |
                         age |  -.0002052   .0000915    -2.24   0.025               -.0117148
                   qci_score |   .0289918   .0012015    24.13   0.000                .1260713
             engagement_rate |  -.0000184   7.27e-06    -2.54   0.011               -.0127361
                     rewards |  -.0035559   .0000872   -40.77   0.000               -.2047775
  ---------------------------+----------------------------------------------------------------
  pdc                        |
  inpatient_hospitalizations |  -.0065554   .0055533    -1.18   0.238               -.0059424
                   er_visits |   .0051142   .0071018     0.72   0.471                .0035852
          pcp_officevisits |  -.0003587   .0007903    -0.45   0.650               -.0031194
    specialist_office_visits |   .0000649   .0008099     0.08   0.936                .0008611
```

```
    other_outpatientservices |  -.0015833   .0006233    -2.54   0.011                 -.0260383
              pharmacy_fills |   .0126969    .000196    64.77   0.000                  .3257464
                         age |   .0028454   .0000964    29.53   0.000                  .1472421
                   qci_score |   -.007959   .0012664    -6.28   0.000                 -.0313731
             engagement_rate |   .0000137   7.59e-06     1.81   0.070                  .0086041
                     rewards |   .0026127   .0000944    27.68   0.000                  .1363877
  ---------------------------+---------------------------------------------------------------
  er_visits                  |
                         age |  -.0005016   .0000714    -7.03   0.000                 -.0370254
                   qci_score |   .0035294   .0009371     3.77   0.000                  .0198455
             engagement_rate |  -.0000215   5.67e-06    -3.80   0.000                 -.0192466
                     rewards |  -.0026671    .000068   -39.20   0.000                 -.1986002
  ---------------------------+---------------------------------------------------------------
  pcp_officevisits           |
                         age |   .0102051   .0009022    11.31   0.000                  .0607266
                   qci_score |   .0421582    .011844     3.56   0.000                  .0191094
             engagement_rate |   2.49e-06   .0000717     0.03   0.972                  .0001796
                     rewards |   .0002309   .0008599     0.27   0.788                  .0013861
  ---------------------------+---------------------------------------------------------------
  specialist_office_visits   |
                         age |   .0146336   .0013781    10.62   0.000                  .0570474
                   qci_score |   .0069804   .0180914     0.39   0.700                  .0020729
             engagement_rate |   6.21e-06   .0001095     0.06   0.955                  .0002929
                     rewards |   .0004574   .0013134     0.35   0.728                  .0017986
  ---------------------------+---------------------------------------------------------------
  other_outpatientservices   |
                         age |   .0238391   .0017035    13.99   0.000                  .0750126
                   qci_score |   .1281599   .0223629     5.73   0.000                  .0307186
             engagement_rate |   8.08e-06   .0001353     0.06   0.952                  .0003077
                     rewards |   .0006867   .0016235     0.42   0.672                  .0021797
  ---------------------------+---------------------------------------------------------------
  pharmacy_fills             |
                         age |    .055894   .0026406    21.17   0.000                  .1127401
                   qci_score |   .3997155    .034666    11.53   0.000                  .0614141
             engagement_rate |   .0000322   .0002097     0.15   0.878                  .0007853
                     rewards |   .0038238   .0025167     1.52   0.129                  .0077803
---------------------------------------------------------------------------------------------


Indirect effects
```

	Coef.	OIM Std. Err.	z	P>|z|	Std. Coef.
**Structural**					
inpatient_hospitalizations					
age	0	(no path)			0
qci_score	0	(no path)			0
engagement_rate	0	(no path)			0
rewards	0	(no path)			0
pdc					
inpatient_hospitalizations	0	(no path)			0
er_visits	0	(no path)			0
pcp_officevisits	0	(no path)			0
specialist_office_visits	0	(no path)			0
other_outpatientservices	0	(no path)			0
pharmacy_fills	0	(no path)			0
age	.000668	.0000359	18.60	0.000	.034568
qci_score	.0046855	.0004754	9.86	0.000	.0184697
engagement_rate	4.06e-07	2.68e-06	0.15	0.880	.0002542
rewards	.0000571	.0000406	1.41	0.160	.0029797
er_visits					
age	0	(no path)			0
qci_score	0	(no path)			0
engagement_rate	0	(no path)			0
rewards	0	(no path)			0
pcp_officevisits					
age	0	(no path)			0
qci_score	0	(no path)			0
engagement_rate	0	(no path)			0
rewards	0	(no path)			0
specialist_office_visits					
age	0	(no path)			0
qci_score	0	(no path)			0
engagement_rate	0	(no path)			0
rewards	0	(no path)			0

**other_outpatientservices**					
age	0	(no path)			0
qci_score	0	(no path)			0
engagement_rate	0	(no path)			0
rewards	0	(no path)			0
**pharmacy_fills**					
age	0	(no path)			0
qci_score	0	(no path)			0
engagement_rate	0	(no path)			0
rewards	0	(no path)			0

Total effects

	Coef.	OIM Std. Err.	z	P>|z|	Std. Coef.
**Structural**					
**inpatient_hospitalizations**					
age	-.0002052	.0000915	-2.24	0.025	-.0117148
qci_score	.0289918	.0012015	24.13	0.000	.1260713
engagement_rate	-.0000184	7.27e-06	-2.54	0.011	-.0127361
rewards	-.0035559	.0000872	-40.77	0.000	-.2047775
**pdc**					
inpatient_hospitalizations	-.0065554	.0055533	-1.18	0.238	-.0059424
er_visits	.0051142	.0071018	0.72	0.471	.0035852
pcp_officevisits	-.0003587	.0007903	-0.45	0.650	-.0031194
specialist_office_visits	.0000649	.0008099	0.08	0.936	.0008611
other_outpatientservices	-.0015833	.0006233	-2.54	0.011	-.0260383
pharmacy_fills	.0126969	.000196	64.77	0.000	.3257464
age	.0035134	.0001013	34.69	0.000	.1818102
qci_score	-.0032734	.0013296	-2.46	0.014	-.0129033
engagement_rate	.0000141	8.04e-06	1.76	0.079	.0088582
rewards	.0026698	.0000965	27.66	0.000	.1393674
**er_visits**					

age	-.0005016	.0000714	-7.03	0.000	-.0370254
qci_score	.0035294	.0009371	3.77	0.000	.0198455
engagement_rate	-.0000215	5.67e-06	-3.80	0.000	-.0192466
rewards	-.0026671	.000068	-39.20	0.000	-.1986002
pcp_officevisits					
age	.0102051	.0009022	11.31	0.000	.0607266
qci_score	.0421582	.011844	3.56	0.000	.0191094
engagement_rate	2.49e-06	.0000717	0.03	0.972	.0001796
rewards	.0002309	.0008599	0.27	0.788	.0013861
specialist_office_visits					
age	.0146336	.0013781	10.62	0.000	.0570474
qci_score	.0069804	.0180914	0.39	0.700	.0020729
engagement_rate	6.21e-06	.0001095	0.06	0.955	.0002929
rewards	.0004574	.0013134	0.35	0.728	.0017986
other_outpatientservices					
age	.0238391	.0017035	13.99	0.000	.0750126
qci_score	.1281599	.0223629	5.73	0.000	.0307186
engagement_rate	8.08e-06	.0001353	0.06	0.952	.0003077
rewards	.0006867	.0016235	0.42	0.672	.0021797
pharmacy_fills					
age	.055894	.0026406	21.17	0.000	.1127401
qci_score	.3997155	.034666	11.53	0.000	.0614141
engagement_rate	.0000322	.0002097	0.15	0.878	.0007853
rewards	.0038238	.0025167	1.52	0.129	.0077803

Appendix 3: Sensitivity Analysis Results Presenting Detailed Outputs of Eight Additional Structural Equation Models and Their Estimated Direct, Indirect and Total Effects

#SCENARIO 1: Sensitivity analysis 10% higher covariance (S1)

```
. ssd init age inpatient_hospitalizations er_visits pcp_officevisits specialist_office_visits other_outpatientservices
pharmacy_fills qci_score mpr pdc rewards engagement_rate

Summary statistics data initialized.  Next use, in any order,

    ssd set observations (required)
        It is best to do this first.

    ssd set means (optional)
        Default setting is 0.

    ssd set variances or ssd set sd (optional)
        Use this only if you have set or will set correlations and, even then, this is optional but highly recommended.
Default setting is 1.

    ssd set covariances or ssd set correlations (required)

.
. ssd set observations 37359
  (value set)

    Status:
                     observations:    set
                            means:  unset
                  variances or sd:  unset
        covariances or correlations:  unset (required to be set)

.
. ssd set means 49.64 0.04 0.02 2.18 2.61 2.22 9.95 1.47 0.58 0.59 30 234
  (values set)

    Status:
                     observations:    set
                            means:    set
```

```
                    variances or sd:  unset
        covariances or correlations:  unset (required to be set)

.
. ssd set covariances 221.105233\0.089650 0.067846\-0.094017 0.011868 0.040578\2.451662 0.037385 0.033620
6.244202\3.267802 0.033255 0.022245 6.805591 14.548804\ 5.864529 0.113002 0.075032 7.918511 15
> .837080 22.331055\ 14.209445 0.200045 0.084031 4.385411 7.174931 10.065882 54.346633\ 4.632682 0.036237 0.002199
0.1013640 .076749 0.274862 0.771759 1.282943\ 0.803770 0.000065 -0.001100 0.087459 0.
> 131478 0.182628 1.037027 0.018688 0.129239\ 0.761121 -0.000166 -0.000795 0.047573 0.073374 0.105173 0.708629 0.012082
0.062483 0.082140\-0.2 -0.8 -0.6 0.05 0.1 0.15 0.85 0.002 0.77 0.66 225\-0.1 -.6
>  -0.7 0.08 0.2 0.26 1.04 0.001 0.55 0.506 0.82 32400
  (values set)

    Status:
                      observations:    set
                             means:    set
                   variances or sd:    set
       covariances or correlations:    set
```

. MODEL 9

```
. sem (inpatient_hospitalizations -> mpr, ) (er_visits -> mpr, ) (pcp_officevisits -> mpr, ) (specialist_office_visits
-> mpr, ) (other_outpatientservices -> mpr, ) (pharmacy_fills -> mpr, ) (age -> m
> pr, ) (age -> inpatient_hospitalizations, ) (age -> er_visits, ) (age -> pcp_officevisits, ) (age ->
specialist_office_visits, ) (age -> other_outpatientservices, ) (age -> pharmacy_fills, ) (qci_sc
> ore -> mpr, ) (qci_score -> inpatient_hospitalizations, ) (qci_score -> er_visits, ) (qci_score -> pcp_officevisits,
) (qci_score -> specialist_office_visits, ) (qci_score -> other_outpatientservice
> s, ) (qci_score -> pharmacy_fills, ) (engagement_rate -> mpr, ) (engagement_rate -> inpatient_hospitalizations, )
(engagement_rate -> er_visits, ) (engagement_rate -> pcp_officevisits, ) (engagement
> _rate -> specialist_office_visits, ) (engagement_rate -> other_outpatientservices, ) (engagement_rate ->
pharmacy_fills, ) (rewards -> mpr, ) (rewards -> inpatient_hospitalizations, ) (rewards -> er
> _visits, ) (rewards -> pcp_officevisits, ) (rewards -> specialist_office_visits, ) (rewards ->
other_outpatientservices, ) (rewards -> pharmacy_fills, ), standardized cov( age*qci_score age*engageme
> nt_rate qci_score*engagement_rate engagement_rate*rewards rewards*age rewards*qci_score) nocapslatent

Endogenous variables
```

```
Observed:  inpatient_hospitalizations mpr er_visits pcp_officevisits specialist_office_visits other_outpatientservices
pharmacy_fills

Exogenous variables

Observed:  age qci_score engagement_rate rewards

Fitting target model:

Iteration 0:   log likelihood = -1044494.8
Iteration 1:   log likelihood = -1044494.8

Structural equation model                      Number of obs     =     37,359
Estimation method  = ml
Log likelihood     = -1044494.8

-------------------------------------------------------------------------------------------------
                             |                 OIM
                Standardized |      Coef.   Std. Err.      z    P>|z|     [95% Conf. Interval]
-----------------------------+-------------------------------------------------------------------
Structural                   |
  inpatient_hospitalizations |
                         age |  -.0117148   .0052245    -2.24   0.025    -.0219546   -.0014749
                   qci_score |   .1260713    .005188    24.30   0.000      .115903    .1362397
             engagement_rate |  -.0127361   .0050229    -2.54   0.011    -.0225808   -.0028914
                     rewards |  -.2047775   .0049186   -41.63   0.000    -.2144177   -.1951372
                       _cons |   .4551753   .0212149    21.46   0.000     .4135949    .4967557
  ---------------------------+-------------------------------------------------------------------
  mpr                        |
  inpatient_hospitalizations |  -.0095253   .0049433    -1.93   0.054    -.0192141    .0001635
                   er_visits |  -.0030873   .0048902    -0.63   0.528    -.0126719    .0064973
           pcp_officevisits |   .0134152   .0067513     1.99   0.047     .0001829    .0266476
    specialist_office_visits |  -.0003967   .0105607    -0.04   0.970    -.0210952    .0203018
    other_outpatientservices |  -.0185354   .0100627    -1.84   0.065    -.0382579    .0011871
              pharmacy_fills |    .380903   .0045536    83.65   0.000     .3719781    .3898278
                         age |   .1063776   .0048757    21.82   0.000     .0968214    .1159339
                   qci_score |  -.0169726   .0049033    -3.46   0.001    -.0265829   -.0073623
             engagement_rate |   .0079735   .0046696     1.71   0.088    -.0011788    .0171257
                     rewards |   .1372191   .0048113    28.52   0.000     .1277891    .1466491
                       _cons |    .478165   .0214995    22.24   0.000     .4360268    .5203032
```

er_visits						
age	-.0370254	.0052662	-7.03	0.000	-.0473469	-.0267039
qci_score	.0198455	.0052685	3.77	0.000	.0095195	.0301715
engagement_rate	-.0192466	.0050652	-3.80	0.000	-.0291743	-.009319
rewards	-.1986002	.0049654	-40.00	0.000	-.2083321	-.1888682
_cons	.6193624	.0212275	29.18	0.000	.5777573	.6609675
pcp_officevisits						
age	.0607266	.0053595	11.33	0.000	.0502222	.071231
qci_score	.0191094	.0053677	3.56	0.000	.0085888	.02963
engagement_rate	.0001796	.0051616	0.03	0.972	-.0099369	.0102961
rewards	.0013861	.0051616	0.27	0.788	-.0087304	.0115026
_cons	.6418804	.0222031	28.91	0.000	.5983632	.6853977
specialist_office_visits						
age	.0570474	.0053642	10.63	0.000	.0465337	.0675611
qci_score	.0020729	.0053723	0.39	0.700	-.0084567	.0126024
engagement_rate	.0002929	.0051651	0.06	0.955	-.0098305	.0104163
rewards	.0017986	.0051651	0.35	0.728	-.0083248	.011922
_cons	.487163	.0221029	22.04	0.000	.4438422	.5304838
other_outpatientservices						
age	.0750126	.0053462	14.03	0.000	.0645342	.085491
qci_score	.0307186	.0053578	5.73	0.000	.0202174	.0412197
engagement_rate	.0003077	.0051534	0.06	0.952	-.0097928	.0104081
rewards	.0021797	.0051534	0.42	0.672	-.0079207	.0122801
_cons	.1747415	.0219364	7.97	0.000	.1317469	.2177361
pharmacy_fills						
age	.1127401	.005295	21.29	0.000	.1023621	.1231181
qci_score	.0614141	.0053171	11.55	0.000	.0509929	.0718354
engagement_rate	.0007853	.0051208	0.15	0.878	-.0092513	.0108219
rewards	.0077803	.0051206	1.52	0.129	-.0022559	.0178166
_cons	.877058	.0224515	39.06	0.000	.8330539	.9210621
mean(age)	3.338398	.0132637	251.69	0.000	3.312401	3.364394
mean(qci_score)	1.297835	.0070221	184.82	0.000	1.284072	1.311598
mean(engagement_rate)	1.300017	.0070275	184.99	0.000	1.286244	1.313791
mean(rewards)	2.000027	.0089612	223.19	0.000	1.982463	2.01759

```
-----------------------------+----------------------------------------------------------------
var(e.inpatient_hospitaliz~s)|   .9426941   .0023351                           .9381285    .9472819
                 var(e.mpr)|   .8142406   .0037738                           .8068776    .8216708
           var(e.er_visits)|   .9588389   .0020129                           .9549018    .9627922
    var(e.pcp_officevisits)|   .9953069   .0007055                           .9939251    .9966907
var(e.specialist_office_vi~s)|   .9966731   .0005948                           .9955079    .9978397
var(e.other_outpatientserv~s)|   .9921573   .0009092                           .9903769    .9939408
      var(e.pharmacy_fills)|   .9796493   .0014461                           .9768192    .9824877
                   var(age)|          1          .                                  .           .
             var(qci_score)|          1          .                                  .           .
       var(engagement_rate)|          1          .                                  .           .
               var(rewards)|          1          .                                  .           .
-----------------------------+----------------------------------------------------------------
         cov(age,qci_score)|   .2750612   .0047823    57.52   0.000     .2656881    .2844343
   cov(age,engagement_rate)|  -.0000374   .0051737    -0.01   0.994    -.0101777    .0101029
           cov(age,rewards)|  -.0008967   .0051737    -0.17   0.862     -.011037    .0092436
             cov(qci_score,|
           engagement_rate)|   4.90e-06   .0051737     0.00   0.999    -.0101354    .0101452
     cov(qci_score,rewards)|   .0001177   .0051737     0.02   0.982    -.0100226     .010258
 cov(engagement_rate,rewards)|   .0003037   .0051737     0.06   0.953    -.0098366     .010444
----------------------------------------------------------------------------------------------
Note: The LR test of model vs. saturated is not reported because the fitted
      model is not full rank.

.
. estat gof, stats(all)

----------------------------------------------------------------------------
Fit statistic        |      Value   Description
---------------------+------------------------------------------------------
Likelihood ratio     |
          chi2_ms(.) |          .   model vs. saturated
            p > chi2 |          .
         chi2_bs(49) | 101088.450   baseline vs. saturated
            p > chi2 |      0.000
---------------------+------------------------------------------------------
Population error     |
               RMSEA |          .   Root mean squared error of approximation
 90% CI, lower bound |      0.000
         upper bound |          .
```

```
             pclose |          .   Probability RMSEA <= 0.05
---------------------+------------------------------------------------------
Information criteria |
                 AIC |  2.089e+06   Akaike's information criterion
                 BIC |  2.090e+06   Bayesian information criterion
---------------------+------------------------------------------------------
Baseline comparison  |
                 CFI |      1.000   Comparative fit index
                 TLI |          .   Tucker-Lewis index
---------------------+------------------------------------------------------
Size of residuals    |
                SRMR |      0.174   Standardized root mean squared residual
                  CD |      0.155   Coefficient of determination
----------------------------------------------------------------------------

.
. estat teffects, standardized


Direct effects
----------------------------------------------------------------------------------------------
                             |                 OIM
                             |      Coef.   Std. Err.      z    P>|z|                Std. Coef.
-----------------------------+----------------------------------------------------------------
Structural                   |
  inpatient_hospitalizations |
                         age |  -.0002052   .0000915    -2.24   0.025                 -.0117148
                   qci_score |   .0289918   .0012015    24.13   0.000                  .1260713
             engagement_rate |  -.0000184   7.27e-06    -2.54   0.011                 -.0127361
                     rewards |  -.0035559   .0000872   -40.77   0.000                 -.2047775
  ---------------------------+----------------------------------------------------------------
  mpr                        |
  inpatient_hospitalizations |  -.0131648   .0068339    -1.93   0.054                 -.0095253
                   er_visits |  -.0055173   .0087396    -0.63   0.528                 -.0030873
            pcp_officevisits |   .0019327   .0009726     1.99   0.047                  .0134152
    specialist_office_visits |  -.0000374   .0009967    -0.04   0.970                 -.0003967
    other_outpatientservices |   -.001412   .0007671    -1.84   0.066                 -.0185354
              pharmacy_fills |   .0186006   .0002412    77.11   0.000                   .380903
                         age |   .0025754   .0001186    21.72   0.000                  .1063776
                   qci_score |  -.0053944   .0015584    -3.46   0.001                 -.0169726
```

```
          engagement_rate |   .0000159   9.34e-06     1.71   0.088                 .0079735
                  rewards |   .0032932   .0001162    28.35   0.000                 .1372191
  ------------------------+----------------------------------------------------------------
  er_visits               |
                      age |  -.0005016   .0000714    -7.03   0.000                -.0370254
                qci_score |   .0035294   .0009371     3.77   0.000                 .0198455
          engagement_rate |  -.0000215   5.67e-06    -3.80   0.000                -.0192466
                  rewards |  -.0026671    .000068   -39.20   0.000                -.1986002
  ------------------------+----------------------------------------------------------------
  pcp_officevisits        |
                      age |   .0102051   .0009022    11.31   0.000                 .0607266
                qci_score |   .0421582    .011844     3.56   0.000                 .0191094
          engagement_rate |   2.49e-06   .0000717     0.03   0.972                 .0001796
                  rewards |   .0002309   .0008599     0.27   0.788                 .0013861
  ------------------------+----------------------------------------------------------------
  specialist_office_visits   |
                      age |   .0146336   .0013781    10.62   0.000                 .0570474
                qci_score |   .0069804   .0180914     0.39   0.700                 .0020729
          engagement_rate |   6.21e-06   .0001095     0.06   0.955                 .0002929
                  rewards |   .0004574   .0013134     0.35   0.728                 .0017986
  ------------------------+----------------------------------------------------------------
  other_outpatientservices   |
                      age |   .0238391   .0017035    13.99   0.000                 .0750126
                qci_score |   .1281599   .0223629     5.73   0.000                 .0307186
          engagement_rate |   8.08e-06   .0001353     0.06   0.952                 .0003077
                  rewards |   .0006867   .0016235     0.42   0.672                 .0021797
  ------------------------+----------------------------------------------------------------
  pharmacy_fills          |
                      age |    .055894   .0026406    21.17   0.000                 .1127401
                qci_score |   .3997155    .034666    11.53   0.000                 .0614141
          engagement_rate |   .0000322   .0002097     0.15   0.878                 .0007853
                  rewards |   .0038238   .0025167     1.52   0.129                 .0077803
--------------------------------------------------------------------------------------------


Indirect effects
--------------------------------------------------------------------------------------------
                          |                OIM
                          |      Coef.   Std. Err.      z    P>|z|              Std. Coef.
--------------------------+-----------------------------------------------------------------
```

```
Structural                    |
  inpatient_hospitalizations |
                         age |          0  (no path)                                            0
                   qci_score |          0  (no path)                                            0
             engagement_rate |          0  (no path)                                            0
                     rewards |          0  (no path)                                            0
  ---------------------------+----------------------------------------------------------------
  mpr                        |
  inpatient_hospitalizations |          0  (no path)                                            0
                   er_visits |          0  (no path)                                            0
            pcp_officevisits |          0  (no path)                                            0
    specialist_office_visits |          0  (no path)                                            0
    other_outpatientservices |          0  (no path)                                            0
              pharmacy_fills |          0  (no path)                                            0
                         age |   .0010306   .0000516    19.98   0.000                    .0425706
                   qci_score |   .0069341   .0006815    10.17   0.000                    .0218168
             engagement_rate |   9.53e-07   3.91e-06     0.24   0.808                    .0004764
                     rewards |   .0001321    .000056     2.36   0.018                    .0055047
  ---------------------------+----------------------------------------------------------------
  er_visits                  |
                         age |          0  (no path)                                            0
                   qci_score |          0  (no path)                                            0
             engagement_rate |          0  (no path)                                            0
                     rewards |          0  (no path)                                            0
  ---------------------------+----------------------------------------------------------------
  pcp_officevisits           |
                         age |          0  (no path)                                            0
                   qci_score |          0  (no path)                                            0
             engagement_rate |          0  (no path)                                            0
                     rewards |          0  (no path)                                            0
  ---------------------------+----------------------------------------------------------------
  specialist_office_visits   |
                         age |          0  (no path)                                            0
                   qci_score |          0  (no path)                                            0
             engagement_rate |          0  (no path)                                            0
                     rewards |          0  (no path)                                            0
  ---------------------------+----------------------------------------------------------------
  other_outpatientservices   |
                         age |          0  (no path)                                            0
                   qci_score |          0  (no path)                                            0
```

```
            engagement_rate |          0  (no path)                                             0
                    rewards |          0  (no path)                                             0
  --------------------------+----------------------------------------------------------------
  pharmacy_fills            |
                        age |          0  (no path)                                             0
                  qci_score |          0  (no path)                                             0
            engagement_rate |          0  (no path)                                             0
                    rewards |          0  (no path)                                             0
-----------------------------------------------------------------------------------------------


Total effects
-----------------------------------------------------------------------------------------------
                            |                 OIM
                            |      Coef.   Std. Err.      z    P>|z|               Std. Coef.
----------------------------+------------------------------------------------------------------
Structural                  |
  inpatient_hospitalizations |
                        age |  -.0002052   .0000915    -2.24   0.025                -.0117148
                  qci_score |   .0289918   .0012015    24.13   0.000                 .1260713
            engagement_rate |  -.0000184   7.27e-06    -2.54   0.011                -.0127361
                    rewards |  -.0035559   .0000872   -40.77   0.000                -.2047775
  --------------------------+----------------------------------------------------------------
  mpr                       |
  inpatient_hospitalizations |  -.0131648   .0068339    -1.93   0.054                -.0095253
                  er_visits |  -.0055173   .0087396    -0.63   0.528                -.0030873
           pcp_officevisits |   .0019327   .0009726     1.99   0.047                 .0134152
    specialist_office_visits |  -.0000374   .0009967    -0.04   0.970                -.0003967
    other_outpatientservices |   -.001412   .0007671    -1.84   0.066                -.0185354
             pharmacy_fills |   .0186006   .0002412    77.11   0.000                  .380903
                        age |   .0036061   .0001275    28.29   0.000                 .1489482
                  qci_score |   .0015397   .0016732     0.92   0.357                 .0048443
            engagement_rate |   .0000169   .0000101     1.67   0.095                 .0084499
                    rewards |   .0034254   .0001215    28.20   0.000                 .1427238
  --------------------------+----------------------------------------------------------------
  er_visits                 |
                        age |  -.0005016   .0000714    -7.03   0.000                -.0370254
                  qci_score |   .0035294   .0009371     3.77   0.000                 .0198455
            engagement_rate |  -.0000215   5.67e-06    -3.80   0.000                -.0192466
                    rewards |  -.0026671    .000068   -39.20   0.000                -.1986002
```

```
  --------------------------+----------------------------------------------------------------
  pcp_officevisits          |
                        age |   .0102051   .0009022    11.31   0.000                 .0607266
                  qci_score |   .0421582    .011844     3.56   0.000                 .0191094
            engagement_rate |   2.49e-06   .0000717     0.03   0.972                 .0001796
                    rewards |   .0002309   .0008599     0.27   0.788                 .0013861
  --------------------------+----------------------------------------------------------------
  specialist_office_visits  |
                        age |   .0146336   .0013781    10.62   0.000                 .0570474
                  qci_score |   .0069804   .0180914     0.39   0.700                 .0020729
            engagement_rate |   6.21e-06   .0001095     0.06   0.955                 .0002929
                    rewards |   .0004574   .0013134     0.35   0.728                 .0017986
  --------------------------+----------------------------------------------------------------
  other_outpatientservices  |
                        age |   .0238391   .0017035    13.99   0.000                 .0750126
                  qci_score |   .1281599   .0223629     5.73   0.000                 .0307186
            engagement_rate |   8.08e-06   .0001353     0.06   0.952                 .0003077
                    rewards |   .0006867   .0016235     0.42   0.672                 .0021797
  --------------------------+----------------------------------------------------------------
  pharmacy_fills            |
                        age |    .055894   .0026406    21.17   0.000                 .1127401
                  qci_score |   .3997155    .034666    11.53   0.000                 .0614141
            engagement_rate |   .0000322   .0002097     0.15   0.878                 .0007853
                    rewards |   .0038238   .0025167     1.52   0.129                 .0077803
--------------------------------------------------------------------------------------------
```

.

MODEL 10

```
. sem (inpatient_hospitalizations -> pdc, ) (er_visits -> pdc, ) (pcp_officevisits -> pdc, ) (specialist_office_visits
-> pdc, ) (other_outpatientservices -> pdc, ) (pharmacy_fills -> pdc, ) (age -> p
> dc, ) (age -> inpatient_hospitalizations, ) (age -> er_visits, ) (age -> pcp_officevisits, ) (age ->
specialist_office_visits, ) (age -> other_outpatientservices, ) (age -> pharmacy_fills, ) (qci_sc
> ore -> pdc, ) (qci_score -> inpatient_hospitalizations, ) (qci_score -> er_visits, ) (qci_score -> pcp_officevisits,
) (qci_score -> specialist_office_visits, ) (qci_score -> other_outpatientservice
> s, ) (qci_score -> pharmacy_fills, ) (engagement_rate -> pdc, ) (engagement_rate -> inpatient_hospitalizations, )
(engagement_rate -> er_visits, ) (engagement_rate -> pcp_officevisits, ) (engagement
```

```
> _rate -> specialist_office_visits, ) (engagement_rate -> other_outpatientservices, ) (engagement_rate ->
pharmacy_fills, ) (rewards -> pdc, ) (rewards -> inpatient_hospitalizations, ) (rewards -> er
> _visits, ) (rewards -> pcp_officevisits, ) (rewards -> specialist_office_visits, ) (rewards ->
other_outpatientservices, ) (rewards -> pharmacy_fills, ), standardized cov( age*qci_score age*engageme
> nt_rate qci_score*engagement_rate engagement_rate*rewards rewards*age rewards*qci_score) nocapslatent

Endogenous variables

Observed:  inpatient_hospitalizations pdc er_visits pcp_officevisits specialist_office_visits other_outpatientservices
pharmacy_fills

Exogenous variables

Observed:  age qci_score engagement_rate rewards

Fitting target model:

Iteration 0:   log likelihood = -1036653.3
Iteration 1:   log likelihood = -1036653.3

Structural equation model                       Number of obs     =     37,359
Estimation method  = ml
Log likelihood     = -1036653.3

--------------------------------------------------------------------------------------------
                             |                 OIM
                Standardized |      Coef.   Std. Err.      z    P>|z|     [95% Conf. Interval]
-----------------------------+--------------------------------------------------------------
Structural                   |
  inpatient_hospitalizations |
                         age |  -.0117148   .0052245    -2.24   0.025    -.0219546   -.0014749
                   qci_score |   .1260713    .005188    24.30   0.000      .115903    .1362397
             engagement_rate |  -.0127361   .0050229    -2.54   0.011    -.0225808   -.0028914
                     rewards |  -.2047775   .0049186   -41.63   0.000    -.2144177   -.1951372
                       _cons |   .4551753   .0212149    21.46   0.000     .4135949    .4967557
  ---------------------------+--------------------------------------------------------------
  pdc                        |
  inpatient_hospitalizations |  -.0032664    .005022    -0.65   0.515    -.0131093    .0065764
                   er_visits |   .0060824   .0049671     1.22   0.221    -.0036529    .0158178
            pcp_officevisits |  -.0033853   .0068568    -0.49   0.622    -.0168243    .0100538
```

specialist_office_visits	.0019877	.010726	0.19	0.853	-.019035	.0230103
other_outpatientservices	-.0272061	.0102159	-2.66	0.008	-.0472289	-.0071834
pharmacy_fills	.3253462	.0047322	68.75	0.000	.3160713	.334621
age	.1474767	.0049309	29.91	0.000	.1378124	.157141
qci_score	-.0317031	.0049794	-6.37	0.000	-.0414627	-.0219436
engagement_rate	.0095724	.0047429	2.02	0.044	.0002765	.0188682
rewards	.1513662	.0048764	31.04	0.000	.1418086	.1609238
_cons	.8622232	.0225095	38.30	0.000	.8181055	.906341
er_visits						
age	-.0370254	.0052662	-7.03	0.000	-.0473469	-.0267039
qci_score	.0198455	.0052685	3.77	0.000	.0095195	.0301715
engagement_rate	-.0192466	.0050652	-3.80	0.000	-.0291743	-.009319
rewards	-.1986002	.0049654	-40.00	0.000	-.2083321	-.1888682
_cons	.6193624	.0212275	29.18	0.000	.5777573	.6609675
pcp_officevisits						
age	.0607266	.0053595	11.33	0.000	.0502222	.071231
qci_score	.0191094	.0053677	3.56	0.000	.0085888	.02963
engagement_rate	.0001796	.0051616	0.03	0.972	-.0099369	.0102961
rewards	.0013861	.0051616	0.27	0.788	-.0087304	.0115026
_cons	.6418804	.0222031	28.91	0.000	.5983632	.6853977
specialist_office_visits						
age	.0570474	.0053642	10.63	0.000	.0465337	.0675611
qci_score	.0020729	.0053723	0.39	0.700	-.0084567	.0126024
engagement_rate	.0002929	.0051651	0.06	0.955	-.0098305	.0104163
rewards	.0017986	.0051651	0.35	0.728	-.0083248	.011922
_cons	.487163	.0221029	22.04	0.000	.4438422	.5304838
other_outpatientservices						
age	.0750126	.0053462	14.03	0.000	.0645342	.085491
qci_score	.0307186	.0053578	5.73	0.000	.0202174	.0412197
engagement_rate	.0003077	.0051534	0.06	0.952	-.0097928	.0104081
rewards	.0021797	.0051534	0.42	0.672	-.0079207	.0122801
_cons	.1747415	.0219364	7.97	0.000	.1317469	.2177361
pharmacy_fills						
age	.1127401	.005295	21.29	0.000	.1023621	.1231181
qci_score	.0614141	.0053171	11.55	0.000	.0509929	.0718354

```
             engagement_rate |   .0007853   .0051208     0.15   0.878    -.0092513    .0108219
                     rewards |   .0077803   .0051206     1.52   0.129    -.0022559    .0178166
                       _cons |    .877058   .0224515    39.06   0.000     .8330539    .9210621
-----------------------------+----------------------------------------------------------------
                   mean(age)|   3.338398   .0132637   251.69   0.000     3.312401    3.364394
             mean(qci_score)|   1.297835   .0070221   184.82   0.000     1.284072    1.311598
       mean(engagement_rate)|   1.300017   .0070275   184.99   0.000     1.286244    1.313791
               mean(rewards)|   2.000027   .0089612   223.19   0.000     1.982463     2.01759
-----------------------------+----------------------------------------------------------------
var(e.inpatient_hospitaliz~s)|   .9426941   .0023351                      .9381285    .9472819
                  var(e.pdc)|   .8400235   .0036334                      .8329322    .8471751
            var(e.er_visits)|   .9588389   .0020129                      .9549018    .9627922
     var(e.pcp_officevisits)|   .9953069   .0007055                      .9939251    .9966907
var(e.specialist_office_vi~s)|   .9966731   .0005948                      .9955079    .9978397
var(e.other_outpatientserv~s)|   .9921573   .0009092                      .9903769    .9939408
       var(e.pharmacy_fills)|   .9796493   .0014461                      .9768192    .9824877
                    var(age)|          1          .                             .           .
              var(qci_score)|          1          .                             .           .
        var(engagement_rate)|          1          .                             .           .
                var(rewards)|          1          .                             .           .
-----------------------------+----------------------------------------------------------------
          cov(age,qci_score)|   .2750612   .0047823    57.52   0.000     .2656881    .2844343
    cov(age,engagement_rate)|  -.0000374   .0051737    -0.01   0.994    -.0101777    .0101029
            cov(age,rewards)|  -.0008967   .0051737    -0.17   0.862     -.011037    .0092436
              cov(qci_score,|
             engagement_rate)|   4.90e-06   .0051737     0.00   0.999    -.0101354    .0101452
      cov(qci_score,rewards)|   .0001177   .0051737     0.02   0.982    -.0100226     .010258
 cov(engagement_rate,rewards)|   .0003037   .0051737     0.06   0.953    -.0098366     .010444
----------------------------------------------------------------------------------------------
Note: The LR test of model vs. saturated is not reported because the fitted
      model is not full rank.

.
. estat gof, stats(all)

----------------------------------------------------------------------------
Fit statistic        |      Value   Description
---------------------+------------------------------------------------------
Likelihood ratio     |
          chi2_ms(.) |          .   model vs. saturated
```

```
           p > chi2 |          .
        chi2_bs(49) |  99838.892   baseline vs. saturated
           p > chi2 |      0.000
--------------------+-------------------------------------------------
Population error    |
              RMSEA |          .   Root mean squared error of approximation
 90% CI, lower bound |      0.000
        upper bound |          .
             pclose |          .   Probability RMSEA <= 0.05
--------------------+-------------------------------------------------
Information criteria |
                AIC |  2.073e+06   Akaike's information criterion
                BIC |  2.074e+06   Bayesian information criterion
--------------------+-------------------------------------------------
Baseline comparison  |
                CFI |      1.000   Comparative fit index
                TLI |          .   Tucker-Lewis index
--------------------+-------------------------------------------------
Size of residuals    |
               SRMR |      0.173   Standardized root mean squared residual
                 CD |      0.167   Coefficient of determination
----------------------------------------------------------------------------

.
. estat teffects, standardized


Direct effects
--------------------------------------------------------------------------------------------
                            |                OIM
                            |      Coef.   Std. Err.      z    P>|z|               Std. Coef.
----------------------------+---------------------------------------------------------------
Structural                  |
  inpatient_hospitalizations |
                        age |  -.0002052   .0000915    -2.24   0.025               -.0117148
                  qci_score |   .0289918   .0012015    24.13   0.000                .1260713
            engagement_rate |  -.0000184   7.27e-06    -2.54   0.011               -.0127361
                    rewards |  -.0035559   .0000872   -40.77   0.000               -.2047775
  --------------------------+---------------------------------------------------------------
  pdc                       |
```

inpatient_hospitalizations	-.0036032	.00554	-0.65	0.515	-.0032664
er_visits	.0086757	.0070849	1.22	0.221	.0060824
pcp_officevisits	-.0003892	.0007885	-0.49	0.622	-.0033853
specialist_office_visits	.0001497	.000808	0.19	0.853	.0019877
other_outpatientservices	-.0016542	.0006219	-2.66	0.008	-.0272061
pharmacy_fills	.0126804	.0001956	64.84	0.000	.3253462
age	.0028497	.0000961	29.65	0.000	.1474767
qci_score	-.0080422	.0012634	-6.37	0.000	-.0317031
engagement_rate	.0000153	7.57e-06	2.02	0.044	.0095724
rewards	.0028994	.0000942	30.79	0.000	.1513662
er_visits					
age	-.0005016	.0000714	-7.03	0.000	-.0370254
qci_score	.0035294	.0009371	3.77	0.000	.0198455
engagement_rate	-.0000215	5.67e-06	-3.80	0.000	-.0192466
rewards	-.0026671	.000068	-39.20	0.000	-.1986002
pcp_officevisits					
age	.0102051	.0009022	11.31	0.000	.0607266
qci_score	.0421582	.011844	3.56	0.000	.0191094
engagement_rate	2.49e-06	.0000717	0.03	0.972	.0001796
rewards	.0002309	.0008599	0.27	0.788	.0013861
specialist_office_visits					
age	.0146336	.0013781	10.62	0.000	.0570474
qci_score	.0069804	.0180914	0.39	0.700	.0020729
engagement_rate	6.21e-06	.0001095	0.06	0.955	.0002929
rewards	.0004574	.0013134	0.35	0.728	.0017986
other_outpatientservices					
age	.0238391	.0017035	13.99	0.000	.0750126
qci_score	.1281599	.0223629	5.73	0.000	.0307186
engagement_rate	8.08e-06	.0001353	0.06	0.952	.0003077
rewards	.0006867	.0016235	0.42	0.672	.0021797
pharmacy_fills					
age	.055894	.0026406	21.17	0.000	.1127401
qci_score	.3997155	.034666	11.53	0.000	.0614141
engagement_rate	.0000322	.0002097	0.15	0.878	.0007853
rewards	.0038238	.0025167	1.52	0.129	.0077803

```
-----------------------------------------------------------------------------------------------


Indirect effects
-----------------------------------------------------------------------------------------------
                             |                 OIM
                             |      Coef.   Std. Err.      z    P>|z|               Std. Coef.
-----------------------------+-----------------------------------------------------------------
Structural                   |
  inpatient_hospitalizations |
                         age |          0  (no path)                                         0
                   qci_score |          0  (no path)                                         0
             engagement_rate |          0  (no path)                                         0
                     rewards |          0  (no path)                                         0
  ---------------------------+-----------------------------------------------------------------
  pdc                        |
  inpatient_hospitalizations |          0  (no path)                                         0
                   er_visits |          0  (no path)                                         0
            pcp_officevisits |          0  (no path)                                         0
    specialist_office_visits |          0  (no path)                                         0
    other_outpatientservices |          0  (no path)                                         0
              pharmacy_fills |          0  (no path)                                         0
                         age |   .0006639   .0000359    18.51   0.000                 .0343596
                   qci_score |   .0047674   .0004749    10.04   0.000                 .0187935
             engagement_rate |   2.74e-07   2.67e-06     0.10   0.918                 .0001716
                     rewards |    .000037   .0000405     0.91   0.361                 .0019318
  ---------------------------+-----------------------------------------------------------------
  er_visits                  |
                         age |          0  (no path)                                         0
                   qci_score |          0  (no path)                                         0
             engagement_rate |          0  (no path)                                         0
                     rewards |          0  (no path)                                         0
  ---------------------------+-----------------------------------------------------------------
  pcp_officevisits           |
                         age |          0  (no path)                                         0
                   qci_score |          0  (no path)                                         0
             engagement_rate |          0  (no path)                                         0
                     rewards |          0  (no path)                                         0
  ---------------------------+-----------------------------------------------------------------
  specialist_office_visits   |
```

```
                     age |          0  (no path)                                          0
               qci_score |          0  (no path)                                          0
         engagement_rate |          0  (no path)                                          0
                 rewards |          0  (no path)                                          0
  -----------------------+----------------------------------------------------------------
  other_outpatientservices   |
                     age |          0  (no path)                                          0
               qci_score |          0  (no path)                                          0
         engagement_rate |          0  (no path)                                          0
                 rewards |          0  (no path)                                          0
  -----------------------+----------------------------------------------------------------
  pharmacy_fills         |
                     age |          0  (no path)                                          0
               qci_score |          0  (no path)                                          0
         engagement_rate |          0  (no path)                                          0
                 rewards |          0  (no path)                                          0
------------------------------------------------------------------------------------------


Total effects
------------------------------------------------------------------------------------------
                             |                 OIM
                             |      Coef.   Std. Err.      z    P>|z|              Std. Coef.
-----------------------------+------------------------------------------------------------
Structural                   |
  inpatient_hospitalizations |
                         age |  -.0002052   .0000915    -2.24   0.025               -.0117148
                   qci_score |   .0289918   .0012015    24.13   0.000                .1260713
             engagement_rate |  -.0000184   7.27e-06    -2.54   0.011               -.0127361
                     rewards |  -.0035559   .0000872   -40.77   0.000               -.2047775
  ---------------------------+------------------------------------------------------------
  pdc                        |
  inpatient_hospitalizations |  -.0036032     .00554    -0.65   0.515               -.0032664
                   er_visits |   .0086757   .0070849     1.22   0.221                .0060824
           pcp_officevisits |  -.0003892   .0007885    -0.49   0.622               -.0033853
    specialist_office_visits |   .0001497    .000808     0.19   0.853                .0019877
    other_outpatientservices |  -.0016542   .0006219    -2.66   0.008               -.0272061
              pharmacy_fills |   .0126804   .0001956    64.84   0.000                .3253462
                         age |   .0035136   .0001011    34.77   0.000                .1818364
                   qci_score |  -.0032748   .0013267    -2.47   0.014               -.0129097
```

```
           engagement_rate |   .0000156   8.03e-06     1.94   0.053                        .009744
                   rewards |   .0029364   .0000963    30.49   0.000                        .153298
---------------------------+----------------------------------------------------------------
  er_visits                |
                       age |  -.0005016   .0000714    -7.03   0.000                      -.0370254
                 qci_score |   .0035294   .0009371     3.77   0.000                       .0198455
           engagement_rate |  -.0000215   5.67e-06    -3.80   0.000                      -.0192466
                   rewards |  -.0026671    .000068   -39.20   0.000                      -.1986002
---------------------------+----------------------------------------------------------------
  pcp_officevisits         |
                       age |   .0102051   .0009022    11.31   0.000                       .0607266
                 qci_score |   .0421582    .011844     3.56   0.000                       .0191094
           engagement_rate |   2.49e-06   .0000717     0.03   0.972                       .0001796
                   rewards |   .0002309   .0008599     0.27   0.788                       .0013861
---------------------------+----------------------------------------------------------------
  specialist_office_visits |
                       age |   .0146336   .0013781    10.62   0.000                       .0570474
                 qci_score |   .0069804   .0180914     0.39   0.700                       .0020729
           engagement_rate |   6.21e-06   .0001095     0.06   0.955                       .0002929
                   rewards |   .0004574   .0013134     0.35   0.728                       .0017986
---------------------------+----------------------------------------------------------------
  other_outpatientservices |
                       age |   .0238391   .0017035    13.99   0.000                       .0750126
                 qci_score |   .1281599   .0223629     5.73   0.000                       .0307186
           engagement_rate |   8.08e-06   .0001353     0.06   0.952                       .0003077
                   rewards |   .0006867   .0016235     0.42   0.672                       .0021797
---------------------------+----------------------------------------------------------------
  pharmacy_fills           |
                       age |    .055894   .0026406    21.17   0.000                       .1127401
                 qci_score |   .3997155    .034666    11.53   0.000                       .0614141
           engagement_rate |   .0000322   .0002097     0.15   0.878                       .0007853
                   rewards |   .0038238   .0025167     1.52   0.129                       .0077803
--------------------------------------------------------------------------------------------

.
. clear all
```

#SCENARIO 2: Sensitivity analysis 20% higher covariance (S2)

```
.
. ssd init age inpatient_hospitalizations er_visits pcp_officevisits specialist_office_visits other_outpatientservices
pharmacy_fills qci_score mpr pdc rewards engagement_rate

Summary statistics data initialized.  Next use, in any order,

    ssd set observations (required)
        It is best to do this first.

    ssd set means (optional)
        Default setting is 0.

    ssd set variances or ssd set sd (optional)
        Use this only if you have set or will set correlations and, even then, this is optional but highly recommended.
Default setting is 1.

    ssd set covariances or ssd set correlations (required)

.
. ssd set observations 37359
  (value set)

    Status:
                       observations:    set
                              means:  unset
                    variances or sd:  unset
        covariances or correlations:  unset (required to be set)

.
. ssd set means 49.64 0.04 0.02 2.18 2.61 2.22 9.95 1.47 0.58 0.59 30 234
  (values set)

    Status:
                       observations:    set
```

```
                              means:    set
                    variances or sd:  unset
        covariances or correlations:  unset (required to be set)

.
. ssd set covariances 221.105233\0.089650 0.067846\-0.094017 0.011868 0.040578\2.451662 0.037385 0.033620
6.244202\3.267802 0.033255 0.022245 6.805591 14.548804\ 5.864529 0.113002 0.075032 7.918511 15
> .837080 22.331055\ 14.209445 0.200045 0.084031 4.385411 7.174931 10.065882 54.346633\ 4.632682 0.036237 0.002199
0.1013640 .076749 0.274862 0.771759 1.282943\ 0.803770 0.000065 -0.001100 0.087459 0.
> 131478 0.182628 1.037027 0.018688 0.129239\ 0.761121 -0.000166 -0.000795 0.047573 0.073374 0.105173 0.708629 0.012082
0.062483 0.082140\-0.2 -0.8 -0.6 0.05 0.1 0.15 0.85 0.002 0.84 0.72 225\-0.1 -.6
>  -0.7 0.08 0.2 0.26 1.04 0.001 0.6 0.552 0.82 32400
  (values set)

    Status:
                       observations:    set
                              means:    set
                    variances or sd:    set
        covariances or correlations:    set
```

. MODEL 11

```
. sem (inpatient_hospitalizations -> mpr, ) (er_visits -> mpr, ) (pcp_officevisits -> mpr, ) (specialist_office_visits
-> mpr, ) (other_outpatientservices -> mpr, ) (pharmacy_fills -> mpr, ) (age -> m
> pr, ) (age -> inpatient_hospitalizations, ) (age -> er_visits, ) (age -> pcp_officevisits, ) (age ->
specialist_office_visits, ) (age -> other_outpatientservices, ) (age -> pharmacy_fills, ) (qci_sc
> ore -> mpr, ) (qci_score -> inpatient_hospitalizations, ) (qci_score -> er_visits, ) (qci_score -> pcp_officevisits,
) (qci_score -> specialist_office_visits, ) (qci_score -> other_outpatientservice
> s, ) (qci_score -> pharmacy_fills, ) (engagement_rate -> mpr, ) (engagement_rate -> inpatient_hospitalizations, )
(engagement_rate -> er_visits, ) (engagement_rate -> pcp_officevisits, ) (engagement
> _rate -> specialist_office_visits, ) (engagement_rate -> other_outpatientservices, ) (engagement_rate ->
pharmacy_fills, ) (rewards -> mpr, ) (rewards -> inpatient_hospitalizations, ) (rewards -> er
> _visits, ) (rewards -> pcp_officevisits, ) (rewards -> specialist_office_visits, ) (rewards ->
other_outpatientservices, ) (rewards -> pharmacy_fills, ), standardized cov( age*qci_score age*engageme
> nt_rate qci_score*engagement_rate engagement_rate*rewards rewards*age rewards*qci_score) nocapslatent

Endogenous variables
```

```
Observed:  inpatient_hospitalizations mpr er_visits pcp_officevisits specialist_office_visits other_outpatientservices
pharmacy_fills

Exogenous variables

Observed:  age qci_score engagement_rate rewards

Fitting target model:

Iteration 0:   log likelihood = -1044408.6
Iteration 1:   log likelihood = -1044408.6

Structural equation model                      Number of obs     =     37,359
Estimation method  = ml
Log likelihood     = -1044408.6

---------------------------------------------------------------------------------------------
                             |                 OIM
                Standardized |      Coef.   Std. Err.      z    P>|z|     [95% Conf. Interval]
-----------------------------+---------------------------------------------------------------
Structural                   |
  inpatient_hospitalizations |
                         age |  -.0117148   .0052245    -2.24   0.025    -.0219546   -.0014749
                   qci_score |   .1260713    .005188    24.30   0.000      .115903    .1362397
             engagement_rate |  -.0127361   .0050229    -2.54   0.011    -.0225808   -.0028914
                     rewards |  -.2047775   .0049186   -41.63   0.000    -.2144177   -.1951372
                       _cons |   .4551753   .0212149    21.46   0.000     .4135949    .4967557
  ---------------------------+---------------------------------------------------------------
  mpr                        |
  inpatient_hospitalizations |  -.0070344   .0049327    -1.43   0.154    -.0167024    .0026336
                   er_visits |  -.0007634   .0048794    -0.16   0.876    -.0103268    .0088001
            pcp_officevisits |   .0131691   .0067364     1.95   0.051     -.000034    .0263722
    specialist_office_visits |   .0006519   .0105371     0.06   0.951    -.0200004    .0213043
    other_outpatientservices |  -.0196226   .0100396    -1.95   0.051    -.0392997    .0000546
              pharmacy_fills |   .3805434   .0045459    83.71   0.000     .3716336    .3894532
                         age |   .1065962   .0048649    21.91   0.000     .0970612    .1161311
                   qci_score |  -.0172794   .0048924    -3.53   0.000    -.0268684   -.0076904
             engagement_rate |   .0088187   .0046592     1.89   0.058    -.0003131    .0179506
                     rewards |   .1511708    .004794    31.53   0.000     .1417747    .1605668
                       _cons |    .448852    .021424    20.95   0.000     .4068617    .4908422
```

er_visits						
age	-.0370254	.0052662	-7.03	0.000	-.0473469	-.0267039
qci_score	.0198455	.0052685	3.77	0.000	.0095195	.0301715
engagement_rate	-.0192466	.0050652	-3.80	0.000	-.0291743	-.009319
rewards	-.1986002	.0049654	-40.00	0.000	-.2083321	-.1888682
_cons	.6193624	.0212275	29.18	0.000	.5777573	.6609675
pcp_officevisits						
age	.0607266	.0053595	11.33	0.000	.0502222	.071231
qci_score	.0191094	.0053677	3.56	0.000	.0085888	.02963
engagement_rate	.0001796	.0051616	0.03	0.972	-.0099369	.0102961
rewards	.0013861	.0051616	0.27	0.788	-.0087304	.0115026
_cons	.6418804	.0222031	28.91	0.000	.5983632	.6853977
specialist_office_visits						
age	.0570474	.0053642	10.63	0.000	.0465337	.0675611
qci_score	.0020729	.0053723	0.39	0.700	-.0084567	.0126024
engagement_rate	.0002929	.0051651	0.06	0.955	-.0098305	.0104163
rewards	.0017986	.0051651	0.35	0.728	-.0083248	.011922
_cons	.487163	.0221029	22.04	0.000	.4438422	.5304838
other_outpatientservices						
age	.0750126	.0053462	14.03	0.000	.0645342	.085491
qci_score	.0307186	.0053578	5.73	0.000	.0202174	.0412197
engagement_rate	.0003077	.0051534	0.06	0.952	-.0097928	.0104081
rewards	.0021797	.0051534	0.42	0.672	-.0079207	.0122801
_cons	.1747415	.0219364	7.97	0.000	.1317469	.2177361
pharmacy_fills						
age	.1127401	.005295	21.29	0.000	.1023621	.1231181
qci_score	.0614141	.0053171	11.55	0.000	.0509929	.0718354
engagement_rate	.0007853	.0051208	0.15	0.878	-.0092513	.0108219
rewards	.0077803	.0051206	1.52	0.129	-.0022559	.0178166
_cons	.877058	.0224515	39.06	0.000	.8330539	.9210621
mean(age)	3.338398	.0132637	251.69	0.000	3.312401	3.364394
mean(qci_score)	1.297835	.0070221	184.82	0.000	1.284072	1.311598
mean(engagement_rate)	1.300017	.0070275	184.99	0.000	1.286244	1.313791
mean(rewards)	2.000027	.0089612	223.19	0.000	1.982463	2.01759

```
-----------------------------+----------------------------------------------------------------
var(e.inpatient_hospitaliz~s)|   .9426941   .0023351                      .9381285    .9472819
                  var(e.mpr)|   .8106312   .0037924                      .8032323    .8180983
            var(e.er_visits)|   .9588389   .0020129                      .9549018    .9627922
     var(e.pcp_officevisits)|   .9953069   .0007055                      .9939251    .9966907
var(e.specialist_office_vi~s)|   .9966731   .0005948                      .9955079    .9978397
var(e.other_outpatientserv~s)|   .9921573   .0009092                      .9903769    .9939408
        var(e.pharmacy_fills)|   .9796493   .0014461                      .9768192    .9824877
                    var(age)|          1          .                             .           .
              var(qci_score)|          1          .                             .           .
        var(engagement_rate)|          1          .                             .           .
                var(rewards)|          1          .                             .           .
-----------------------------+----------------------------------------------------------------
          cov(age,qci_score)|   .2750612   .0047823    57.52   0.000     .2656881    .2844343
    cov(age,engagement_rate)|  -.0000374   .0051737    -0.01   0.994    -.0101777    .0101029
            cov(age,rewards)|  -.0008967   .0051737    -0.17   0.862     -.011037    .0092436
              cov(qci_score,|
            engagement_rate)|   4.90e-06   .0051737     0.00   0.999    -.0101354    .0101452
      cov(qci_score,rewards)|   .0001177   .0051737     0.02   0.982    -.0100226     .010258
 cov(engagement_rate,rewards)|   .0003037   .0051737     0.06   0.953    -.0098366     .010444
----------------------------------------------------------------------------------------------
Note: The LR test of model vs. saturated is not reported because the fitted
      model is not full rank.

.
. estat gof, stats(all)

----------------------------------------------------------------------------
Fit statistic        |      Value   Description
---------------------+------------------------------------------------------
Likelihood ratio     |
          chi2_ms(.) |          .   model vs. saturated
            p > chi2 |          .
         chi2_bs(49) | 101260.960   baseline vs. saturated
            p > chi2 |      0.000
---------------------+------------------------------------------------------
Population error     |
               RMSEA |          .   Root mean squared error of approximation
 90% CI, lower bound |      0.000
         upper bound |          .
```

```
             pclose |          .   Probability RMSEA <= 0.05
--------------------+------------------------------------------------------
Information criteria |
                AIC |  2.089e+06   Akaike's information criterion
                BIC |  2.089e+06   Bayesian information criterion
--------------------+------------------------------------------------------
Baseline comparison  |
                CFI |      1.000   Comparative fit index
                TLI |          .   Tucker-Lewis index
--------------------+------------------------------------------------------
Size of residuals    |
               SRMR |      0.174   Standardized root mean squared residual
                 CD |      0.159   Coefficient of determination
---------------------------------------------------------------------------

.
. estat teffects, standardized


Direct effects
---------------------------------------------------------------------------------------------
                            |                OIM
                            |      Coef.   Std. Err.      z    P>|z|               Std. Coef.
----------------------------+----------------------------------------------------------------
Structural                  |
  inpatient_hospitalizations |
                        age |  -.0002052   .0000915    -2.24   0.025                -.0117148
                  qci_score |   .0289918   .0012015    24.13   0.000                 .1260713
            engagement_rate |  -.0000184   7.27e-06    -2.54   0.011                -.0127361
                    rewards |  -.0035559   .0000872   -40.77   0.000                -.2047775
  --------------------------+----------------------------------------------------------------
  mpr                       |
  inpatient_hospitalizations |  -.0097214   .0068182    -1.43   0.154                -.0070344
                  er_visits |  -.0013641   .0087194    -0.16   0.876                -.0007634
           pcp_officevisits |   .0018971   .0009704     1.96   0.051                 .0131691
    specialist_office_visits |   .0000615   .0009944     0.06   0.951                 .0006519
    other_outpatientservices |  -.0014947   .0007653    -1.95   0.051                -.0196226
             pharmacy_fills |   .0185815   .0002407    77.21   0.000                 .3805434
                        age |   .0025805   .0001183    21.81   0.000                 .1065962
                  qci_score |  -.0054915   .0015548    -3.53   0.000                -.0172794
```

engagement_rate	.0000176	9.32e-06	1.89	0.058	.0088187
rewards	.0036278	.0001159	31.30	0.000	.1511708
**er_visits**					
age	-.0005016	.0000714	-7.03	0.000	-.0370254
qci_score	.0035294	.0009371	3.77	0.000	.0198455
engagement_rate	-.0000215	5.67e-06	-3.80	0.000	-.0192466
rewards	-.0026671	.000068	-39.20	0.000	-.1986002
**pcp_officevisits**					
age	.0102051	.0009022	11.31	0.000	.0607266
qci_score	.0421582	.011844	3.56	0.000	.0191094
engagement_rate	2.49e-06	.0000717	0.03	0.972	.0001796
rewards	.0002309	.0008599	0.27	0.788	.0013861
**specialist_office_visits**					
age	.0146336	.0013781	10.62	0.000	.0570474
qci_score	.0069804	.0180914	0.39	0.700	.0020729
engagement_rate	6.21e-06	.0001095	0.06	0.955	.0002929
rewards	.0004574	.0013134	0.35	0.728	.0017986
**other_outpatientservices**					
age	.0238391	.0017035	13.99	0.000	.0750126
qci_score	.1281599	.0223629	5.73	0.000	.0307186
engagement_rate	8.08e-06	.0001353	0.06	0.952	.0003077
rewards	.0006867	.0016235	0.42	0.672	.0021797
**pharmacy_fills**					
age	.055894	.0026406	21.17	0.000	.1127401
qci_score	.3997155	.034666	11.53	0.000	.0614141
engagement_rate	.0000322	.0002097	0.15	0.878	.0007853
rewards	.0038238	.0025167	1.52	0.129	.0077803

Indirect effects

	Coef.	OIM Std. Err.	z	P>|z|	Std. Coef.

```
Structural                    |
  inpatient_hospitalizations |
                         age |          0  (no path)                                                 0
                   qci_score |          0  (no path)                                                 0
             engagement_rate |          0  (no path)                                                 0
                     rewards |          0  (no path)                                                 0
  ---------------------------+------------------------------------------------------------------------
  mpr                        |
  inpatient_hospitalizations |          0  (no path)                                                 0
                   er_visits |          0  (no path)                                                 0
            pcp_officevisits |          0  (no path)                                                 0
    specialist_office_visits |          0  (no path)                                                 0
    other_outpatientservices |          0  (no path)                                                 0
              pharmacy_fills |          0  (no path)                                                 0
                         age |   .0010259   .0000515    19.91   0.000                         .0423781
                   qci_score |   .0070295   .0006807    10.33   0.000                          .022119
             engagement_rate |   7.99e-07   3.91e-06     0.20   0.838                         .0003996
                     rewards |   .0001087    .000056     1.94   0.052                         .0045295
  ---------------------------+------------------------------------------------------------------------
  er_visits                  |
                         age |          0  (no path)                                                 0
                   qci_score |          0  (no path)                                                 0
             engagement_rate |          0  (no path)                                                 0
                     rewards |          0  (no path)                                                 0
  ---------------------------+------------------------------------------------------------------------
  pcp_officevisits           |
                         age |          0  (no path)                                                 0
                   qci_score |          0  (no path)                                                 0
             engagement_rate |          0  (no path)                                                 0
                     rewards |          0  (no path)                                                 0
  ---------------------------+------------------------------------------------------------------------
  specialist_office_visits   |
                         age |          0  (no path)                                                 0
                   qci_score |          0  (no path)                                                 0
             engagement_rate |          0  (no path)                                                 0
                     rewards |          0  (no path)                                                 0
  ---------------------------+------------------------------------------------------------------------
  other_outpatientservices   |
                         age |          0  (no path)                                                 0
                   qci_score |          0  (no path)                                                 0
```

	Coef.	Std. Err.	z	P>|z|	Std. Coef.
engagement_rate	0	(no path)			0
rewards	0	(no path)			0
**pharmacy_fills**					
age	0	(no path)			0
qci_score	0	(no path)			0
engagement_rate	0	(no path)			0
rewards	0	(no path)			0

Total effects

	Coef.	OIM Std. Err.	z	P>|z|	Std. Coef.
**Structural**					
**inpatient_hospitalizations**					
age	-.0002052	.0000915	-2.24	0.025	-.0117148
qci_score	.0289918	.0012015	24.13	0.000	.1260713
engagement_rate	-.0000184	7.27e-06	-2.54	0.011	-.0127361
rewards	-.0035559	.0000872	-40.77	0.000	-.2047775
**mpr**					
inpatient_hospitalizations	-.0097214	.0068182	-1.43	0.154	-.0070344
er_visits	-.0013641	.0087194	-0.16	0.876	-.0007634
pcp_officevisits	.0018971	.0009704	1.96	0.051	.0131691
specialist_office_visits	.0000615	.0009944	0.06	0.951	.0006519
other_outpatientservices	-.0014947	.0007653	-1.95	0.051	-.0196226
pharmacy_fills	.0185815	.0002407	77.21	0.000	.3805434
age	.0036064	.0001272	28.36	0.000	.1489743
qci_score	.001538	.0016696	0.92	0.357	.0048396
engagement_rate	.0000184	.0000101	1.83	0.068	.0092184
rewards	.0037365	.0001212	30.83	0.000	.1557003
**er_visits**					
age	-.0005016	.0000714	-7.03	0.000	-.0370254
qci_score	.0035294	.0009371	3.77	0.000	.0198455
engagement_rate	-.0000215	5.67e-06	-3.80	0.000	-.0192466
rewards	-.0026671	.000068	-39.20	0.000	-.1986002

```
  --------------------------+----------------------------------------------------------------
  pcp_officevisits          |
                        age |   .0102051   .0009022    11.31   0.000                   .0607266
                  qci_score |   .0421582    .011844     3.56   0.000                   .0191094
            engagement_rate |   2.49e-06   .0000717     0.03   0.972                   .0001796
                    rewards |   .0002309   .0008599     0.27   0.788                   .0013861
  --------------------------+----------------------------------------------------------------
  specialist_office_visits  |
                        age |   .0146336   .0013781    10.62   0.000                   .0570474
                  qci_score |   .0069804   .0180914     0.39   0.700                   .0020729
            engagement_rate |   6.21e-06   .0001095     0.06   0.955                   .0002929
                    rewards |   .0004574   .0013134     0.35   0.728                   .0017986
  --------------------------+----------------------------------------------------------------
  other_outpatientservices  |
                        age |   .0238391   .0017035    13.99   0.000                   .0750126
                  qci_score |   .1281599   .0223629     5.73   0.000                   .0307186
            engagement_rate |   8.08e-06   .0001353     0.06   0.952                   .0003077
                    rewards |   .0006867   .0016235     0.42   0.672                   .0021797
  --------------------------+----------------------------------------------------------------
  pharmacy_fills            |
                        age |    .055894   .0026406    21.17   0.000                   .1127401
                  qci_score |   .3997155    .034666    11.53   0.000                   .0614141
            engagement_rate |   .0000322   .0002097     0.15   0.878                   .0007853
                    rewards |   .0038238   .0025167     1.52   0.129                   .0077803
----------------------------------------------------------------------------------------------
```

. MODEL 12

```
. sem (inpatient_hospitalizations -> pdc, ) (er_visits -> pdc, ) (pcp_officevisits -> pdc, ) (specialist_office_visits
-> pdc, ) (other_outpatientservices -> pdc, ) (pharmacy_fills -> pdc, ) (age -> p
> dc, ) (age -> inpatient_hospitalizations, ) (age -> er_visits, ) (age -> pcp_officevisits, ) (age ->
specialist_office_visits, ) (age -> other_outpatientservices, ) (age -> pharmacy_fills, ) (qci_sc
> ore -> pdc, ) (qci_score -> inpatient_hospitalizations, ) (qci_score -> er_visits, ) (qci_score -> pcp_officevisits,
) (qci_score -> specialist_office_visits, ) (qci_score -> other_outpatientservice
> s, ) (qci_score -> pharmacy_fills, ) (engagement_rate -> pdc, ) (engagement_rate -> inpatient_hospitalizations, )
(engagement_rate -> er_visits, ) (engagement_rate -> pcp_officevisits, ) (engagement
> _rate -> specialist_office_visits, ) (engagement_rate -> other_outpatientservices, ) (engagement_rate ->
pharmacy_fills, ) (rewards -> pdc, ) (rewards -> inpatient_hospitalizations, ) (rewards -> er
```

```
> _visits, ) (rewards -> pcp_officevisits, ) (rewards -> specialist_office_visits, ) (rewards ->
other_outpatientservices, ) (rewards -> pharmacy_fills, ), standardized cov( age*qci_score age*engageme
> nt_rate qci_score*engagement_rate engagement_rate*rewards rewards*age rewards*qci_score) nocapslatent

Endogenous variables

Observed:  inpatient_hospitalizations pdc er_visits pcp_officevisits specialist_office_visits other_outpatientservices
pharmacy_fills

Exogenous variables

Observed:  age qci_score engagement_rate rewards

Fitting target model:

Iteration 0:   log likelihood = -1036554.3
Iteration 1:   log likelihood = -1036554.3

Structural equation model                      Number of obs     =     37,359
Estimation method  = ml
Log likelihood     = -1036554.3

----------------------------------------------------------------------------------------------
                             |                 OIM
                Standardized |      Coef.   Std. Err.      z    P>|z|     [95% Conf. Interval]
-----------------------------+----------------------------------------------------------------
Structural                   |
  inpatient_hospitalizations |
                         age |  -.0117148   .0052245    -2.24   0.025    -.0219546   -.0014749
                   qci_score |   .1260713    .005188    24.30   0.000      .115903    .1362397
             engagement_rate |  -.0127361   .0050229    -2.54   0.011    -.0225808   -.0028914
                     rewards |  -.2047775   .0049186   -41.63   0.000    -.2144177   -.1951372
                       _cons |   .4551753   .0212149    21.46   0.000     .4135949    .4967557
  ---------------------------+----------------------------------------------------------------
  pdc                        |
  inpatient_hospitalizations |  -.0005901   .0050093    -0.12   0.906    -.0104081    .0092278
                   er_visits |   .0085799   .0049543     1.73   0.083    -.0011302    .0182901
            pcp_officevisits |  -.0036511    .006839    -0.53   0.593    -.0170554    .0097531
    specialist_office_visits |   .0031143    .010698     0.29   0.771    -.0178534     .024082
    other_outpatientservices |  -.0283741   .0101884    -2.78   0.005     -.048343   -.0084052
```

pharmacy_fills	.3249456	.0047221	68.81	0.000	.3156904	.3342009
age	.1477112	.0049182	30.03	0.000	.1380718	.1573507
qci_score	-.0320332	.0049666	-6.45	0.000	-.0417676	-.0222989
engagement_rate	.0105408	.0047306	2.23	0.026	.001269	.0198126
rewards	.1663465	.0048552	34.26	0.000	.1568305	.1758626
_cons	.8306757	.0224263	37.04	0.000	.786721	.8746305
er_visits						
age	-.0370254	.0052662	-7.03	0.000	-.0473469	-.0267039
qci_score	.0198455	.0052685	3.77	0.000	.0095195	.0301715
engagement_rate	-.0192466	.0050652	-3.80	0.000	-.0291743	-.009319
rewards	-.1986002	.0049654	-40.00	0.000	-.2083321	-.1888682
_cons	.6193624	.0212275	29.18	0.000	.5777573	.6609675
pcp_officevisits						
age	.0607266	.0053595	11.33	0.000	.0502222	.071231
qci_score	.0191094	.0053677	3.56	0.000	.0085888	.02963
engagement_rate	.0001796	.0051616	0.03	0.972	-.0099369	.0102961
rewards	.0013861	.0051616	0.27	0.788	-.0087304	.0115026
_cons	.6418804	.0222031	28.91	0.000	.5983632	.6853977
specialist_office_visits						
age	.0570474	.0053642	10.63	0.000	.0465337	.0675611
qci_score	.0020729	.0053723	0.39	0.700	-.0084567	.0126024
engagement_rate	.0002929	.0051651	0.06	0.955	-.0098305	.0104163
rewards	.0017986	.0051651	0.35	0.728	-.0083248	.011922
_cons	.487163	.0221029	22.04	0.000	.4438422	.5304838
other_outpatientservices						
age	.0750126	.0053462	14.03	0.000	.0645342	.085491
qci_score	.0307186	.0053578	5.73	0.000	.0202174	.0412197
engagement_rate	.0003077	.0051534	0.06	0.952	-.0097928	.0104081
rewards	.0021797	.0051534	0.42	0.672	-.0079207	.0122801
_cons	.1747415	.0219364	7.97	0.000	.1317469	.2177361
pharmacy_fills						
age	.1127401	.005295	21.29	0.000	.1023621	.1231181
qci_score	.0614141	.0053171	11.55	0.000	.0509929	.0718354
engagement_rate	.0007853	.0051208	0.15	0.878	-.0092513	.0108219
rewards	.0077803	.0051206	1.52	0.129	-.0022559	.0178166

```
                      _cons |    .877058   .0224515    39.06   0.000     .8330539    .9210621
----------------------------+----------------------------------------------------------------
                  mean(age)|   3.338398   .0132637   251.69   0.000     3.312401    3.364394
            mean(qci_score)|   1.297835   .0070221   184.82   0.000     1.284072    1.311598
      mean(engagement_rate)|   1.300017   .0070275   184.99   0.000     1.286244    1.313791
              mean(rewards)|   2.000027   .0089612   223.19   0.000     1.982463     2.01759
----------------------------+----------------------------------------------------------------
var(e.inpatient_hospitaliz~s)|   .9426941   .0023351                      .9381285    .9472819
                 var(e.pdc)|   .8356931   .0036633                       .828544     .842904
           var(e.er_visits)|   .9588389   .0020129                      .9549018    .9627922
     var(e.pcp_officevisits)|   .9953069   .0007055                      .9939251    .9966907
var(e.specialist_office_vi~s)|   .9966731   .0005948                      .9955079    .9978397
var(e.other_outpatientserv~s)|   .9921573   .0009092                      .9903769    .9939408
      var(e.pharmacy_fills)|   .9796493   .0014461                      .9768192    .9824877
                   var(age)|          1          .                             .           .
             var(qci_score)|          1          .                             .           .
       var(engagement_rate)|          1          .                             .           .
               var(rewards)|          1          .                             .           .
----------------------------+----------------------------------------------------------------
        cov(age,qci_score)|   .2750612   .0047823    57.52   0.000     .2656881    .2844343
  cov(age,engagement_rate)|  -.0000374   .0051737    -0.01   0.994    -.0101777    .0101029
          cov(age,rewards)|  -.0008967   .0051737    -0.17   0.862     -.011037    .0092436
            cov(qci_score,|
           engagement_rate)|   4.90e-06   .0051737     0.00   0.999    -.0101354    .0101452
    cov(qci_score,rewards)|   .0001177   .0051737     0.02   0.982    -.0100226     .010258
 cov(engagement_rate,rewards)|   .0003037   .0051737     0.06   0.953    -.0098366     .010444
---------------------------------------------------------------------------------------------
Note: The LR test of model vs. saturated is not reported because the fitted
      model is not full rank.

.
. estat gof, stats(all)

----------------------------------------------------------------------------
Fit statistic        |      Value   Description
---------------------+------------------------------------------------------
Likelihood ratio     |
          chi2_ms(.) |          .   model vs. saturated
            p > chi2 |          .
         chi2_bs(49) | 100036.914   baseline vs. saturated
```

```
           p > chi2 |      0.000
---------------------+----------------------------------------------------
Population error     |
               RMSEA |          .   Root mean squared error of approximation
 90% CI, lower bound |      0.000
         upper bound |          .
              pclose |          .   Probability RMSEA <= 0.05
---------------------+----------------------------------------------------
Information criteria |
                 AIC |  2.073e+06   Akaike's information criterion
                 BIC |  2.074e+06   Bayesian information criterion
---------------------+----------------------------------------------------
Baseline comparison  |
                 CFI |      1.000   Comparative fit index
                 TLI |          .   Tucker-Lewis index
---------------------+----------------------------------------------------
Size of residuals    |
                SRMR |      0.173   Standardized root mean squared residual
                  CD |      0.171   Coefficient of determination
--------------------------------------------------------------------------

.
. estat teffects, standardized


Direct effects
----------------------------------------------------------------------------------------------
                             |                OIM
                             |      Coef.   Std. Err.      z    P>|z|              Std. Coef.
-----------------------------+----------------------------------------------------------------
Structural                   |
  inpatient_hospitalizations |
                         age |  -.0002052   .0000915    -2.24   0.025               -.0117148
                   qci_score |   .0289918   .0012015    24.13   0.000                .1260713
             engagement_rate |  -.0000184   7.27e-06    -2.54   0.011               -.0127361
                     rewards |  -.0035559   .0000872   -40.77   0.000               -.2047775
  ---------------------------+----------------------------------------------------------------
  pdc                        |
  inpatient_hospitalizations |  -.0006509   .0055254    -0.12   0.906               -.0005901
                   er_visits |   .0122372   .0070661     1.73   0.083                .0085799
```

pcp_officevisits	-.0004198	.0007864	-0.53	0.593	-.0036511
specialist_office_visits	.0002346	.0008059	0.29	0.771	.0031143
other_outpatientservices	-.0017251	.0006202	-2.78	0.005	-.0283741
pharmacy_fills	.012664	.000195	64.93	0.000	.3249456
age	.002854	.0000959	29.77	0.000	.1477112
qci_score	-.0081254	.00126	-6.45	0.000	-.0320332
engagement_rate	.0000168	7.55e-06	2.23	0.026	.0105408
rewards	.0031862	.0000939	33.92	0.000	.1663465
er_visits					
age	-.0005016	.0000714	-7.03	0.000	-.0370254
qci_score	.0035294	.0009371	3.77	0.000	.0198455
engagement_rate	-.0000215	5.67e-06	-3.80	0.000	-.0192466
rewards	-.0026671	.000068	-39.20	0.000	-.1986002
pcp_officevisits					
age	.0102051	.0009022	11.31	0.000	.0607266
qci_score	.0421582	.011844	3.56	0.000	.0191094
engagement_rate	2.49e-06	.0000717	0.03	0.972	.0001796
rewards	.0002309	.0008599	0.27	0.788	.0013861
specialist_office_visits					
age	.0146336	.0013781	10.62	0.000	.0570474
qci_score	.0069804	.0180914	0.39	0.700	.0020729
engagement_rate	6.21e-06	.0001095	0.06	0.955	.0002929
rewards	.0004574	.0013134	0.35	0.728	.0017986
other_outpatientservices					
age	.0238391	.0017035	13.99	0.000	.0750126
qci_score	.1281599	.0223629	5.73	0.000	.0307186
engagement_rate	8.08e-06	.0001353	0.06	0.952	.0003077
rewards	.0006867	.0016235	0.42	0.672	.0021797
pharmacy_fills					
age	.055894	.0026406	21.17	0.000	.1127401
qci_score	.3997155	.034666	11.53	0.000	.0614141
engagement_rate	.0000322	.0002097	0.15	0.878	.0007853
rewards	.0038238	.0025167	1.52	0.129	.0077803

```
Indirect effects
-----------------------------------------------------------------------------------------------
                             |                 OIM
                             |      Coef.   Std. Err.      z    P>|z|               Std. Coef.
-----------------------------+-----------------------------------------------------------------
Structural                   |
  inpatient_hospitalizations |
                         age |          0  (no path)                                         0
                   qci_score |          0  (no path)                                         0
             engagement_rate |          0  (no path)                                         0
                     rewards |          0  (no path)                                         0
  ---------------------------+-----------------------------------------------------------------
  pdc                        |
  inpatient_hospitalizations |          0  (no path)                                         0
                   er_visits |          0  (no path)                                         0
            pcp_officevisits |          0  (no path)                                         0
    specialist_office_visits |          0  (no path)                                         0
    other_outpatientservices |          0  (no path)                                         0
              pharmacy_fills |          0  (no path)                                         0
                         age |   .0006599   .0000358    18.41   0.000                 .0341512
                   qci_score |   .0048492   .0004744    10.22   0.000                 .0191172
             engagement_rate |   1.42e-07   2.67e-06     0.05   0.958                 .0000891
                     rewards |   .0000169   .0000405     0.42   0.676                 .0008838
  ---------------------------+-----------------------------------------------------------------
  er_visits                  |
                         age |          0  (no path)                                         0
                   qci_score |          0  (no path)                                         0
             engagement_rate |          0  (no path)                                         0
                     rewards |          0  (no path)                                         0
  ---------------------------+-----------------------------------------------------------------
  pcp_officevisits           |
                         age |          0  (no path)                                         0
                   qci_score |          0  (no path)                                         0
             engagement_rate |          0  (no path)                                         0
                     rewards |          0  (no path)                                         0
  ---------------------------+-----------------------------------------------------------------
  specialist_office_visits   |
                         age |          0  (no path)                                         0
                   qci_score |          0  (no path)                                         0
```

```
            engagement_rate |          0  (no path)                                           0
                    rewards |          0  (no path)                                           0
  --------------------------+----------------------------------------------------------------
  other_outpatientservices   |
                        age |          0  (no path)                                           0
                  qci_score |          0  (no path)                                           0
            engagement_rate |          0  (no path)                                           0
                    rewards |          0  (no path)                                           0
  --------------------------+----------------------------------------------------------------
  pharmacy_fills             |
                        age |          0  (no path)                                           0
                  qci_score |          0  (no path)                                           0
            engagement_rate |          0  (no path)                                           0
                    rewards |          0  (no path)                                           0
-----------------------------------------------------------------------------------------------


Total effects
-----------------------------------------------------------------------------------------------
                             |                 OIM
                             |      Coef.   Std. Err.      z    P>|z|               Std. Coef.
-----------------------------+-----------------------------------------------------------------
Structural                   |
  inpatient_hospitalizations |
                         age |  -.0002052   .0000915    -2.24   0.025               -.0117148
                   qci_score |   .0289918   .0012015    24.13   0.000                .1260713
             engagement_rate |  -.0000184   7.27e-06    -2.54   0.011               -.0127361
                     rewards |  -.0035559   .0000872   -40.77   0.000               -.2047775
  ---------------------------+-----------------------------------------------------------------
  pdc                        |
  inpatient_hospitalizations |  -.0006509   .0055254    -0.12   0.906               -.0005901
                   er_visits |   .0122372   .0070661     1.73   0.083                .0085799
            pcp_officevisits |  -.0004198   .0007864    -0.53   0.593               -.0036511
    specialist_office_visits |   .0002346   .0008059     0.29   0.771                .0031143
    other_outpatientservices |  -.0017251   .0006202    -2.78   0.005               -.0283741
              pharmacy_fills |    .012664    .000195    64.93   0.000                .3249456
                         age |   .0035139   .0001008    34.86   0.000                .1818624
                   qci_score |  -.0032762   .0013234    -2.48   0.013               -.0129161
             engagement_rate |    .000017   8.01e-06     2.12   0.034                .0106299
                     rewards |   .0032031   .0000961    33.34   0.000                .1672303
```

```
--------------------------+----------------------------------------------------------------
er_visits                 |
                      age |  -.0005016   .0000714    -7.03   0.000                -.0370254
                qci_score |   .0035294   .0009371     3.77   0.000                 .0198455
          engagement_rate |  -.0000215   5.67e-06    -3.80   0.000                -.0192466
                  rewards |  -.0026671    .000068   -39.20   0.000                -.1986002
--------------------------+----------------------------------------------------------------
pcp_officevisits          |
                      age |   .0102051   .0009022    11.31   0.000                 .0607266
                qci_score |   .0421582    .011844     3.56   0.000                 .0191094
          engagement_rate |   2.49e-06   .0000717     0.03   0.972                 .0001796
                  rewards |   .0002309   .0008599     0.27   0.788                 .0013861
--------------------------+----------------------------------------------------------------
specialist_office_visits  |
                      age |   .0146336   .0013781    10.62   0.000                 .0570474
                qci_score |   .0069804   .0180914     0.39   0.700                 .0020729
          engagement_rate |   6.21e-06   .0001095     0.06   0.955                 .0002929
                  rewards |   .0004574   .0013134     0.35   0.728                 .0017986
--------------------------+----------------------------------------------------------------
other_outpatientservices  |
                      age |   .0238391   .0017035    13.99   0.000                 .0750126
                qci_score |   .1281599   .0223629     5.73   0.000                 .0307186
          engagement_rate |   8.08e-06   .0001353     0.06   0.952                 .0003077
                  rewards |   .0006867   .0016235     0.42   0.672                 .0021797
--------------------------+----------------------------------------------------------------
pharmacy_fills            |
                      age |    .055894   .0026406    21.17   0.000                 .1127401
                qci_score |   .3997155    .034666    11.53   0.000                 .0614141
          engagement_rate |   .0000322   .0002097     0.15   0.878                 .0007853
                  rewards |   .0038238   .0025167     1.52   0.129                 .0077803
-------------------------------------------------------------------------------------------

.
. clear all
```

#SCENARIO 3: Sensitivity analysis 10% lower covariance (S3)

```
.
. ssd init age inpatient_hospitalizations er_visits pcp_officevisits specialist_office_visits other_outpatientservices
pharmacy_fills qci_score mpr pdc rewards engagement_rate

Summary statistics data initialized.  Next use, in any order,

    ssd set observations (required)
        It is best to do this first.

    ssd set means (optional)
        Default setting is 0.

    ssd set variances or ssd set sd (optional)
        Use this only if you have set or will set correlations and, even then, this is optional but highly recommended.
Default setting is 1.

    ssd set covariances or ssd set correlations (required)

.
. ssd set observations 37359
  (value set)

    Status:
                      observations:    set
                             means:  unset
                   variances or sd:  unset
        covariances or correlations:  unset (required to be set)

.
. ssd set means 49.64 0.04 0.02 2.18 2.61 2.22 9.95 1.47 0.58 0.59 30 234
  (values set)

    Status:
                      observations:    set
                             means:    set
                   variances or sd:  unset
        covariances or correlations:  unset (required to be set)

.
```

```
. ssd set covariances 221.105233\0.089650 0.067846\-0.094017 0.011868 0.040578\2.451662 0.037385 0.033620
6.244202\3.267802 0.033255 0.022245 6.805591 14.548804\ 5.864529 0.113002 0.075032 7.918511 15
> .837080 22.331055\ 14.209445 0.200045 0.084031 4.385411 7.174931 10.065882 54.346633\ 4.632682 0.036237 0.002199
0.1013640 .076749 0.274862 0.771759 1.282943\ 0.803770 0.000065 -0.001100 0.087459 0.
> 131478 0.182628 1.037027 0.018688 0.129239\ 0.761121 -0.000166 -0.000795 0.047573 0.073374 0.105173 0.708629 0.012082
0.062483 0.082140\-0.2 -0.8 -0.6 0.05 0.1 0.15 0.85 0.002 0.63 0.54 225\-0.1 -.6
>  -0.7 0.08 0.2 0.26 1.04 0.001 0.45 0.414 0.82 32400
  (values set)

    Status:
                      observations:    set
                             means:    set
                   variances or sd:    set
        covariances or correlations:    set
```

. MODEL 13

```
. sem (inpatient_hospitalizations -> mpr, ) (er_visits -> mpr, ) (pcp_officevisits -> mpr, ) (specialist_office_visits
-> mpr, ) (other_outpatientservices -> mpr, ) (pharmacy_fills -> mpr, ) (age -> m
> pr, ) (age -> inpatient_hospitalizations, ) (age -> er_visits, ) (age -> pcp_officevisits, ) (age ->
specialist_office_visits, ) (age -> other_outpatientservices, ) (age -> pharmacy_fills, ) (qci_sc
> ore -> mpr, ) (qci_score -> inpatient_hospitalizations, ) (qci_score -> er_visits, ) (qci_score -> pcp_officevisits,
) (qci_score -> specialist_office_visits, ) (qci_score -> other_outpatientservice
> s, ) (qci_score -> pharmacy_fills, ) (engagement_rate -> mpr, ) (engagement_rate -> inpatient_hospitalizations, )
(engagement_rate -> er_visits, ) (engagement_rate -> pcp_officevisits, ) (engagement
> _rate -> specialist_office_visits, ) (engagement_rate -> other_outpatientservices, ) (engagement_rate ->
pharmacy_fills, ) (rewards -> mpr, ) (rewards -> inpatient_hospitalizations, ) (rewards -> er
> _visits, ) (rewards -> pcp_officevisits, ) (rewards -> specialist_office_visits, ) (rewards ->
other_outpatientservices, ) (rewards -> pharmacy_fills, ), standardized cov( age*qci_score age*engageme
> nt_rate qci_score*engagement_rate engagement_rate*rewards rewards*age rewards*qci_score) nocapslatent

Endogenous variables

Observed:  inpatient_hospitalizations mpr er_visits pcp_officevisits specialist_office_visits other_outpatientservices
pharmacy_fills

Exogenous variables

Observed:  age qci_score engagement_rate rewards
```

```
Fitting target model:

Iteration 0:   log likelihood = -1044641.4
Iteration 1:   log likelihood = -1044641.4

Structural equation model                       Number of obs     =     37,359
Estimation method  = ml
Log likelihood     = -1044641.4

----------------------------------------------------------------------------------------------
                             |                 OIM
                Standardized |      Coef.   Std. Err.      z    P>|z|     [95% Conf. Interval]
-----------------------------+----------------------------------------------------------------
Structural                   |
  inpatient_hospitalizations |
                         age |  -.0117148   .0052245    -2.24   0.025    -.0219546   -.0014749
                   qci_score |   .1260713    .005188    24.30   0.000      .115903    .1362397
             engagement_rate |  -.0127361   .0050229    -2.54   0.011    -.0225808   -.0028914
                     rewards |  -.2047775   .0049186   -41.63   0.000    -.2144177   -.1951372
                       _cons |   .4551753   .0212149    21.46   0.000     .4135949    .4967557
  ---------------------------+----------------------------------------------------------------
  mpr                        |
  inpatient_hospitalizations |  -.0145056   .0049611    -2.92   0.003    -.0242292   -.0047821
                   er_visits |  -.0077338   .0049083    -1.58   0.115     -.017354    .0018863
             pcp_officevisits |   .0139072   .0067765     2.05   0.040     .0006255     .027189
   specialist_office_visits |  -.0024934   .0106004    -0.24   0.814    -.0232699     .018283
    other_outpatientservices |  -.0163616   .0101019    -1.62   0.105    -.0361609    .0034377
              pharmacy_fills |   .3816209   .0045663    83.57   0.000     .3726712    .3905707
                         age |   .1059405   .0048941    21.65   0.000     .0963482    .1155328
                   qci_score |   -.016359   .0049217    -3.32   0.001    -.0260053   -.0067127
             engagement_rate |   .0062833   .0046871     1.34   0.180    -.0029033    .0154699
                     rewards |   .1093228   .0048407    22.58   0.000     .0998353    .1188104
                       _cons |   .5367744   .0216318    24.81   0.000     .4943768    .5791719
  ---------------------------+----------------------------------------------------------------
  er_visits                  |
                         age |  -.0370254   .0052662    -7.03   0.000    -.0473469   -.0267039
                   qci_score |   .0198455   .0052685     3.77   0.000     .0095195    .0301715
             engagement_rate |  -.0192466   .0050652    -3.80   0.000    -.0291743    -.009319
                     rewards |  -.1986002   .0049654   -40.00   0.000    -.2083321   -.1888682
```

```
                       _cons |   .6193624   .0212275    29.18   0.000     .5777573    .6609675
  ---------------------------+----------------------------------------------------------------
  pcp_officevisits           |
                         age |   .0607266   .0053595    11.33   0.000     .0502222     .071231
                   qci_score |   .0191094   .0053677     3.56   0.000     .0085888      .02963
             engagement_rate |   .0001796   .0051616     0.03   0.972    -.0099369    .0102961
                     rewards |   .0013861   .0051616     0.27   0.788    -.0087304    .0115026
                       _cons |   .6418804   .0222031    28.91   0.000     .5983632    .6853977
  ---------------------------+----------------------------------------------------------------
  specialist_office_visits   |
                         age |   .0570474   .0053642    10.63   0.000     .0465337    .0675611
                   qci_score |   .0020729   .0053723     0.39   0.700    -.0084567    .0126024
             engagement_rate |   .0002929   .0051651     0.06   0.955    -.0098305    .0104163
                     rewards |   .0017986   .0051651     0.35   0.728    -.0083248     .011922
                       _cons |    .487163   .0221029    22.04   0.000     .4438422    .5304838
  ---------------------------+----------------------------------------------------------------
  other_outpatientservices   |
                         age |   .0750126   .0053462    14.03   0.000     .0645342     .085491
                   qci_score |   .0307186   .0053578     5.73   0.000     .0202174    .0412197
             engagement_rate |   .0003077   .0051534     0.06   0.952    -.0097928    .0104081
                     rewards |   .0021797   .0051534     0.42   0.672    -.0079207    .0122801
                       _cons |   .1747415   .0219364     7.97   0.000     .1317469    .2177361
  ---------------------------+----------------------------------------------------------------
  pharmacy_fills             |
                         age |   .1127401    .005295    21.29   0.000     .1023621    .1231181
                   qci_score |   .0614141   .0053171    11.55   0.000     .0509929    .0718354
             engagement_rate |   .0007853   .0051208     0.15   0.878    -.0092513    .0108219
                     rewards |   .0077803   .0051206     1.52   0.129    -.0022559    .0178166
                       _cons |    .877058   .0224515    39.06   0.000     .8330539    .9210621
-----------------------------+----------------------------------------------------------------
                   mean(age)|   3.338398   .0132637   251.69   0.000     3.312401    3.364394
             mean(qci_score)|   1.297835   .0070221   184.82   0.000     1.284072    1.311598
       mean(engagement_rate)|   1.300017   .0070275   184.99   0.000     1.286244    1.313791
               mean(rewards)|   2.000027   .0089612   223.19   0.000     1.982463     2.01759
-----------------------------+----------------------------------------------------------------
var(e.inpatient_hospitaliz~s)|   .9426941   .0023351                       .9381285    .9472819
                  var(e.mpr)|    .820364   .0037408                       .8130648    .8277288
            var(e.er_visits)|   .9588389   .0020129                       .9549018    .9627922
     var(e.pcp_officevisits)|   .9953069   .0007055                       .9939251    .9966907
var(e.specialist_office_vi~s)|   .9966731   .0005948                       .9955079    .9978397
```

```
var(e.other_outpatientserv~s)|   .9921573   .0009092                      .9903769    .9939408
       var(e.pharmacy_fills)|   .9796493   .0014461                      .9768192    .9824877
                    var(age)|          1          .                             .           .
              var(qci_score)|          1          .                             .           .
         var(engagement_rate)|          1          .                             .           .
                var(rewards)|          1          .                             .           .
-----------------------------+----------------------------------------------------------------
         cov(age,qci_score)|   .2750612   .0047823    57.52   0.000     .2656881    .2844343
    cov(age,engagement_rate)|  -.0000374   .0051737    -0.01   0.994    -.0101777    .0101029
           cov(age,rewards)|  -.0008967   .0051737    -0.17   0.862     -.011037    .0092436
              cov(qci_score,|
            engagement_rate)|   4.90e-06   .0051737     0.00   0.999    -.0101354    .0101452
      cov(qci_score,rewards)|   .0001177   .0051737     0.02   0.982    -.0100226     .010258
 cov(engagement_rate,rewards)|   .0003037   .0051737     0.06   0.953    -.0098366     .010444
----------------------------------------------------------------------------------------------
Note: The LR test of model vs. saturated is not reported because the fitted
      model is not full rank.

.
. estat gof, stats(all)

----------------------------------------------------------------------------
Fit statistic        |      Value   Description
---------------------+------------------------------------------------------
Likelihood ratio     |
          chi2_ms(.) |          .   model vs. saturated
            p > chi2 |          .
         chi2_bs(49) | 100795.299   baseline vs. saturated
            p > chi2 |      0.000
---------------------+------------------------------------------------------
Population error     |
               RMSEA |          .   Root mean squared error of approximation
 90% CI, lower bound |      0.000
         upper bound |          .
              pclose |          .   Probability RMSEA <= 0.05
---------------------+------------------------------------------------------
Information criteria |
                 AIC |  2.089e+06   Akaike's information criterion
                 BIC |  2.090e+06   Bayesian information criterion
---------------------+------------------------------------------------------
```

```
Baseline comparison  |
                 CFI |      1.000   Comparative fit index
                 TLI |          .   Tucker-Lewis index
---------------------+------------------------------------------------
Size of residuals    |
                SRMR |      0.174   Standardized root mean squared residual
                  CD |      0.148   Coefficient of determination
----------------------------------------------------------------------

.
. estat teffects, standardized


Direct effects
-----------------------------------------------------------------------------------------------
                             |                 OIM
                             |      Coef.   Std. Err.      z    P>|z|               Std. Coef.
-----------------------------+-----------------------------------------------------------------
Structural                   |
  inpatient_hospitalizations |
                         age |  -.0002052   .0000915    -2.24   0.025                -.0117148
                   qci_score |   .0289918   .0012015    24.13   0.000                 .1260713
             engagement_rate |  -.0000184   7.27e-06    -2.54   0.011                -.0127361
                     rewards |  -.0035559   .0000872   -40.77   0.000                -.2047775
  ---------------------------+-----------------------------------------------------------------
  mpr                        |
  inpatient_hospitalizations |  -.0200517   .0068608    -2.92   0.003                -.0145056
                   er_visits |  -.0138238   .0087739    -1.58   0.115                -.0077338
            pcp_officevisits |   .0020039   .0009764     2.05   0.040                 .0139072
    specialist_office_visits |  -.0002354   .0010006    -0.24   0.814                -.0024934
    other_outpatientservices |  -.0012467   .0007701    -1.62   0.105                -.0163616
              pharmacy_fills |    .018639   .0002422    76.96   0.000                 .3816209
                         age |   .0025653    .000119    21.55   0.000                 .1059405
                   qci_score |  -.0052003   .0015646    -3.32   0.001                 -.016359
             engagement_rate |   .0000126   9.38e-06     1.34   0.180                 .0062833
                     rewards |   .0026242   .0001166    22.50   0.000                 .1093228
  ---------------------------+-----------------------------------------------------------------
  er_visits                  |
                         age |  -.0005016   .0000714    -7.03   0.000                -.0370254
                   qci_score |   .0035294   .0009371     3.77   0.000                 .0198455
```

engagement_rate	-.0000215	5.67e-06	-3.80	0.000	-.0192466
rewards	-.0026671	.000068	-39.20	0.000	-.1986002
pcp_officevisits					
age	.0102051	.0009022	11.31	0.000	.0607266
qci_score	.0421582	.011844	3.56	0.000	.0191094
engagement_rate	2.49e-06	.0000717	0.03	0.972	.0001796
rewards	.0002309	.0008599	0.27	0.788	.0013861
specialist_office_visits					
age	.0146336	.0013781	10.62	0.000	.0570474
qci_score	.0069804	.0180914	0.39	0.700	.0020729
engagement_rate	6.21e-06	.0001095	0.06	0.955	.0002929
rewards	.0004574	.0013134	0.35	0.728	.0017986
other_outpatientservices					
age	.0238391	.0017035	13.99	0.000	.0750126
qci_score	.1281599	.0223629	5.73	0.000	.0307186
engagement_rate	8.08e-06	.0001353	0.06	0.952	.0003077
rewards	.0006867	.0016235	0.42	0.672	.0021797
pharmacy_fills					
age	.055894	.0026406	21.17	0.000	.1127401
qci_score	.3997155	.034666	11.53	0.000	.0614141
engagement_rate	.0000322	.0002097	0.15	0.878	.0007853
rewards	.0038238	.0025167	1.52	0.129	.0077803

Indirect effects

	Coef.	OIM Std. Err.	z	P>|z|	Std. Coef.
Structural					
inpatient_hospitalizations					
age	0	(no path)			0
qci_score	0	(no path)			0
engagement_rate	0	(no path)			0
rewards	0	(no path)			0

```
--------------------------+----------------------------------------------------------------
mpr                       |
inpatient_hospitalizations |          0  (no path)                                         0
                er_visits |          0  (no path)                                         0
          pcp_officevisits |          0  (no path)                                         0
  specialist_office_visits |          0  (no path)                                         0
  other_outpatientservices |          0  (no path)                                         0
            pharmacy_fills |          0  (no path)                                         0
                       age |   .0010401   .0000517    20.11   0.000                  .0429552
                 qci_score |   .0067432   .0006832     9.87   0.000                  .0212127
           engagement_rate |   1.26e-06   3.92e-06     0.32   0.748                    .00063
                   rewards |   .0001789   .0000562     3.18   0.001                  .0074546
--------------------------+----------------------------------------------------------------
er_visits                 |
                       age |          0  (no path)                                         0
                 qci_score |          0  (no path)                                         0
           engagement_rate |          0  (no path)                                         0
                   rewards |          0  (no path)                                         0
--------------------------+----------------------------------------------------------------
pcp_officevisits          |
                       age |          0  (no path)                                         0
                 qci_score |          0  (no path)                                         0
           engagement_rate |          0  (no path)                                         0
                   rewards |          0  (no path)                                         0
--------------------------+----------------------------------------------------------------
specialist_office_visits  |
                       age |          0  (no path)                                         0
                 qci_score |          0  (no path)                                         0
           engagement_rate |          0  (no path)                                         0
                   rewards |          0  (no path)                                         0
--------------------------+----------------------------------------------------------------
other_outpatientservices  |
                       age |          0  (no path)                                         0
                 qci_score |          0  (no path)                                         0
           engagement_rate |          0  (no path)                                         0
                   rewards |          0  (no path)                                         0
--------------------------+----------------------------------------------------------------
pharmacy_fills            |
                       age |          0  (no path)                                         0
                 qci_score |          0  (no path)                                         0
```

```
            engagement_rate |          0  (no path)                                      0
                    rewards |          0  (no path)                                      0
-----------------------------------------------------------------------------------------------


Total effects
-----------------------------------------------------------------------------------------------
                            |                 OIM
                            |      Coef.   Std. Err.      z    P>|z|                Std. Coef.
----------------------------+------------------------------------------------------------------
Structural                  |
  inpatient_hospitalizations |
                        age |  -.0002052   .0000915    -2.24   0.025                 -.0117148
                  qci_score |   .0289918   .0012015    24.13   0.000                  .1260713
            engagement_rate |  -.0000184   7.27e-06    -2.54   0.011                 -.0127361
                    rewards |  -.0035559   .0000872   -40.77   0.000                 -.2047775
  --------------------------+------------------------------------------------------------------
  mpr                       |
  inpatient_hospitalizations |  -.0200517   .0068608    -2.92   0.003                 -.0145056
                  er_visits |  -.0138238   .0087739    -1.58   0.115                 -.0077338
           pcp_officevisits |   .0020039   .0009764     2.05   0.040                  .0139072
  specialist_office_visits |  -.0002354   .0010006    -0.24   0.814                 -.0024934
  other_outpatientservices |  -.0012467   .0007701    -1.62   0.105                 -.0163616
             pharmacy_fills |    .018639   .0002422    76.96   0.000                  .3816209
                        age |   .0036055   .0001279    28.18   0.000                  .1488957
                  qci_score |   .0015429   .0016794     0.92   0.358                  .0048536
            engagement_rate |   .0000138   .0000102     1.36   0.173                  .0069133
                    rewards |   .0028031   .0001219    22.99   0.000                  .1167775
  --------------------------+------------------------------------------------------------------
  er_visits                 |
                        age |  -.0005016   .0000714    -7.03   0.000                 -.0370254
                  qci_score |   .0035294   .0009371     3.77   0.000                  .0198455
            engagement_rate |  -.0000215   5.67e-06    -3.80   0.000                 -.0192466
                    rewards |  -.0026671    .000068   -39.20   0.000                 -.1986002
  --------------------------+------------------------------------------------------------------
  pcp_officevisits          |
                        age |   .0102051   .0009022    11.31   0.000                  .0607266
                  qci_score |   .0421582    .011844     3.56   0.000                  .0191094
            engagement_rate |   2.49e-06   .0000717     0.03   0.972                  .0001796
                    rewards |   .0002309   .0008599     0.27   0.788                  .0013861
```

```
  -------------------------+----------------------------------------------------------------
  specialist_office_visits   |
                       age |   .0146336   .0013781    10.62   0.000                .0570474
                 qci_score |   .0069804   .0180914     0.39   0.700                .0020729
           engagement_rate |   6.21e-06   .0001095     0.06   0.955                .0002929
                   rewards |   .0004574   .0013134     0.35   0.728                .0017986
  -------------------------+----------------------------------------------------------------
  other_outpatientservices   |
                       age |   .0238391   .0017035    13.99   0.000                .0750126
                 qci_score |   .1281599   .0223629     5.73   0.000                .0307186
           engagement_rate |   8.08e-06   .0001353     0.06   0.952                .0003077
                   rewards |   .0006867   .0016235     0.42   0.672                .0021797
  -------------------------+----------------------------------------------------------------
  pharmacy_fills             |
                       age |    .055894   .0026406    21.17   0.000                .1127401
                 qci_score |   .3997155    .034666    11.53   0.000                .0614141
           engagement_rate |   .0000322   .0002097     0.15   0.878                .0007853
                   rewards |   .0038238   .0025167     1.52   0.129                .0077803
-----------------------------------------------------------------------------------------------
```

. MODEL 14

```
. sem (inpatient_hospitalizations -> pdc, ) (er_visits -> pdc, ) (pcp_officevisits -> pdc, ) (specialist_office_visits
-> pdc, ) (other_outpatientservices -> pdc, ) (pharmacy_fills -> pdc, ) (age -> p
> dc, ) (age -> inpatient_hospitalizations, ) (age -> er_visits, ) (age -> pcp_officevisits, ) (age ->
specialist_office_visits, ) (age -> other_outpatientservices, ) (age -> pharmacy_fills, ) (qci_sc
> ore -> pdc, ) (qci_score -> inpatient_hospitalizations, ) (qci_score -> er_visits, ) (qci_score -> pcp_officevisits,
) (qci_score -> specialist_office_visits, ) (qci_score -> other_outpatientservice
> s, ) (qci_score -> pharmacy_fills, ) (engagement_rate -> pdc, ) (engagement_rate -> inpatient_hospitalizations, )
(engagement_rate -> er_visits, ) (engagement_rate -> pcp_officevisits, ) (engagement
> _rate -> specialist_office_visits, ) (engagement_rate -> other_outpatientservices, ) (engagement_rate ->
pharmacy_fills, ) (rewards -> pdc, ) (rewards -> inpatient_hospitalizations, ) (rewards -> er
> _visits, ) (rewards -> pcp_officevisits, ) (rewards -> specialist_office_visits, ) (rewards ->
other_outpatientservices, ) (rewards -> pharmacy_fills, ), standardized cov( age*qci_score age*engageme
> nt_rate qci_score*engagement_rate engagement_rate*rewards rewards*age rewards*qci_score) nocapslatent

Endogenous variables
```

```
Observed:  inpatient_hospitalizations pdc er_visits pcp_officevisits specialist_office_visits other_outpatientservices
pharmacy_fills

Exogenous variables

Observed:  age qci_score engagement_rate rewards

Fitting target model:

Iteration 0:   log likelihood = -1036822.2
Iteration 1:   log likelihood = -1036822.2

Structural equation model                      Number of obs     =     37,359
Estimation method  = ml
Log likelihood     = -1036822.2

----------------------------------------------------------------------------------------------
                             |                 OIM
                Standardized |      Coef.   Std. Err.      z    P>|z|     [95% Conf. Interval]
-----------------------------+----------------------------------------------------------------
Structural                   |
  inpatient_hospitalizations |
                         age |  -.0117148   .0052245    -2.24   0.025    -.0219546   -.0014749
                   qci_score |   .1260713    .005188    24.30   0.000      .115903    .1362397
             engagement_rate |  -.0127361   .0050229    -2.54   0.011    -.0225808   -.0028914
                     rewards |  -.2047775   .0049186   -41.63   0.000    -.2144177   -.1951372
                       _cons |   .4551753   .0212149    21.46   0.000     .4135949    .4967557
  ---------------------------+----------------------------------------------------------------
  pdc                        |
  inpatient_hospitalizations |  -.0086179   .0050434    -1.71   0.087    -.0185028     .001267
                   er_visits |   .0010884   .0049889     0.22   0.827    -.0086898    .0108665
           pcp_officevisits |  -.0028536    .006887    -0.41   0.679    -.0163519    .0106446
    specialist_office_visits |  -.0002653   .0107737    -0.02   0.980    -.0213813    .0208508
    other_outpatientservices |  -.0248705   .0102628    -2.42   0.015    -.0449853   -.0047558
              pharmacy_fills |   .3261462    .004749    68.68   0.000     .3168383     .335454
                         age |   .1470074   .0049525    29.68   0.000     .1373007    .1567141
                   qci_score |   -.031043   .0050013    -6.21   0.000    -.0408453   -.0212407
             engagement_rate |   .0076358   .0047638     1.60   0.109     -.001701    .0169727
                     rewards |   .1214111   .0049127    24.71   0.000     .1117824    .1310398
                       _cons |   .9253032   .0226539    40.85   0.000     .8809023    .9697041
```

er_visits						
age	-.0370254	.0052662	-7.03	0.000	-.0473469	-.0267039
qci_score	.0198455	.0052685	3.77	0.000	.0095195	.0301715
engagement_rate	-.0192466	.0050652	-3.80	0.000	-.0291743	-.009319
rewards	-.1986002	.0049654	-40.00	0.000	-.2083321	-.1888682
_cons	.6193624	.0212275	29.18	0.000	.5777573	.6609675
pcp_officevisits						
age	.0607266	.0053595	11.33	0.000	.0502222	.071231
qci_score	.0191094	.0053677	3.56	0.000	.0085888	.02963
engagement_rate	.0001796	.0051616	0.03	0.972	-.0099369	.0102961
rewards	.0013861	.0051616	0.27	0.788	-.0087304	.0115026
_cons	.6418804	.0222031	28.91	0.000	.5983632	.6853977
specialist_office_visits						
age	.0570474	.0053642	10.63	0.000	.0465337	.0675611
qci_score	.0020729	.0053723	0.39	0.700	-.0084567	.0126024
engagement_rate	.0002929	.0051651	0.06	0.955	-.0098305	.0104163
rewards	.0017986	.0051651	0.35	0.728	-.0083248	.011922
_cons	.487163	.0221029	22.04	0.000	.4438422	.5304838
other_outpatientservices						
age	.0750126	.0053462	14.03	0.000	.0645342	.085491
qci_score	.0307186	.0053578	5.73	0.000	.0202174	.0412197
engagement_rate	.0003077	.0051534	0.06	0.952	-.0097928	.0104081
rewards	.0021797	.0051534	0.42	0.672	-.0079207	.0122801
_cons	.1747415	.0219364	7.97	0.000	.1317469	.2177361
pharmacy_fills						
age	.1127401	.005295	21.29	0.000	.1023621	.1231181
qci_score	.0614141	.0053171	11.55	0.000	.0509929	.0718354
engagement_rate	.0007853	.0051208	0.15	0.878	-.0092513	.0108219
rewards	.0077803	.0051206	1.52	0.129	-.0022559	.0178166
_cons	.877058	.0224515	39.06	0.000	.8330539	.9210621
mean(age)	3.338398	.0132637	251.69	0.000	3.312401	3.364394
mean(qci_score)	1.297835	.0070221	184.82	0.000	1.284072	1.311598
mean(engagement_rate)	1.300017	.0070275	184.99	0.000	1.286244	1.313791
mean(rewards)	2.000027	.0089612	223.19	0.000	1.982463	2.01759

```
-----------------------------+----------------------------------------------------------------
var(e.inpatient_hospitaliz~s)|   .9426941   .0023351                          .9381285    .9472819
                 var(e.pdc)|   .8474208   .0035792                          .8404346    .8544651
           var(e.er_visits)|   .9588389   .0020129                          .9549018    .9627922
    var(e.pcp_officevisits)|   .9953069   .0007055                          .9939251    .9966907
var(e.specialist_office_vi~s)|   .9966731   .0005948                          .9955079    .9978397
var(e.other_outpatientserv~s)|   .9921573   .0009092                          .9903769    .9939408
      var(e.pharmacy_fills)|   .9796493   .0014461                          .9768192    .9824877
                   var(age)|          1          .                                 .           .
             var(qci_score)|          1          .                                 .           .
       var(engagement_rate)|          1          .                                 .           .
               var(rewards)|          1          .                                 .           .
-----------------------------+----------------------------------------------------------------
         cov(age,qci_score)|   .2750612   .0047823    57.52   0.000     .2656881    .2844343
   cov(age,engagement_rate)|  -.0000374   .0051737    -0.01   0.994    -.0101777    .0101029
           cov(age,rewards)|  -.0008967   .0051737    -0.17   0.862     -.011037    .0092436
             cov(qci_score,|
           engagement_rate)|   4.90e-06   .0051737     0.00   0.999    -.0101354    .0101452
     cov(qci_score,rewards)|   .0001177   .0051737     0.02   0.982    -.0100226     .010258
 cov(engagement_rate,rewards)|   .0003037   .0051737     0.06   0.953    -.0098366     .010444
----------------------------------------------------------------------------------------------
Note: The LR test of model vs. saturated is not reported because the fitted
      model is not full rank.

.
. estat gof, stats(all)

----------------------------------------------------------------------------
Fit statistic        |      Value   Description
---------------------+------------------------------------------------------
Likelihood ratio     |
          chi2_ms(.) |          .   model vs. saturated
            p > chi2 |          .
         chi2_bs(49) |  99501.266   baseline vs. saturated
            p > chi2 |      0.000
---------------------+------------------------------------------------------
Population error     |
               RMSEA |          .   Root mean squared error of approximation
 90% CI, lower bound |      0.000
         upper bound |          .
```

```
             pclose |          .   Probability RMSEA <= 0.05
--------------------+---------------------------------------------------
Information criteria |
                AIC |  2.074e+06   Akaike's information criterion
                BIC |  2.074e+06   Bayesian information criterion
--------------------+---------------------------------------------------
Baseline comparison  |
                CFI |      1.000   Comparative fit index
                TLI |          .   Tucker-Lewis index
--------------------+---------------------------------------------------
Size of residuals    |
               SRMR |      0.173   Standardized root mean squared residual
                 CD |      0.159   Coefficient of determination
------------------------------------------------------------------------

.
. estat teffects, standardized


Direct effects
----------------------------------------------------------------------------------------------
                            |                 OIM
                            |      Coef.   Std. Err.      z    P>|z|               Std. Coef.
----------------------------+-----------------------------------------------------------------
Structural                  |
  inpatient_hospitalizations |
                        age |  -.0002052   .0000915    -2.24   0.025                 -.0117148
                  qci_score |   .0289918   .0012015    24.13   0.000                  .1260713
            engagement_rate |  -.0000184   7.27e-06    -2.54   0.011                 -.0127361
                    rewards |  -.0035559   .0000872   -40.77   0.000                 -.2047775
  --------------------------+-----------------------------------------------------------------
  pdc                       |
  inpatient_hospitalizations |  -.0095076   .0055651    -1.71   0.088                 -.0086179
                  er_visits |   .0015526    .007117     0.22   0.827                  .0010884
          pcp_officevisits |  -.0003282    .000792    -0.41   0.679                 -.0028536
   specialist_office_visits |    -.00002   .0008117    -0.02   0.980                 -.0002653
   other_outpatientservices |  -.0015124   .0006247    -2.42   0.015                 -.0248705
             pharmacy_fills |   .0127133   .0001964    64.72   0.000                  .3261462
                        age |    .002841   .0000966    29.42   0.000                  .1470074
                  qci_score |  -.0078758   .0012691    -6.21   0.000                  -.031043
```

```
            engagement_rate |   .0000122   7.61e-06     1.60   0.109                 .0076358
                    rewards |   .0023259   .0000946    24.59   0.000                 .1214111
  --------------------------+----------------------------------------------------------------
  er_visits                 |
                        age |  -.0005016   .0000714    -7.03   0.000                -.0370254
                  qci_score |   .0035294   .0009371     3.77   0.000                 .0198455
            engagement_rate |  -.0000215   5.67e-06    -3.80   0.000                -.0192466
                    rewards |  -.0026671    .000068   -39.20   0.000                -.1986002
  --------------------------+----------------------------------------------------------------
  pcp_officevisits          |
                        age |   .0102051   .0009022    11.31   0.000                 .0607266
                  qci_score |   .0421582    .011844     3.56   0.000                 .0191094
            engagement_rate |   2.49e-06   .0000717     0.03   0.972                 .0001796
                    rewards |   .0002309   .0008599     0.27   0.788                 .0013861
  --------------------------+----------------------------------------------------------------
  specialist_office_visits  |
                        age |   .0146336   .0013781    10.62   0.000                 .0570474
                  qci_score |   .0069804   .0180914     0.39   0.700                 .0020729
            engagement_rate |   6.21e-06   .0001095     0.06   0.955                 .0002929
                    rewards |   .0004574   .0013134     0.35   0.728                 .0017986
  --------------------------+----------------------------------------------------------------
  other_outpatientservices  |
                        age |   .0238391   .0017035    13.99   0.000                 .0750126
                  qci_score |   .1281599   .0223629     5.73   0.000                 .0307186
            engagement_rate |   8.08e-06   .0001353     0.06   0.952                 .0003077
                    rewards |   .0006867   .0016235     0.42   0.672                 .0021797
  --------------------------+----------------------------------------------------------------
  pharmacy_fills            |
                        age |    .055894   .0026406    21.17   0.000                 .1127401
                  qci_score |   .3997155    .034666    11.53   0.000                 .0614141
            engagement_rate |   .0000322   .0002097     0.15   0.878                 .0007853
                    rewards |   .0038238   .0025167     1.52   0.129                 .0077803
----------------------------------------------------------------------------------------------


Indirect effects
----------------------------------------------------------------------------------------------
                            |                 OIM
                            |      Coef.   Std. Err.      z    P>|z|               Std. Coef.
----------------------------+-----------------------------------------------------------------
```

```
Structural                    |
  inpatient_hospitalizations |
                         age |          0  (no path)                                                0
                   qci_score |          0  (no path)                                                0
             engagement_rate |          0  (no path)                                                0
                     rewards |          0  (no path)                                                0
  ---------------------------+---------------------------------------------------------------------
  pdc                        |
  inpatient_hospitalizations |          0  (no path)                                                0
                   er_visits |          0  (no path)                                                0
            pcp_officevisits |          0  (no path)                                                0
    specialist_office_visits |          0  (no path)                                                0
    other_outpatientservices |          0  (no path)                                                0
              pharmacy_fills |          0  (no path)                                                0
                         age |   .0006721    .000036    18.69   0.000                       .0347764
                   qci_score |   .0046037    .000476     9.67   0.000                        .018146
             engagement_rate |   5.38e-07   2.68e-06     0.20   0.841                       .0003367
                     rewards |   .0000772   .0000407     1.90   0.058                       .0040275
  ---------------------------+---------------------------------------------------------------------
  er_visits                  |
                         age |          0  (no path)                                                0
                   qci_score |          0  (no path)                                                0
             engagement_rate |          0  (no path)                                                0
                     rewards |          0  (no path)                                                0
  ---------------------------+---------------------------------------------------------------------
  pcp_officevisits           |
                         age |          0  (no path)                                                0
                   qci_score |          0  (no path)                                                0
             engagement_rate |          0  (no path)                                                0
                     rewards |          0  (no path)                                                0
  ---------------------------+---------------------------------------------------------------------
  specialist_office_visits   |
                         age |          0  (no path)                                                0
                   qci_score |          0  (no path)                                                0
             engagement_rate |          0  (no path)                                                0
                     rewards |          0  (no path)                                                0
  ---------------------------+---------------------------------------------------------------------
  other_outpatientservices   |
                         age |          0  (no path)                                                0
                   qci_score |          0  (no path)                                                0
```

```
           engagement_rate |          0  (no path)                                          0
                   rewards |          0  (no path)                                          0
  -------------------------+----------------------------------------------------------------
  pharmacy_fills           |
                       age |          0  (no path)                                          0
                 qci_score |          0  (no path)                                          0
           engagement_rate |          0  (no path)                                          0
                   rewards |          0  (no path)                                          0
-----------------------------------------------------------------------------------------------


Total effects
-----------------------------------------------------------------------------------------------
                            |                 OIM
                            |      Coef.   Std. Err.      z    P>|z|                Std. Coef.
----------------------------+------------------------------------------------------------------
Structural                  |
  inpatient_hospitalizations |
                        age |  -.0002052   .0000915    -2.24   0.025                 -.0117148
                  qci_score |   .0289918   .0012015    24.13   0.000                  .1260713
            engagement_rate |  -.0000184   7.27e-06    -2.54   0.011                 -.0127361
                    rewards |  -.0035559   .0000872   -40.77   0.000                 -.2047775
  --------------------------+------------------------------------------------------------------
  pdc                       |
  inpatient_hospitalizations |  -.0095076   .0055651    -1.71   0.088                 -.0086179
                  er_visits |   .0015526    .007117     0.22   0.827                  .0010884
           pcp_officevisits |  -.0003282    .000792    -0.41   0.679                 -.0028536
    specialist_office_visits |    -.00002   .0008117    -0.02   0.980                 -.0002653
    other_outpatientservices |  -.0015124   .0006247    -2.42   0.015                 -.0248705
             pharmacy_fills |   .0127133   .0001964    64.72   0.000                  .3261462
                        age |   .0035131   .0001015    34.62   0.000                  .1817838
                  qci_score |   -.003272   .0013323    -2.46   0.014                  -.012897
            engagement_rate |   .0000127   8.06e-06     1.58   0.114                  .0079725
                    rewards |   .0024031   .0000967    24.84   0.000                  .1254386
  --------------------------+------------------------------------------------------------------
  er_visits                 |
                        age |  -.0005016   .0000714    -7.03   0.000                 -.0370254
                  qci_score |   .0035294   .0009371     3.77   0.000                  .0198455
            engagement_rate |  -.0000215   5.67e-06    -3.80   0.000                 -.0192466
                    rewards |  -.0026671    .000068   -39.20   0.000                 -.1986002
```

```
--------------------------+----------------------------------------------------------------
pcp_officevisits          |
                      age |   .0102051   .0009022    11.31   0.000                .0607266
                qci_score |   .0421582    .011844     3.56   0.000                .0191094
          engagement_rate |   2.49e-06   .0000717     0.03   0.972                .0001796
                  rewards |   .0002309   .0008599     0.27   0.788                .0013861
--------------------------+----------------------------------------------------------------
specialist_office_visits  |
                      age |   .0146336   .0013781    10.62   0.000                .0570474
                qci_score |   .0069804   .0180914     0.39   0.700                .0020729
          engagement_rate |   6.21e-06   .0001095     0.06   0.955                .0002929
                  rewards |   .0004574   .0013134     0.35   0.728                .0017986
--------------------------+----------------------------------------------------------------
other_outpatientservices  |
                      age |   .0238391   .0017035    13.99   0.000                .0750126
                qci_score |   .1281599   .0223629     5.73   0.000                .0307186
          engagement_rate |   8.08e-06   .0001353     0.06   0.952                .0003077
                  rewards |   .0006867   .0016235     0.42   0.672                .0021797
--------------------------+----------------------------------------------------------------
pharmacy_fills            |
                      age |    .055894   .0026406    21.17   0.000                .1127401
                qci_score |   .3997155    .034666    11.53   0.000                .0614141
          engagement_rate |   .0000322   .0002097     0.15   0.878                .0007853
                  rewards |   .0038238   .0025167     1.52   0.129                .0077803
-------------------------------------------------------------------------------------------

.
. clear all
```

#SCENARIO 4: Sensitivity analysis 20% lower covariance (S4)

```
.
. ssd init age inpatient_hospitalizations er_visits pcp_officevisits specialist_office_visits other_outpatientservices
pharmacy_fills qci_score mpr pdc rewards engagement_rate

Summary statistics data initialized.  Next use, in any order,
```

```
    ssd set observations (required)
        It is best to do this first.

    ssd set means (optional)
        Default setting is 0.

    ssd set variances or ssd set sd (optional)
        Use this only if you have set or will set correlations and, even then, this is optional but highly recommended.
Default setting is 1.

    ssd set covariances or ssd set correlations (required)

.
. ssd set observations 37359
  (value set)

    Status:
                       observations:    set
                              means:  unset
                    variances or sd:  unset
        covariances or correlations:  unset (required to be set)

.
. ssd set means 49.64 0.04 0.02 2.18 2.61 2.22 9.95 1.47 0.58 0.59 30 234
  (values set)

    Status:
                       observations:    set
                              means:    set
                    variances or sd:  unset
        covariances or correlations:  unset (required to be set)

.
. ssd set covariances 221.105233\0.089650 0.067846\-0.094017 0.011868 0.040578\2.451662 0.037385 0.033620
6.244202\3.267802 0.033255 0.022245 6.805591 14.548804\ 5.864529 0.113002 0.075032 7.918511 15
> .837080 22.331055\ 14.209445 0.200045 0.084031 4.385411 7.174931 10.065882 54.346633\ 4.632682 0.036237 0.002199
0.1013640 .076749 0.274862 0.771759 1.282943\ 0.803770 0.000065 -0.001100 0.087459 0.
> 131478 0.182628 1.037027 0.018688 0.129239\ 0.761121 -0.000166 -0.000795 0.047573 0.073374 0.105173 0.708629 0.012082
0.062483 0.082140\-0.2 -0.8 -0.6 0.05 0.1 0.15 0.85 0.002 0.56 0.48 225\-0.1 -.6
```

```
>  -0.7 0.08 0.2 0.26 1.04 0.001 0.4 0.368 0.82 32400
  (values set)

    Status:
                      observations:    set
                             means:    set
                    variances or sd:    set
        covariances or correlations:    set
```

MODEL 15

```
.
. sem (inpatient_hospitalizations -> mpr, ) (er_visits -> mpr, ) (pcp_officevisits -> mpr, ) (specialist_office_visits
-> mpr, ) (other_outpatientservices -> mpr, ) (pharmacy_fills -> mpr, ) (age -> m
> pr, ) (age -> inpatient_hospitalizations, ) (age -> er_visits, ) (age -> pcp_officevisits, ) (age ->
specialist_office_visits, ) (age -> other_outpatientservices, ) (age -> pharmacy_fills, ) (qci_sc
> ore -> mpr, ) (qci_score -> inpatient_hospitalizations, ) (qci_score -> er_visits, ) (qci_score -> pcp_officevisits,
) (qci_score -> specialist_office_visits, ) (qci_score -> other_outpatientservice
> s, ) (qci_score -> pharmacy_fills, ) (engagement_rate -> mpr, ) (engagement_rate -> inpatient_hospitalizations, )
(engagement_rate -> er_visits, ) (engagement_rate -> pcp_officevisits, ) (engagement
> _rate -> specialist_office_visits, ) (engagement_rate -> other_outpatientservices, ) (engagement_rate ->
pharmacy_fills, ) (rewards -> mpr, ) (rewards -> inpatient_hospitalizations, ) (rewards -> er
> _visits, ) (rewards -> pcp_officevisits, ) (rewards -> specialist_office_visits, ) (rewards ->
other_outpatientservices, ) (rewards -> pharmacy_fills, ), standardized cov( age*qci_score age*engageme
> nt_rate qci_score*engagement_rate engagement_rate*rewards rewards*age rewards*qci_score) nocapslatent

Endogenous variables

Observed:  inpatient_hospitalizations mpr er_visits pcp_officevisits specialist_office_visits other_outpatientservices
pharmacy_fills

Exogenous variables

Observed:  age qci_score engagement_rate rewards

Fitting target model:

Iteration 0:   log likelihood = -1044701.9
```

```
Iteration 1:   log likelihood = -1044701.9

Structural equation model                       Number of obs     =     37,359
Estimation method  = ml
Log likelihood     = -1044701.9

----------------------------------------------------------------------------------------------
                             |                 OIM
                Standardized |      Coef.   Std. Err.      z    P>|z|     [95% Conf. Interval]
-----------------------------+----------------------------------------------------------------
Structural                   |
  inpatient_hospitalizations |
                         age |  -.0117148   .0052245    -2.24   0.025    -.0219546   -.0014749
                   qci_score |   .1260713    .005188    24.30   0.000      .115903    .1362397
             engagement_rate |  -.0127361   .0050229    -2.54   0.011    -.0225808   -.0028914
                     rewards |  -.2047775   .0049186   -41.63   0.000    -.2144177   -.1951372
                       _cons |   .4551753   .0212149    21.46   0.000     .4135949    .4967557
  ---------------------------+----------------------------------------------------------------
  mpr                        |
  inpatient_hospitalizations |  -.0169951   .0049682    -3.42   0.001    -.0267327   -.0072576
                   er_visits |  -.0100565   .0049157    -2.05   0.041    -.0196911    -.000422
            pcp_officevisits |   .0141532   .0067869     2.09   0.037     .0008512    .0274551
    specialist_office_visits |  -.0035415   .0106167    -0.33   0.739    -.0243497    .0172668
    other_outpatientservices |  -.0152749    .010118    -1.51   0.131    -.0351058    .0045559
              pharmacy_fills |   .3819794   .0045713    83.56   0.000     .3730198     .390939
                         age |   .1057218   .0049017    21.57   0.000     .0961146    .1153289
                   qci_score |  -.0160523   .0049292    -3.26   0.001    -.0257134   -.0063913
             engagement_rate |   .0054385   .0046943     1.16   0.247    -.0037623    .0146392
                     rewards |   .0953783   .0048527    19.65   0.000     .0858671    .1048895
                       _cons |   .5660706   .0216887    26.10   0.000     .5235616    .6085796
  ---------------------------+----------------------------------------------------------------
  er_visits                  |
                         age |  -.0370254   .0052662    -7.03   0.000    -.0473469   -.0267039
                   qci_score |   .0198455   .0052685     3.77   0.000     .0095195    .0301715
             engagement_rate |  -.0192466   .0050652    -3.80   0.000    -.0291743    -.009319
                     rewards |  -.1986002   .0049654   -40.00   0.000    -.2083321   -.1888682
                       _cons |   .6193624   .0212275    29.18   0.000     .5777573    .6609675
  ---------------------------+----------------------------------------------------------------
  pcp_officevisits           |
                         age |   .0607266   .0053595    11.33   0.000     .0502222     .071231
```

qci_score	.0191094	.0053677	3.56	0.000	.0085888	.02963
engagement_rate	.0001796	.0051616	0.03	0.972	-.0099369	.0102961
rewards	.0013861	.0051616	0.27	0.788	-.0087304	.0115026
_cons	.6418804	.0222031	28.91	0.000	.5983632	.6853977
specialist_office_visits						
age	.0570474	.0053642	10.63	0.000	.0465337	.0675611
qci_score	.0020729	.0053723	0.39	0.700	-.0084567	.0126024
engagement_rate	.0002929	.0051651	0.06	0.955	-.0098305	.0104163
rewards	.0017986	.0051651	0.35	0.728	-.0083248	.011922
_cons	.487163	.0221029	22.04	0.000	.4438422	.5304838
other_outpatientservices						
age	.0750126	.0053462	14.03	0.000	.0645342	.085491
qci_score	.0307186	.0053578	5.73	0.000	.0202174	.0412197
engagement_rate	.0003077	.0051534	0.06	0.952	-.0097928	.0104081
rewards	.0021797	.0051534	0.42	0.672	-.0079207	.0122801
_cons	.1747415	.0219364	7.97	0.000	.1317469	.2177361
pharmacy_fills						
age	.1127401	.005295	21.29	0.000	.1023621	.1231181
qci_score	.0614141	.0053171	11.55	0.000	.0509929	.0718354
engagement_rate	.0007853	.0051208	0.15	0.878	-.0092513	.0108219
rewards	.0077803	.0051206	1.52	0.129	-.0022559	.0178166
_cons	.877058	.0224515	39.06	0.000	.8330539	.9210621
mean(age)	3.338398	.0132637	251.69	0.000	3.312401	3.364394
mean(qci_score)	1.297835	.0070221	184.82	0.000	1.284072	1.311598
mean(engagement_rate)	1.300017	.0070275	184.99	0.000	1.286244	1.313791
mean(rewards)	2.000027	.0089612	223.19	0.000	1.982463	2.01759
var(e.inpatient_hospitaliz~s)	.9426941	.0023351			.9381285	.9472819
var(e.mpr)	.8228784	.0037268			.8156063	.8302153
var(e.er_visits)	.9588389	.0020129			.9549018	.9627922
var(e.pcp_officevisits)	.9953069	.0007055			.9939251	.9966907
var(e.specialist_office_vi~s)	.9966731	.0005948			.9955079	.9978397
var(e.other_outpatientserv~s)	.9921573	.0009092			.9903769	.9939408
var(e.pharmacy_fills)	.9796493	.0014461			.9768192	.9824877
var(age)	1	.			.	.
var(qci_score)	1	.			.	.

```
         var(engagement_rate)|          1          .                              .           .
                 var(rewards)|          1          .                              .           .
-----------------------------+----------------------------------------------------------------
           cov(age,qci_score)|   .2750612   .0047823    57.52   0.000     .2656881    .2844343
     cov(age,engagement_rate)|  -.0000374   .0051737    -0.01   0.994    -.0101777    .0101029
             cov(age,rewards)|  -.0008967   .0051737    -0.17   0.862     -.011037    .0092436
               cov(qci_score,|
             engagement_rate)|   4.90e-06   .0051737     0.00   0.999    -.0101354    .0101452
       cov(qci_score,rewards)|   .0001177   .0051737     0.02   0.982    -.0100226     .010258
 cov(engagement_rate,rewards)|   .0003037   .0051737     0.06   0.953    -.0098366     .010444
----------------------------------------------------------------------------------------------
Note: The LR test of model vs. saturated is not reported because the fitted
      model is not full rank.

.
. estat gof, stats(all)

----------------------------------------------------------------------------
Fit statistic        |      Value   Description
---------------------+------------------------------------------------------
Likelihood ratio     |
          chi2_ms(.) |          .   model vs. saturated
            p > chi2 |          .
         chi2_bs(49) | 100674.259   baseline vs. saturated
            p > chi2 |      0.000
---------------------+------------------------------------------------------
Population error     |
               RMSEA |          .   Root mean squared error of approximation
 90% CI, lower bound |      0.000
         upper bound |          .
              pclose |          .   Probability RMSEA <= 0.05
---------------------+------------------------------------------------------
Information criteria |
                 AIC |  2.090e+06   Akaike's information criterion
                 BIC |  2.090e+06   Bayesian information criterion
---------------------+------------------------------------------------------
Baseline comparison  |
                 CFI |      1.000   Comparative fit index
                 TLI |          .   Tucker-Lewis index
---------------------+------------------------------------------------------
```

```
Size of residuals    |
                SRMR |      0.174   Standardized root mean squared residual
                  CD |      0.146   Coefficient of determination
----------------------------------------------------------------------------

.
. estat teffects, standardized


Direct effects
----------------------------------------------------------------------------------------------
                             |                 OIM
                             |      Coef.   Std. Err.      z    P>|z|               Std. Coef.
-----------------------------+----------------------------------------------------------------
Structural                   |
  inpatient_hospitalizations |
                         age |  -.0002052   .0000915    -2.24   0.025               -.0117148
                   qci_score |   .0289918   .0012015    24.13   0.000                .1260713
             engagement_rate |  -.0000184   7.27e-06    -2.54   0.011               -.0127361
                     rewards |  -.0035559   .0000872   -40.77   0.000               -.2047775
  ---------------------------+----------------------------------------------------------------
  mpr                        |
  inpatient_hospitalizations |  -.0234952   .0068719    -3.42   0.001               -.0169951
                   er_visits |   -.017977   .0087881    -2.05   0.041               -.0100565
            pcp_officevisits |   .0020395    .000978     2.09   0.037                .0141532
    specialist_office_visits |  -.0003343   .0010023    -0.33   0.739               -.0035415
    other_outpatientservices |   -.001164   .0007714    -1.51   0.131               -.0152749
              pharmacy_fills |   .0186582   .0002426    76.92   0.000                .3819794
                         age |   .0025602   .0001192    21.47   0.000                .1057218
                   qci_score |  -.0051033   .0015671    -3.26   0.001               -.0160523
             engagement_rate |   .0000109   9.39e-06     1.16   0.247                .0054385
                     rewards |   .0022897   .0001168    19.60   0.000                .0953783
  ---------------------------+----------------------------------------------------------------
  er_visits                  |
                         age |  -.0005016   .0000714    -7.03   0.000               -.0370254
                   qci_score |   .0035294   .0009371     3.77   0.000                .0198455
             engagement_rate |  -.0000215   5.67e-06    -3.80   0.000               -.0192466
                     rewards |  -.0026671    .000068   -39.20   0.000               -.1986002
  ---------------------------+----------------------------------------------------------------
  pcp_officevisits           |
```

```
                      age |   .0102051   .0009022    11.31   0.000                 .0607266
                qci_score |   .0421582    .011844     3.56   0.000                 .0191094
          engagement_rate |   2.49e-06   .0000717     0.03   0.972                 .0001796
                  rewards |   .0002309   .0008599     0.27   0.788                 .0013861
  ------------------------+----------------------------------------------------------------
  specialist_office_visits   |
                      age |   .0146336   .0013781    10.62   0.000                 .0570474
                qci_score |   .0069804   .0180914     0.39   0.700                 .0020729
          engagement_rate |   6.21e-06   .0001095     0.06   0.955                 .0002929
                  rewards |   .0004574   .0013134     0.35   0.728                 .0017986
  ------------------------+----------------------------------------------------------------
  other_outpatientservices   |
                      age |   .0238391   .0017035    13.99   0.000                 .0750126
                qci_score |   .1281599   .0223629     5.73   0.000                 .0307186
          engagement_rate |   8.08e-06   .0001353     0.06   0.952                 .0003077
                  rewards |   .0006867   .0016235     0.42   0.672                 .0021797
  ------------------------+----------------------------------------------------------------
  pharmacy_fills             |
                      age |    .055894   .0026406    21.17   0.000                 .1127401
                qci_score |   .3997155    .034666    11.53   0.000                 .0614141
          engagement_rate |   .0000322   .0002097     0.15   0.878                 .0007853
                  rewards |   .0038238   .0025167     1.52   0.129                 .0077803
------------------------------------------------------------------------------------------


Indirect effects
------------------------------------------------------------------------------------------
                             |                OIM
                             |      Coef.   Std. Err.      z    P>|z|             Std. Coef.
-----------------------------+------------------------------------------------------------
Structural                   |
  inpatient_hospitalizations |
                      age |          0  (no path)                                        0
                qci_score |          0  (no path)                                        0
          engagement_rate |          0  (no path)                                        0
                  rewards |          0  (no path)                                        0
  ---------------------------+------------------------------------------------------------
  mpr                        |
  inpatient_hospitalizations |          0  (no path)                                        0
                er_visits |          0  (no path)                                        0
```

```
          pcp_officevisits |          0  (no path)                                              0
  specialist_office_visits |          0  (no path)                                              0
  other_outpatientservices |          0  (no path)                                              0
             pharmacy_fills |          0  (no path)                                              0
                        age |   .0010449   .0000518    20.17   0.000                     .0431475
                  qci_score |   .0066478   .0006842     9.72   0.000                     .0209106
            engagement_rate |   1.41e-06   3.93e-06     0.36   0.719                     .0007068
                    rewards |   .0002024   .0000563     3.59   0.000                     .0084293
  --------------------------+----------------------------------------------------------------
  er_visits                 |
                        age |          0  (no path)                                              0
                  qci_score |          0  (no path)                                              0
            engagement_rate |          0  (no path)                                              0
                    rewards |          0  (no path)                                              0
  --------------------------+----------------------------------------------------------------
  pcp_officevisits          |
                        age |          0  (no path)                                              0
                  qci_score |          0  (no path)                                              0
            engagement_rate |          0  (no path)                                              0
                    rewards |          0  (no path)                                              0
  --------------------------+----------------------------------------------------------------
  specialist_office_visits  |
                        age |          0  (no path)                                              0
                  qci_score |          0  (no path)                                              0
            engagement_rate |          0  (no path)                                              0
                    rewards |          0  (no path)                                              0
  --------------------------+----------------------------------------------------------------
  other_outpatientservices  |
                        age |          0  (no path)                                              0
                  qci_score |          0  (no path)                                              0
            engagement_rate |          0  (no path)                                              0
                    rewards |          0  (no path)                                              0
  --------------------------+----------------------------------------------------------------
  pharmacy_fills            |
                        age |          0  (no path)                                              0
                  qci_score |          0  (no path)                                              0
            engagement_rate |          0  (no path)                                              0
                    rewards |          0  (no path)                                              0
---------------------------------------------------------------------------------------------
```

Total effects

	Coef.	OIM Std. Err.	z	P>|z|	Std. Coef.
**Structural**					
inpatient_hospitalizations					
age	-.0002052	.0000915	-2.24	0.025	-.0117148
qci_score	.0289918	.0012015	24.13	0.000	.1260713
engagement_rate	-.0000184	7.27e-06	-2.54	0.011	-.0127361
rewards	-.0035559	.0000872	-40.77	0.000	-.2047775
mpr					
inpatient_hospitalizations	-.0234952	.0068719	-3.42	0.001	-.0169951
er_visits	-.017977	.0087881	-2.05	0.041	-.0100565
pcp_officevisits	.0020395	.000978	2.09	0.037	.0141532
specialist_office_visits	-.0003343	.0010023	-0.33	0.739	-.0035415
other_outpatientservices	-.001164	.0007714	-1.51	0.131	-.0152749
pharmacy_fills	.0186582	.0002426	76.92	0.000	.3819794
age	.0036051	.0001281	28.14	0.000	.1488693
qci_score	.0015445	.001682	0.92	0.358	.0048583
engagement_rate	.0000123	.0000102	1.21	0.227	.0061452
rewards	.002492	.0001221	20.41	0.000	.1038076
er_visits					
age	-.0005016	.0000714	-7.03	0.000	-.0370254
qci_score	.0035294	.0009371	3.77	0.000	.0198455
engagement_rate	-.0000215	5.67e-06	-3.80	0.000	-.0192466
rewards	-.0026671	.000068	-39.20	0.000	-.1986002
pcp_officevisits					
age	.0102051	.0009022	11.31	0.000	.0607266
qci_score	.0421582	.011844	3.56	0.000	.0191094
engagement_rate	2.49e-06	.0000717	0.03	0.972	.0001796
rewards	.0002309	.0008599	0.27	0.788	.0013861
specialist_office_visits					
age	.0146336	.0013781	10.62	0.000	.0570474
qci_score	.0069804	.0180914	0.39	0.700	.0020729

```
           engagement_rate |   6.21e-06   .0001095     0.06   0.955                  .0002929
                   rewards |   .0004574   .0013134     0.35   0.728                  .0017986
  -------------------------+----------------------------------------------------------------
  other_outpatientservices   |
                       age |   .0238391   .0017035    13.99   0.000                  .0750126
                 qci_score |   .1281599   .0223629     5.73   0.000                  .0307186
           engagement_rate |   8.08e-06   .0001353     0.06   0.952                  .0003077
                   rewards |   .0006867   .0016235     0.42   0.672                  .0021797
  -------------------------+----------------------------------------------------------------
  pharmacy_fills           |
                       age |    .055894   .0026406    21.17   0.000                  .1127401
                 qci_score |   .3997155    .034666    11.53   0.000                  .0614141
           engagement_rate |   .0000322   .0002097     0.15   0.878                  .0007853
                   rewards |   .0038238   .0025167     1.52   0.129                  .0077803
--------------------------------------------------------------------------------------------
```

. MODEL 16

```
. sem (inpatient_hospitalizations -> pdc, ) (er_visits -> pdc, ) (pcp_officevisits -> pdc, ) (specialist_office_visits
-> pdc, ) (other_outpatientservices -> pdc, ) (pharmacy_fills -> pdc, ) (age -> p
> dc, ) (age -> inpatient_hospitalizations, ) (age -> er_visits, ) (age -> pcp_officevisits, ) (age ->
specialist_office_visits, ) (age -> other_outpatientservices, ) (age -> pharmacy_fills, ) (qci_sc
> ore -> pdc, ) (qci_score -> inpatient_hospitalizations, ) (qci_score -> er_visits, ) (qci_score -> pcp_officevisits,
) (qci_score -> specialist_office_visits, ) (qci_score -> other_outpatientservice
> s, ) (qci_score -> pharmacy_fills, ) (engagement_rate -> pdc, ) (engagement_rate -> inpatient_hospitalizations, )
(engagement_rate -> er_visits, ) (engagement_rate -> pcp_officevisits, ) (engagement
> _rate -> specialist_office_visits, ) (engagement_rate -> other_outpatientservices, ) (engagement_rate ->
pharmacy_fills, ) (rewards -> pdc, ) (rewards -> inpatient_hospitalizations, ) (rewards -> er
> _visits, ) (rewards -> pcp_officevisits, ) (rewards -> specialist_office_visits, ) (rewards ->
other_outpatientservices, ) (rewards -> pharmacy_fills, ), standardized cov( age*qci_score age*engageme
> nt_rate qci_score*engagement_rate engagement_rate*rewards rewards*age rewards*qci_score) nocapslatent

Endogenous variables

Observed:  inpatient_hospitalizations pdc er_visits pcp_officevisits specialist_office_visits other_outpatientservices
pharmacy_fills

Exogenous variables
```

```
Observed:  age qci_score engagement_rate rewards

Fitting target model:

Iteration 0:   log likelihood = -1036892.2
Iteration 1:   log likelihood = -1036892.2

Structural equation model                       Number of obs     =     37,359
Estimation method  = ml
Log likelihood     = -1036892.2

----------------------------------------------------------------------------------------------
                             |                 OIM
                Standardized |      Coef.   Std. Err.      z    P>|z|     [95% Conf. Interval]
-----------------------------+----------------------------------------------------------------
Structural                   |
  inpatient_hospitalizations |
                         age |  -.0117148   .0052245    -2.24   0.025    -.0219546   -.0014749
                   qci_score |   .1260713    .005188    24.30   0.000      .115903    .1362397
             engagement_rate |  -.0127361   .0050229    -2.54   0.011    -.0225808   -.0028914
                     rewards |  -.2047775   .0049186   -41.63   0.000    -.2144177   -.1951372
                       _cons |   .4551753   .0212149    21.46   0.000     .4135949    .4967557
  ---------------------------+----------------------------------------------------------------
  pdc                        |
  inpatient_hospitalizations |  -.0112931   .0050522    -2.24   0.025    -.0211951    -.001391
                   er_visits |  -.0014081   .0049979    -0.28   0.778    -.0112039    .0083877
           pcp_officevisits |  -.0025879   .0068995    -0.38   0.708    -.0161106    .0109348
    specialist_office_visits |  -.0013915   .0107934    -0.13   0.897    -.0225461    .0197631
    other_outpatientservices |   -.023703   .0102824    -2.31   0.021     -.043856   -.0035499
              pharmacy_fills |   .3265457   .0047558    68.66   0.000     .3172245    .3358669
                         age |   .1467726   .0049615    29.58   0.000     .1370482     .156497
                   qci_score |  -.0307129   .0050103    -6.13   0.000     -.040533   -.0208929
             engagement_rate |   .0066678   .0047724     1.40   0.162     -.002686    .0160215
                     rewards |   .1064365   .0049277    21.60   0.000     .0967783    .1160947
                       _cons |   .9568355   .0227153    42.12   0.000     .9123143    1.001357
  ---------------------------+----------------------------------------------------------------
  er_visits                  |
                         age |  -.0370254   .0052662    -7.03   0.000    -.0473469   -.0267039
                   qci_score |   .0198455   .0052685     3.77   0.000     .0095195    .0301715
```

engagement_rate	-.0192466	.0050652	-3.80	0.000	-.0291743	-.009319
rewards	-.1986002	.0049654	-40.00	0.000	-.2083321	-.1888682
_cons	.6193624	.0212275	29.18	0.000	.5777573	.6609675
pcp_officevisits						
age	.0607266	.0053595	11.33	0.000	.0502222	.071231
qci_score	.0191094	.0053677	3.56	0.000	.0085888	.02963
engagement_rate	.0001796	.0051616	0.03	0.972	-.0099369	.0102961
rewards	.0013861	.0051616	0.27	0.788	-.0087304	.0115026
_cons	.6418804	.0222031	28.91	0.000	.5983632	.6853977
specialist_office_visits						
age	.0570474	.0053642	10.63	0.000	.0465337	.0675611
qci_score	.0020729	.0053723	0.39	0.700	-.0084567	.0126024
engagement_rate	.0002929	.0051651	0.06	0.955	-.0098305	.0104163
rewards	.0017986	.0051651	0.35	0.728	-.0083248	.011922
_cons	.487163	.0221029	22.04	0.000	.4438422	.5304838
other_outpatientservices						
age	.0750126	.0053462	14.03	0.000	.0645342	.085491
qci_score	.0307186	.0053578	5.73	0.000	.0202174	.0412197
engagement_rate	.0003077	.0051534	0.06	0.952	-.0097928	.0104081
rewards	.0021797	.0051534	0.42	0.672	-.0079207	.0122801
_cons	.1747415	.0219364	7.97	0.000	.1317469	.2177361
pharmacy_fills						
age	.1127401	.005295	21.29	0.000	.1023621	.1231181
qci_score	.0614141	.0053171	11.55	0.000	.0509929	.0718354
engagement_rate	.0007853	.0051208	0.15	0.878	-.0092513	.0108219
rewards	.0077803	.0051206	1.52	0.129	-.0022559	.0178166
_cons	.877058	.0224515	39.06	0.000	.8330539	.9210621
mean(age)	3.338398	.0132637	251.69	0.000	3.312401	3.364394
mean(qci_score)	1.297835	.0070221	184.82	0.000	1.284072	1.311598
mean(engagement_rate)	1.300017	.0070275	184.99	0.000	1.286244	1.313791
mean(rewards)	2.000027	.0089612	223.19	0.000	1.982463	2.01759
var(e.inpatient_hospitaliz~s)	.9426941	.0023351			.9381285	.9472819
var(e.pdc)	.8504881	.0035554			.8435481	.8574852
var(e.er_visits)	.9588389	.0020129			.9549018	.9627922

```
      var(e.pcp_officevisits)|   .9953069   .0007055                      .9939251    .9966907
var(e.specialist_office_vi~s)|   .9966731   .0005948                      .9955079    .9978397
var(e.other_outpatientserv~s)|   .9921573   .0009092                      .9903769    .9939408
        var(e.pharmacy_fills)|   .9796493   .0014461                      .9768192    .9824877
                     var(age)|          1          .                             .           .
               var(qci_score)|          1          .                             .           .
         var(engagement_rate)|          1          .                             .           .
                 var(rewards)|          1          .                             .           .
-----------------------------+----------------------------------------------------------------
           cov(age,qci_score)|   .2750612   .0047823    57.52   0.000     .2656881    .2844343
     cov(age,engagement_rate)|  -.0000374   .0051737    -0.01   0.994    -.0101777    .0101029
             cov(age,rewards)|  -.0008967   .0051737    -0.17   0.862     -.011037    .0092436
               cov(qci_score,|
             engagement_rate)|   4.90e-06   .0051737     0.00   0.999    -.0101354    .0101452
       cov(qci_score,rewards)|   .0001177   .0051737     0.02   0.982    -.0100226     .010258
 cov(engagement_rate,rewards)|   .0003037   .0051737     0.06   0.953    -.0098366     .010444
----------------------------------------------------------------------------------------------
Note: The LR test of model vs. saturated is not reported because the fitted
      model is not full rank.

.
. estat gof, stats(all)

----------------------------------------------------------------------------
Fit statistic        |      Value   Description
---------------------+------------------------------------------------------
Likelihood ratio     |
          chi2_ms(.) |          .   model vs. saturated
            p > chi2 |          .
         chi2_bs(49) |  99361.144   baseline vs. saturated
            p > chi2 |      0.000
---------------------+------------------------------------------------------
Population error     |
               RMSEA |          .   Root mean squared error of approximation
 90% CI, lower bound |      0.000
         upper bound |          .
              pclose |          .   Probability RMSEA <= 0.05
---------------------+------------------------------------------------------
Information criteria |
                 AIC |  2.074e+06   Akaike's information criterion
```

```
                BIC |  2.074e+06   Bayesian information criterion
---------------------+------------------------------------------------------
Baseline comparison  |
                CFI |      1.000   Comparative fit index
                TLI |          .   Tucker-Lewis index
---------------------+------------------------------------------------------
Size of residuals    |
               SRMR |      0.173   Standardized root mean squared residual
                 CD |      0.156   Coefficient of determination
----------------------------------------------------------------------------

.
. estat teffects, standardized


Direct effects
----------------------------------------------------------------------------------------------
                             |                 OIM
                             |      Coef.   Std. Err.      z    P>|z|               Std. Coef.
-----------------------------+----------------------------------------------------------------
Structural                   |
  inpatient_hospitalizations |
                         age |  -.0002052   .0000915    -2.24   0.025                -.0117148
                   qci_score |   .0289918   .0012015    24.13   0.000                 .1260713
             engagement_rate |  -.0000184   7.27e-06    -2.54   0.011                -.0127361
                     rewards |  -.0035559   .0000872   -40.77   0.000                -.2047775
  ---------------------------+----------------------------------------------------------------
  pdc                        |
  inpatient_hospitalizations |  -.0124599   .0055756    -2.23   0.025                -.0112931
                   er_visits |  -.0020089   .0071303    -0.28   0.778                -.0014081
            pcp_officevisits |  -.0002976   .0007935    -0.38   0.708                -.0025879
    specialist_office_visits |  -.0001048   .0008132    -0.13   0.897                -.0013915
    other_outpatientservices |  -.0014415   .0006258    -2.30   0.021                 -.023703
              pharmacy_fills |   .0127298   .0001968    64.68   0.000                 .3265457
                         age |   .0028367   .0000967    29.32   0.000                 .1467726
                   qci_score |  -.0077926   .0012715    -6.13   0.000                -.0307129
             engagement_rate |   .0000106   7.62e-06     1.40   0.162                 .0066678
                     rewards |   .0020392   .0000948    21.52   0.000                 .1064365
  ---------------------------+----------------------------------------------------------------
  er_visits                  |
```

age	-.0005016	.0000714	-7.03	0.000	-.0370254
qci_score	.0035294	.0009371	3.77	0.000	.0198455
engagement_rate	-.0000215	5.67e-06	-3.80	0.000	-.0192466
rewards	-.0026671	.000068	-39.20	0.000	-.1986002
pcp_officevisits					
age	.0102051	.0009022	11.31	0.000	.0607266
qci_score	.0421582	.011844	3.56	0.000	.0191094
engagement_rate	2.49e-06	.0000717	0.03	0.972	.0001796
rewards	.0002309	.0008599	0.27	0.788	.0013861
specialist_office_visits					
age	.0146336	.0013781	10.62	0.000	.0570474
qci_score	.0069804	.0180914	0.39	0.700	.0020729
engagement_rate	6.21e-06	.0001095	0.06	0.955	.0002929
rewards	.0004574	.0013134	0.35	0.728	.0017986
other_outpatientservices					
age	.0238391	.0017035	13.99	0.000	.0750126
qci_score	.1281599	.0223629	5.73	0.000	.0307186
engagement_rate	8.08e-06	.0001353	0.06	0.952	.0003077
rewards	.0006867	.0016235	0.42	0.672	.0021797
pharmacy_fills					
age	.055894	.0026406	21.17	0.000	.1127401
qci_score	.3997155	.034666	11.53	0.000	.0614141
engagement_rate	.0000322	.0002097	0.15	0.878	.0007853
rewards	.0038238	.0025167	1.52	0.129	.0077803

Indirect effects

		OIM			
	Coef.	Std. Err.	z	P>|z|	Std. Coef.
Structural					
inpatient_hospitalizations					
age	0	(no path)			0
qci_score	0	(no path)			0

```
           engagement_rate |          0  (no path)                                               0
                   rewards |          0  (no path)                                               0
---------------------------+----------------------------------------------------------------
pdc                        |
inpatient_hospitalizations |          0  (no path)                                               0
                 er_visits |          0  (no path)                                               0
          pcp_officevisits |          0  (no path)                                               0
  specialist_office_visits |          0  (no path)                                               0
  other_outpatientservices |          0  (no path)                                               0
            pharmacy_fills |          0  (no path)                                               0
                       age |   .0006761    .000036    18.78   0.000                        .0349847
                 qci_score |   .0045219   .0004767     9.49   0.000                        .0178224
           engagement_rate |   6.69e-07   2.68e-06     0.25   0.803                        .0004192
                   rewards |   .0000972   .0000407     2.39   0.017                        .0050751
---------------------------+----------------------------------------------------------------
er_visits                  |
                       age |          0  (no path)                                               0
                 qci_score |          0  (no path)                                               0
           engagement_rate |          0  (no path)                                               0
                   rewards |          0  (no path)                                               0
---------------------------+----------------------------------------------------------------
pcp_officevisits           |
                       age |          0  (no path)                                               0
                 qci_score |          0  (no path)                                               0
           engagement_rate |          0  (no path)                                               0
                   rewards |          0  (no path)                                               0
---------------------------+----------------------------------------------------------------
specialist_office_visits   |
                       age |          0  (no path)                                               0
                 qci_score |          0  (no path)                                               0
           engagement_rate |          0  (no path)                                               0
                   rewards |          0  (no path)                                               0
---------------------------+----------------------------------------------------------------
other_outpatientservices   |
                       age |          0  (no path)                                               0
                 qci_score |          0  (no path)                                               0
           engagement_rate |          0  (no path)                                               0
                   rewards |          0  (no path)                                               0
---------------------------+----------------------------------------------------------------
pharmacy_fills             |
```

age	0	(no path)			0
qci_score	0	(no path)			0
engagement_rate	0	(no path)			0
rewards	0	(no path)			0

Total effects

	Coef.	OIM Std. Err.	z	P>|z|	Std. Coef.
**Structural**					
inpatient_hospitalizations					
age	-.0002052	.0000915	-2.24	0.025	-.0117148
qci_score	.0289918	.0012015	24.13	0.000	.1260713
engagement_rate	-.0000184	7.27e-06	-2.54	0.011	-.0127361
rewards	-.0035559	.0000872	-40.77	0.000	-.2047775
pdc					
inpatient_hospitalizations	-.0124599	.0055756	-2.23	0.025	-.0112931
er_visits	-.0020089	.0071303	-0.28	0.778	-.0014081
pcp_officevisits	-.0002976	.0007935	-0.38	0.708	-.0025879
specialist_office_visits	-.0001048	.0008132	-0.13	0.897	-.0013915
other_outpatientservices	-.0014415	.0006258	-2.30	0.021	-.023703
pharmacy_fills	.0127298	.0001968	64.68	0.000	.3265457
age	.0035128	.0001017	34.55	0.000	.1817573
qci_score	-.0032706	.0013347	-2.45	0.014	-.0128906
engagement_rate	.0000113	8.07e-06	1.40	0.161	.007087
rewards	.0021364	.0000969	22.05	0.000	.1115116
er_visits					
age	-.0005016	.0000714	-7.03	0.000	-.0370254
qci_score	.0035294	.0009371	3.77	0.000	.0198455
engagement_rate	-.0000215	5.67e-06	-3.80	0.000	-.0192466
rewards	-.0026671	.000068	-39.20	0.000	-.1986002
pcp_officevisits					
age	.0102051	.0009022	11.31	0.000	.0607266
qci_score	.0421582	.011844	3.56	0.000	.0191094

engagement_rate	2.49e-06	.0000717	0.03	0.972	.0001796
rewards	.0002309	.0008599	0.27	0.788	.0013861
specialist_office_visits					
age	.0146336	.0013781	10.62	0.000	.0570474
qci_score	.0069804	.0180914	0.39	0.700	.0020729
engagement_rate	6.21e-06	.0001095	0.06	0.955	.0002929
rewards	.0004574	.0013134	0.35	0.728	.0017986
other_outpatientservices					
age	.0238391	.0017035	13.99	0.000	.0750126
qci_score	.1281599	.0223629	5.73	0.000	.0307186
engagement_rate	8.08e-06	.0001353	0.06	0.952	.0003077
rewards	.0006867	.0016235	0.42	0.672	.0021797
pharmacy_fills					
age	.055894	.0026406	21.17	0.000	.1127401
qci_score	.3997155	.034666	11.53	0.000	.0614141
engagement_rate	.0000322	.0002097	0.15	0.878	.0007853
rewards	.0038238	.0025167	1.52	0.129	.0077803
